
JOURNAL INFORMATION
==============================
NLM Title Abbreviation: J Orthop Traumatol
Journal ID: J Orthop Traumatol
NLM Journal ID: 101090931

Journal of Orthopaedics and Traumatology : Official Journal of the Italian Society of Orthopaedics and Traumatology

ISSN: 1590-9921
EISSN: 1590-9999
Publisher: Springer

ARTICLE INFORMATION
==============================
PMCID: PMC4417793
DOI: 10.1007/s10195-013-0259-6
Article ID: 259
Article version: 1
Subjects: Events

In-Depth Oral Presentations and Oral Communications

Electronic publication date: 2013 Sep 18
Print publication date: 2013 Oct
Volume: 14
Issue: Suppl 1
First page: 47
Last page: 82
Copyright: © The Author(s) 2013
License: Open AccessThis article is distributed under the terms of the Creative Commons Attribution 2.0 International License (https://creativecommons.org/licenses/by/2.0), which permits unrestricted use, distribution, and reproduction in any medium, provided the original work is properly cited.
License URL: https://creativecommons.org/licenses/by/2.0/

Keywords: Anterior Cruciate Ligament, Anterior Cruciate Ligament Reconstruction, Proximal Humeral Fracture, Open Wedge High Tibial Osteotomy, Direct Anterior Approach
issue-copyright-statement: © The Author(s) 2013

==============================
J Orthopaed Traumatol (2013) 14 (Suppl 1):S47–S82
DOI 10.1007/s10195-013-0259-6

28 OCTOBER 2013

### In-Depth Oral Presentations and Oral Communications

### IN DEPTH ORAL PRESENTATIONS

### *A06–BIOMATERIALS*

### Titanium plates with angular stability, the removal could be a problem

R. Matteotti*[1], A. Martinasso[2], E. Grosso[3], R. Sisto[3], L. Rollero[2], D. Testa[3], B. Battiston[3]

[1]Ospedale Regionale Umberto Parini (Aosta, IT);
[2]Università degli Studi di Torino (Turin, IT);
[3]Traumatologia Muscolo-Scheletrica CTO (Turin, IT)

**Introduction** Titanium plates with locking screws are certainly a very useful tool for the surgeon especially in traumatology. Often the hardware removal is difficult.
**Materials and methods** The aim of this study is the assessment of the incidence and type of difficulty of removing the locking compression plate in our experience. One hundred and fifty seven titanium plates implanted in our center were removed from January 2010 to December 2011. First surgery was performed by experienced trauma surgeons and the material placement rules respected, particularly drilling and screwing in the axis, perpendicular to the plate. Targeting device was systematically used with no resistance, in association with torque controlled screwdrivers.
**Results** Thirty-three patients (21 %) out of 157 had some problems: destruction of the recess of the screw head already present, or caused by surgeon during removal; screws jamming on the plate. These problems are more frequent with minimally invasive surgery, and in patients operated at least after 2 years. Surgery in these cases lasted on average 43 min more.
**Conclusions** Our study shows that prevention is essential but it is not sufficient; anyway many problems can occur even in patients surgically treated following the rigorous surgical technique. These problems are more frequent with minimally invasive surgery and at least after 2 years from the first surgery.

### *In vivo* experimental study of a new porous cement: research line

C. Dall'Oca*, T. Maluta, B. Magnan

Azienda Ospedaliera Universitaria Integrata (Verona, IT)

**Introduction** Finding a biocompatible material, which, for its chemical-structural characteristics, promised a biomechanical strength adequate till to its complete re-habitation by the neo-formed bone tissue, the need was felt. The purpose of this study is the analysis of a new acrylic bone cement (Porosectan) based on polymethylmethacrylate (PMMA) and β-tricalcium phosphate (TCP).
**Materials and methods** From May 2007 to now at the Department of Orthopaedics and Traumatology, University of Verona, we have tested the new porous cement into 3 phases: the first, on which it was evaluated the biocompatibility on 8 New Zealand rabbits, the second, in which we have analyzed osteoconduction and osteointegration on 12 New Zealand rabbits and the third, to test the biomechanical strength on large animals, 5 pigs. For each sample analyzes were conducted macroscopic, microscopic with suitable colors for bone, X-ray and electron microscopy (SEM).
**Results** The first phase of the study has demonstrated the excellent biocompatibility of the cement due to the low polymerization temperature and a valid osteointegration at the cement-bone contact surface. The second phase of the study showed that the Porosectan was characterized by macroscopic porosity, with pores of 200–500 μm that create a structure similar to that of trabecular bone. The increase of the contact surface cement-bone and the formation, not only at the interface level, of newly formed trabeculae, demonstrated the excellent osteointegration of this cement. The formation of neotrabeculae within the cement, which involves all the material surface after 12 months, is entirely determined by the resorption of the β-TCP that releases calcium ions and phosphorus, osteoconductive factors. The last phase of the study is providing encouraging results on the biomechanical strength of the Porosectan: the material implanted on the femur and tibia of large animals that were on average 220 kg of weight is plotted periodically with standard radiographs, not showing any signs of slowing down.
**Discussion** The high polymerization temperature and the absence of osseointegration make the PMMA as a foreign body for our body which tends to isolate with the formation of a fibrous layer cement-bone interface. The preliminary results lead us to think that the Porosectan can be a viable alternative to PMMA cements.
**Conclusions** The almost total re-housing by newly formed bone in Porosectan with partial-progressive resorption of the cement, making this an ideal material in the daily practice of orthopaedic surgery. Further studies should be conducted on this material but the applications seem to be promising for the future.

### The biomaterials in the surgical treatment of the lesion of the anterior talofibular ligament: preliminary results

A. Siclari*, M. Piras, T. Aramo, E. Boux

Ospedale Infermi (Biella, IT)

**Introduction** The ankle sprain is one of the most common lesions, being the 25 % of any sports trauma and affecting 1 in 10000 person a day. The 85 % of the sprains affect the lateral ligamental system. The most used treatment for this lesion is the conservative one, but, of all the patients so treated, a percentage that varies from 20 to 40 % is headed to a symptomatic chronic instability characterized by repeated sprain episodes. The patients affected with this syndrome, in the 72 % of the cases, are unable to back to sport on a pre-trauma level or are obliged to change job. The lesion of the anterior talofibular ligament, if a 3rd degree, causes an alteration of the movements of the talus so that, in some cases, the automatic system of proprioceptive compensation is not able to balance; then it appears the chronic instability. Until now the unease of the surgical treatment did not compensate this evolution. The introduction of a new technique which uses the biomaterial to reconstruct the ligament with percutaneous and mini invasive procedure opens a new way in the treatment of this lesion.

**Materials and methods** In 2012, 24 patients (4 professional sportsmen, 14 males and 10 females, age 18–44) with a 3rd degree lesion of the anterior talofibular ligament have been treated with an Artelon Tissue Reinforcement (EON Medica) implant, soaked in autologous PRP with percutaneous technique and fixation with anchors. The load has been allowed immediately and the patients have followed a specific rehabilitative accelerated protocol. The patients have been checked with the following clinical follow-up: weekly check for a month, then monthly for 6 months.

**Results** In no case of the 24 treated has appeared a further sprain. The complete load has been well tolerated by everyone, without pain. Even in the first check the walk was correct. The restart of the agonistic activity in the 4 cases of sportsmen has begun 45 days after the surgery. Currently no complications have appeared.

**Discussion** The high frequency of chronic instability after a 3rd degree lesion of the anterior talofibular ligament treated bloodlessly has lead to the search for new surgical treatments with less unease for the patient. The proposed technique seems to respond to this necessity.

**Conclusions** The preliminary results, even evaluating the small number of treated patients and of the short follow-up, are good and lead us to continue to use this technique and continue the follow-up.

### Reconstruction of severe bone defects in acetabular cup revision by application of an innovative osteoinductive paste, containing demineralized bone matrix (DBM)

G. Pignatti, N. Fazio*, C. Quinto, I. Andreoli, P. Fornasari

Istituto Ortopedico Rizzoli (Bologna, IT)

**Introduction** The most common indications for revision of the acetabular component include aseptic loosening, hip instability, periprosthetic osteolysis, and periprosthetic infection. A successful acetabular revision must provide intimate contact between the acetabular implant and the host bone, allowing bone ingrowth into a cementless acetabular component, a mechanical construct able to distribute physiologic stresses to the surrounding acetabular bone. Acetabular bone defects can be defined as contained or uncontained. Contained or cavitary defects can be filled with autologous or allogenic bone grafts; uncontained or segmental defects can be managed also with bone graft, but the reconstruction is more difficult and usually required a revision type acetabular component.

**Materials and methods** An innovative allogenic bone-based osteoinductive and malleable paste (DB-Graft) has been developed and used in the acetabular revisions, subjects of this study. DB-Graft is composed as follows: (1) lyophilized bone chips—granting osteoconductive property; (2) DBM—delivering osteoinductive biological signalling in situ; (3) equine atelocollagen—working both as paste matrix and as local cells adhesion substrate. DBM consists of cortical bone that has had the inorganic mineral removed; such treatment leaves intact the organic matrix, increasing the bioavailability of the cortical osteoinductive factors, such as bone morphogenetic factors messengers family (BMP-2; BMP-4; BMP-7). DBM is known to be more biologically active than demineralized bone grafts.

**Results** Between January and December 2012, 20 acetabular cup revision were performed, using DB-Graft paste as bone filler. The bone defect was classified according to Paprosky. In no case patients exhibited complications, such as infection, or implant failure. In all the cases radiographs have been taken immediate postoperatively, and after 3, 6 and 12 months. In no case a tissue sample have been taken for histological analysis. In all the analyzed cases, a significant bone deposition could have been observed even 3 months after the DB-Graft implant.

**Discussion** Main goal of the presented clinical study, still ongoing, is the clinical and radiographic evaluation of the acetabular revision technique performed adopting uncemented acetabular components, associated with the in situ DB-Graft osteoinductive paste implant. To date, the engineered tissue DB-Graft is showing a good osteogenic potential.

**Conclusions** The addition of DBM as a bone filler, and thus of the physiological bone osteoinductive factors, could represent an advancement compared to the standard in the reconstruction of the acetabular defect in case of acetabular cup revision.

### *A07–BIOTECHNOLOGIES*

### Bone marrow cells and orthopaedics: where are we?

M. Cavallo*, R. Buda, D.M. Donati, F. Vannini, L. Ramponi, L. Cevolani, S. Maraldi, S. Giannini

Istituto Ortopedico Rizzoli (Bologna, IT)

**Introduction** The high regenerative potential and the capacity of differentiation of bone marrow mononuclear cells (BMDC) makes them suitable for multiple indications in orthopaedic pathology, especially where injured osseous or chondral tissue are not brightly healing. Aim of this work is to describe the different clinical applications of these cells with both harvesting and implantation in a single step technique.

**Materials and methods** Between 2006 and 2010, 298 patients underwent a regenerative treatment with BMDC. One hundred and twenty-eight were osteochondral lesions of the talus, 52 osteochondral lesions of the knee, 66 simple bone cysts, 38 femoral head osteonecrosis and 14 upper or lower limb nonunions. BMDC were harvested and concentrated in operative room and delivered into the lesion site with a collagen or hyaluronic acid biomaterial and platelet gel. In the deeper lesions, in the bone cysts, nonunion and femoral head osteonecrosis there were supplementation of demineralised bone matrix.

**Results** Osteochondral lesions obtained good clinical, imaging and histological results. In particular AOFAS, IKDC and KOOS scores improved significatively, T2 mapping MRI and bioptic samples showed a good quality of the regenerated cartilage. The treatment of bone cists reported a 77 % of success rate with significative improvements in Neer's index. 22 % of the cases needed multiple treatments and the 12 % reported a pathological fracture. For the femoral head osteonecrosis results are satisfactory in more than 95 % of the cases; patients with Ficat stage II of the necrosis have shown a better clinical response than patients with stage III-IV. In three cases the treatment failed. After 3 months from surgery, 7 patients bore full load, did not feel pain and X-ray showed an increase of osteogenesis which permitted the removal of the external fixator in 3 patients. 2 cases failed; in one of them the patient underwent another BMDC treatment and in one the fixation was revised.

**Discussion** The clinical application of BMDC in regenerative orthopaedic surgery appears to date a valid option for the treatment of osteochondral lesion of knee and ankle, femoral head osteonecrosis, upper and lower limb bone cysts and non-unions with allowing a considerable reduction in the morbidity of patients and costs.

**Conclusions** One step technique has proved to be a promising treatment in this kind of orthopaedic diseases. Nevertheless a longer follow-up is necessary, in order to confirm these results and to enlarge the indications of this procedure.

### Knee cartilage lesions treated with the AMIC technique: 198 cases after 2 years

A. Siclari*, M. Piras, T. Aramo, M. Vigna Suria, E. Boux

Ospedale Infermi (Biella, IT)

**Introduction** The treatment of cartilage lesions is a complicated problem for the orthopaedic surgeon, but the use of the reparative potentiality of the pluripotent mesenchymal cells and of the tissue engineering has opened new horizons. The combined use of a scaffold in hyaluronic acid and polyglycolic acid with the grow factors present in the platelet's concentrated lead to the regeneration of the cartilage, thanks to the local induction of chondrogenesis of the mesenchymal cells of the patient.

**Materials and methods** The procedure is entirely arthroscopic. From July 2007 to December 2010 176 patients have been treated with 198 surgeries for chondral lesions of the knee, 123 of the shinbone and 75 of the thighbone. 35 patients had a kissing lesion; the biggest lesion was of 5 $cm^2$. The patients have been checked with the KOOS score in the preoperative state and then in the 3rd, the 6th, the 9th, the 12th and the 24th month.

**Results** The average preoperative score was 50.25 points; at the 24th month it was 82.85 points ($p$ value $< 0.0001$), with an increase of 32.60 points. All the patients are checked with fibroarthroscopy in the 4th, the 8th and in the 12th month. Ten patients have been re-evaluated with a second arthroscopy 9 months after the first implant and biopsy of the neoformed cartilage have been carried out. The arthroscopic aspect was of a cartilage tissue, whiter than the other cartilage, smooth but with some undulation, of a consistency similar to the normal, well stick to the bone. The histological exam has proven in all 20 cases the complete disappearance of the scaffold and the presence of a neo-cartilage close to hyaline cartilage.

**Discussion** One patient has been treated with an arthroscopy after 24 months from the implant; it has showed, on a macroscopic aspect, a flat cartilage, of normal consistency, with a colour only a little darker than the nearest cartilage. The bioptic exam has proven the presence of hyaline cartilage.

**Conclusions** All the patients have been checked after 24 months with RMN, which has showed an optimal reconstruction of the cartilage lesion.

### Analysis of drugs effect on the osteoblastic activity in a murine model of bone fracture

C. Ruosi*[1], F. Querques[2], F. Granata[1], A. Barbato[1], S. Lupoli[1], G. Colella[1], L. Pastore[2]

[1]Dipartimento di Ortopedia e Traumatologia, Università "Federico II" (Neaples, IT);
[2]Dipartimento di Biochimica e Biotecnologie Mediche "Federico II" di Napoli e "CEINGE - Biotecnologie Avanzate" (Neaples, IT)

**Introduction** The aim of this study is to investigate the effects of new drugs on the osteoblastic activity in a murine model of bone fracture. For this purpose experimental femoral fractures are made in mice. With radiological and histological procedures will be evaluated the effect of losartan and D-serine administrated *per os*.

**Materials and methods** In order to discover the gene involved in osteoblastogenesis we have performed an in vitro high-throughput screening in a cell-based differentiation system. We have used a murine mesenchymal stem cells line that can differentiate into osteoblasts and produce mineralized depots in appropriate conditions. For this study we have considered two molecular target: the murine receptor of angiotensin Ib (corresponding to human ATR1) and a *N*-methyl-D-aspartate receptor (NMDA-R); these receptor seems to be involved in osteoblastic development. Angiotensin Ib receptor is blocked by losartan, a well known drug used in hypertension; on the NMDA receptor act the D-serine with co-agonize function, D-serine is the D-enantiomer of serine amino acid. For the murine model are used C57bL/6 mice. After anaesthesia a 1-cm right medial parapatellar incision is made. The longitudinal fibers of the quadriceps are divided and the patella is dislocated to expose the femoral condyles. A sterile 25-gauge needle is introduced into the intramedullary canal and advanced in the shaft of the femur to the level of the greater trochanter. The diaphysis is exposed. A snipper is used to perform a mid-diaphyseal fracture of the femur. The soft tissues are closed. The left femur is left unfractured. Animals are allowed free, unrestricted weight bearing after recovery from anaesthesia. A group of mice ($n = 10$) is treated with losartan, other group is treated with D-serine ($n = 10$); these drugs was effective in vitro. Animals are examined with DEXA scanning to evaluate the bone mineralization in the fracture site.

**Results** In the end the fractures are valuated with DEXA, micro-CT and, after the sacrifice, histological analysis.

**Discussion** We focus on the elucidation of genetic determinants of bone formation and, in particular, on the identification of genes involved in osteoblast differentiation. Such genes may increase our knowledge on the biological signals involved in this developmental process and constitute the basis for the development of drugs aimed at the increase of bone neosynthesis.

**Conclusions** The aim of this study is to obtain preclinical data of efficacy in order to evaluate the possible clinic use of this drugs, especially for the bone regeneration.

### *A08–BASIC SCIENCE*

### Intra-articular cytokine patterns in chronic meniscal tears associated with chondropathy

D. Gaddi*[1], M. Turati[1], C.A. Augusti[1], M. Gorla[1], M. Piatti[1], D. Munegato[1], A. Castelnuovo[1], E. Bresciani[2], L. Rizzi[2], A. Torsello[2], M. Bigoni[1]

[1]Orthopaedic Department, San Gerardo Hospital (Monza, IT);
[2]Department of Experimental Medicine, University of Milano-Bicocca (Monza, IT)

**Introduction** Articular injuries are related with arthritis. In 10–20 years from diagnosis almost 50 % of the patients with meniscal lesion have clinical signs of arthritis. Osteoarthritis is imputed to biochemical and biological factors as cytokines and metalloproteases activation. Studies describing anterior cruciate ligament injury cytokine patterns have shown the occurrence of pro-inflammatory patterns after a trauma which continued over time. Literature is lacking in studies that analyze analytically the synovial fluid cytokine's expression in patients with meniscal tear presenting or lacking chondral damage. The aim of the present study was to evaluate if chondral damage could condition biochemical parameters in chronic meniscal tears. Indeed we did analyzed concentrations of selected cytokines (IL-1$\beta$, IL-1ra, IL-6, IL-8, IL-10 and TNF-$\alpha$) in the synovial fluid of a human knees with meniscal tears comparing the grade of chondropathy.

**Materials and methods** The study includes thirty-eight patients with chronic meniscal tears (>3 months). Diagnosis was confirmed from a senior orthopaedic surgeon using present history, physical examination, magnetic resonance imaging (MRI) as well as confirmation by arthroscopic examination. The levels of interleukin (IL)-1β, IL-1ra, IL-6, IL-8, IL-10 and tumour necrosis factor (TNF)-α have been measured using ELISA test. Synovial samples were divided into meniscal tear without chondral damage graded from I to IV based on the Outerbridge classification: group A, no chondral damage; group B, damage ≤ II; group C, damage > II.
**Results** We have not observed statistically significant differences in cytokine expression of the three groups of patients for any cytokine. Our results show that the concentrations of IL-6, TNF-α, and IL-1β were positively correlated with those of IL-10. Moreover, the concentrations of IL-1ra were positively correlated with those of IL-8. In contrast, the concentration of IL-1ra were significantly negatively correlated with those of TNF-α.
**Discussion** The absence of statistical difference among patients with isolated meniscal tear compared to patients with chondral damage suggests that meniscal tear could be one of the factors that lead to a catabolic and pro-inflammatory state. Moreover the presence of correlations between different cytokines suggests the existence of a complex regulation of cytokines, which will require more specific and targeted investigations.
**Conclusions** We observed that cytokines patterns established in patients with isolated and chronic meniscal tear are important and comparable to those of patients with evident chondral damage of different degrees.

### Inter- and intra-observer reproducibility of the main scores used for the histological evaluation of cartilage repair

A.D.F. Massa*, A. Marmotti, A. Ferro, R. Rossi, P. Rossi, F. Castoldi, D.E. Bonasia

Clinica Ortopedica, Università degli Studi di Torino (Turin, IT)

**Introduction** Histological evaluation of cartilage tissue quality is fundamental in research on articular cartilage repair. It must be reliable, reproducible to allow a correct analysis of the various cartilage repair techniques. The literature reports several scoring systems, the main ones being: Mankin (HHGS), O'Driscoll, modified O'Driscoll, OsScore, the ICRS, ICRSII, Fortier, Sellers, Pineda and Wakitani. All of these evaluate the in vivo cartilage repair. The Mankin Score, created for the histopathological classification of osteoarthritic cartilage lesion severity, has also been used to evaluate cartilage repair. The Pineda and Wakitani scores are classified as simple, while the others as complex, according to the number of sub items. This study aimed at establishing the intra and inter-observer reproducibility of the 10 most commonly used scoring systems for the histological evaluation of cartilage repair. The authors tested the null hypothesis that the simple scores had higher reproducibility than complex ones. In addition, the experience of the investigators in cartilage histology was evaluated as a factor affecting final score.
**Materials and methods** Fifty biopsies (stained with Safranin O fast green) were taken from the femoral trochlea of New Zealand White rabbits where a large osteochondral defect was created and repaired with various techniques or left untreated. A wide variety of histological sections were examined blindly twice (with a 1-month interval) by 4 observers (2 experienced in cartilage histology and 2 inexperienced). The observers were given the same literature on the 10 scoring systems. Spearman's rho or Pearson's r were used to determine the intra-observer reproducibility (depending on the normality of the specimen), along with Cronbach's coefficient. The intra-class correlation coefficient (ICC) and Cronbach's coefficient were used to determine the inter-observer reproducibility.
**Results** The null hypothesis was rejected. The statistical analysis demonstrated a high intra-observer ($r = 0.99$-$\alpha = 0.99$/$\rho = 0.59$ -$\alpha = 0.71$) and inter-observer reproducibility ($ICC = 0.95$-$\alpha = 0.95$/ $ICC = 0.91$-$\alpha = 0.91$). Investigator's experience did not affect the reproducibility.
**Discussion** Despite this high reproducibility, the reliability of these scores cannot be evaluated due to the lack of a reference parameter for outcomes. Moreover, the scores evaluate different cartilage characteristics and are used with similar frequency in the current literature.
**Conclusions** In conclusion, even if the most commonly used and complete scores are preferable, all those examined in the present study can be used. However, the choice of the score should be based on the most important parameters for the specific study and on the project's design.

### Analysis of $Ca^{2+}$ flows induced by ATP in osteoblasts of osteoporotic and osteoarthritic patient in relation to their mineralization capacity

A. Cutarelli*[1], M. Celi[1], C. Frank[2], G. Fioravanti Cinci[1], U. Tarantino[1]

[1]Fondazione Policlinico Tor Vergata (Rome, IT);
[2]Rome, IT

**Introduction** The intracellular communication between the osteocytes by means of the generation of $Ca^{2+}$ waves seems to have a fundamental role in the processes of bone metabolism regulation, its mineralization and its remodelling. Two different ways of generating these waves are described: the activation of the P2Y or P2X purinergic receptors through ATP. The aim of this study is to evaluate the relationship between the activity of the purinergic receptors and the capacity of patients' cells to produce bone matrix.
**Materials and methods** The osteoblast cell cultures were isolated from fragments of bone trabeculae taken from 36 selected patients, who underwent total or partial hip prosthesis replacement, as a result of coxarthrosis or fracture of the femoral neck caused by osteoporosis, and who met the requirements set by the study itself (12 osteoporotic, 12 osteoarthritic and 12 control patients). To evaluate the $Ca^{2+}$ flows induced by ATP and subsequently by Thapsigargin fluorometric recordings were made with Fura-2AM. The quantification of the purinergic receptors, in particular of the P2X7 receptor, will be done using western blot. The mineralization capacity of the cells of the patient groups will be evaluated using Alizarin Red Assay.
**Results** The data show a statistically significant reduction in $Ca^{2+}$ flows induced by ATP in the cells of osteoporotic and osteoarthritic patients, compared to the control group. The administration of Thapsigargin following ATP stimulation resulted in increased $Ca^{2+}$ flows in the osteoporotic and osteoarthritic patients, compared to the control group.
**Discussion** The cellular communication between the osteoblasts and the osteoclasts is fundamental in bone resorption and neo-formation, and the ATP is one of the paracrine and autocrine signals implicated in these processes. The analysis of $Ca^{2+}$ flows induced by ATP is thus aimed at understanding how the expression and activity of purinergic receptors can influence various pathologies.
**Conclusions** The reduced $Ca^{2+}$ flows induced by ATP in the osteoporotic and osteoarthritic patients is attributable to a quantitative and qualitative alteration of the purinergic receptors. This hypothesis is supported by the results obtained after Thapsigargin stimulation, that show a reduced release of $Ca^{2+}$ in the control group, probably due to the increased activity of the P2X and P2Y receptors which favour, following the previous bond with ATP, the release of intracellular $Ca^{2+}$.

### Magnetic scaffolds fixed by permanent magnets for treatment of critical long bone defect

A. Russo*[1], S. Panseri[1], T. Shelyakova[1], M. Sandri[2], A. Ortolani[1], A. Visani[1], S. Meikle[3], J. Lacey[3], A. Tampieri[2], V. Dediu[4], M. Santin[3], M. Marcacci[5]

[1]Laboratorio NaBi, Istituto Ortopedico Rizzoli (Bologna, IT);
[2]ISTEC-CNR (Faenza, IT);
[3]University of Brighton (Brighton, UK);
[4]ISMN-CNR (Bologna, IT);
[5]Istituto Ortopedico Rizzoli (Bologna, IT)

**Introduction** Critical long bone defect represents a significant problem for orthopaedic surgeons. Bone is a complex tissue whose structure and function depend strictly on ultrastructural organization of its components: cells, organic (extracellular matrix, ECM) and inorganic components. The purpose of this study was to evaluate bone regeneration in a critical diaphyseal defect treated by implantation of a magnetic scaffold fixed by hybrid system (magnetic and mechanical), supplied through nanoparticle-magnetic (MNP) functionalized with vascular endothelial growth factor (VEGF) and magnetic-guiding.
**Materials and methods** A critical long bone defect was created in 8 sheep metatarsus diaphysis (20.0 mm in length; 6.00 mm inner diameter and 17.00 mm outer diameter), then we implanted a novel porous ceramic composite scaffold made of hydroxyapatite that incorporates magnetite (HA/Mgn 90/10), proximally fixated by two small cylindrical permanent parylene coated NdFeB magnets (one 6.00 mm diameter magnetic rod firmly incorporated into the scaffold and one 8.00 mm diameter magnetic rods fitted into proximal medullary canal, both 10.00 mm long); to give stability to the complex bone-scaffold-bone, screws and plate was used as a bridge. Scaffolds biocompatibility was previously assessed in vitro using human osteoblast-like cells. Magnetic forces through scaffold were calculated by finite element software (COMSOL Multiphysics, AC/DC Model). One week after surgery, magnetic nanoparticles functionalized with VEGF were injected at the mid portion of the scaffold using a cutaneous marker positioned during surgery as reference point in 4 sheep; other sheep were used as control group. After sixteen weeks, sheep were sacrificed to analyze metatarsi. Macroscopical, radiological and microCT examinations were performed.
**Results** Macroscopical examination shows bone tissue formation inside scaffold pores and with complete coverage of scaffolds, in particular at magnetized bone-scaffold interface. X-rays show a good integration of the scaffold with a good healing process of critical bone defect, and without scaffolds mobilization. These data were confirmed by the microCT that shown new formation of bone inside the scaffolds, in particular at magnetized bone-scaffold interface. Histomorphological evaluation confirmed a greater bone regeneration at magnetized interface, both in the group with VEGF-MNP that in the control group, than at not-magnetized interface. Comparing the two groups bone regeneration was greater when VEGF-MNP were injected.
**Conclusions** These preliminary results lead our research to exploiting magnetic forces to stimulate bone formation, as attested in both in vitro and in vivo models and to improve fixation at bone scaffold interface, as calculated by finite element software, and moreover to guide targeted drug delivery without functionalized magnetic nanoparticles dissemination in all body.

### Biochemical parameters changes after anterior cruciate ligament (ACL) injury

M. Bigoni*[1], M. Turati[1], C.A. Augusti[1], M. Piatti[1], F. Bernareggi[1], D. Munegato[1], E. Bresciani[2], L. Rizzi[2], A. Torsello[2], D. Gaddi[1]

[1]Orthopaedic Department, San Gerardo Hospital (Monza, IT);
[2]Department of Experimental Medicine, University of Milano-Bicocca (Milan, IT)

**Introduction** The natural history of an anterior cruciate ligament (ACL)-deficient knee has been and remains of large interest in literature. It has been demonstrated that an ACL rupture often leads to the development of degenerative osteoarthritis (OA). An ACL reconstruction that restore knee joint stability has not been shown to decrease the incidence of posttraumatic OA in this patient population. However, some reports indicate that 50–60 % of patients with ACL-reconstructed knees have radiographic evidence of OA after five years. It has been reported that a biochemical changes of the intra-articular synovial fluid happens in an ACL-deficient knee. We studied the biochemical characteristics of human knees with ACL rupture.
**Materials and methods** The sample population consisted of patients with isolated ACL tear ($N = 18$) and ACL tear associated with meniscal injury ($n = 30$) which were assigned to different groups according to the time elapsed between the injury and fluid collection: 22 acute (A), 7 early sub-acute (ESA), 11 late sub-acute (LSA) and 8 chronic (C). The samples were analysed for interleukin (IL)-1β, tumour necrosis factor (TNF)-α, IL-1ra, IL-6, IL-8 and IL-10 using commercially available sandwich enzyme-linked immune-adsorbent assay (ELISA test).
**Results** In the A group there were high levels of IL-1β, IL-6 and IL-8, whereas those of IL-1ra and TNF-α were significantly lower than the reported normal levels. IL-8 concentrations returned to normal already in the ESA group, whereas IL-6 decreased only beginning from the LSA samples. In chronic stage we found increased level of pro-inflammatory cytokines (TNF-α) and a decreased level of modulator mediators (IL-10 and IL-1ra). The concentrations of IL-8 were positively correlated to those of IL-10 and IL-1ra.
**Discussion** Our data show increased level of IL-6 and IL-8 in the acute phase of inflammation which could be responsible for triggering cartilage catabolism.
**Conclusions** In the chronic phase an over expression of pro-inflammatory cytokines and a low expression of IL-10 and IL-1ra could drive to OA trough a biochemical degenerative process of articular cartilage.

### Type 2 diabetes and markers of bone metabolism: interactions in fragility fractures healing

M. Celi*, F.M. Liuni, G. Giganti, U. Tarantino

Università degli Studi di Roma Tor Vergata (Rome, IT)

**Introduction** The inflammatory state associated with type 2 diabetes leads to alterations of bone tissue reflected both in terms of increased fracture risk and impairment of the reparative process. Among bone metabolic markers, osteocalcin has shown several effects on energy metabolism and insulin regulation, also suggesting a connection between bone and adipose tissue. Previous studies have shown an inverse correlation between osteocalcin and diabetes. In this study, we investigated the correlation between type 2 diabetes, osteocalcin and other markers of bone metabolism in women with fragility fracture.
**Materials and methods** One hundred and twenty women over 55 surgically treated for fragility fracture were included and divided into two groups according to the diagnosis of type II diabetes. In addition to blood glucose values were evaluated and correlated the serum levels of: osteocalcin, vitamin D, PTH, TNF-α, IL-6, calcium, phosphorus, bone alkaline phosphatase and osteoprotegerin.

**Results** Osteocalcin values were higher in non-diabetic controls ($p < 0.005$). In contrast, TNF-α and IL-6 showed higher values in the group of diabetic patients ($p < 0.005$).

**Discussion** The confirmation of an inverse correlation between serum osteocalcin and diagnosis of diabetes mellitus highlights a hypothetical protective role of this molecule for this condition. It's also possible that the disease itself causes a reduction of bone quality by a reduced concentration of osteocalcin. It was also observed a more evident inflammatory response, mediated by TNF-α and IL-6 in the diabetic fractured patient, probably related to a pre-existing state of chronic inflammation and linked to the diabetes itself. It's already proved an increased inflammatory state in diabetic patients that results in an alteration of the fracture repair process.

**Conclusions** The study of the correlation between type 2 diabetes and inflammatory markers and bone metabolism in patients with fragility fracture, can highlight the complex mechanisms that can lead to impaired bone quality and compromised fracture repair process in diabetic patients. It's therefore necessary to pay more attention to the resolution of the inflammatory state in the management of the diabetic fractured patient.

### *A09–SHOULDER AND ELBOW*

### Sport come back after shoulder instability surgery: Latarjet versus arthroscopic Bankart. A multi-centric study

D. Blonna[1], U. Cottino*[1], D.E. Bonasia[2], F. Pasquero[1], U. Mariotti[2], A. Marmotti[1], R. Rossi[1], F. Castoldi[1]

[1]SCDU Ortopedia e Traumatologia, Ospedale Mauriziano Umberto I, Largo Turati 62 (Turin, IT);
[2]Dipartimento di Ortopedia e Traumatologia, Città della Salute e della Scienza, Presidio Ospedaliero CTO-M. Adelaide, Via Zuretti 29 (Turin, IT)

**Introduction** Shoulder instability is a frequent pathology, in particular among sportsman. When conservative treatment fails the surgical approach may be useful. The goal of this study is to compare return to sport after arthroscopic Bankart and open Latarjet.

**Materials and methods** We conducted a polycentric retrospective study in two different university centres with two different surgical philosophies. One centre more frequently utilizes the arthroscopic approach, the other one the open approach. We included patients from 2004 to 2010. They were evaluated using upper limb TEGNER score, SPORTS score, Oxford score for instability and Simple Shoulder Value. Patients were coupled using such parameters: type of sport, age, sex, number of anterior luxations, instability type (TUBS *vs* AMBRI). Primary outcomes were: number of recurrences, return to sport, objective and subjective scores performed at the time of follow-up. Outcomes have been evaluated with the Students' $t$ test for parametric values and Fisher test for frequencies comparison. A logistic regression analysis has been executed to point out significative factors for sport come back.

**Results** Ninety-eight patients have been recruited; 74 presented similar characteristics according to comparison criteria and formed the case–control population (37 for each group). Basic demographic characteristics of the two groups resulted similar after the matching, confirming coupling validity. Multiple regression analysis pointed out that pre-surgical aspects that statistically influenced sport come back were: dislocations number, Tegner score and surgical procedure adopted. Contact sport, dominance, age and sex did not influence sport return. Average Sport score was 6.94 for Latarjet group and 8.35 in the arthroscopic Bankart one ($p = 0.004$). Oxford score was not different (40 *vs.* 42, $p = 0.2$), on the contrary, Simple Shoulder Value resulted different (92 % Bankart *vs.* 74 % Latarjet, $p = 0.008$). Arthroscopic Bankart group had a recurrence rate near significance level (10 *vs.* 0 %, $p = 0.05$).

**Discussion** Analysed data shows that Latarjet procedure has a lower recurrence rate, even if not statistically significative. However, sport come back is better in patients subject to arthroscopic Bankart.

**Conclusions** Relapsing shoulder instability treatment can be performed according to open Latarjet procedure or the arthroscopic Bankart approach. Patients have to be informed that recurrence is higher for the arthroscopic approach. However perceived result and sport come back are better in case of arthroscopic surgery.

### Post-operative rehabilitation after repair of the rotator cuff: supervised rehabilitation could provide better results?

F. Franceschi[1], R. Papalia[1], A. Palumbo[1], A. Tecame[1], B. Zampogna*[1], N. Maffulli[2], V. Denaro[1]

[1]Campus Bio-Medico (Rome, IT);
[2]Centre for Sports and Exercise Medicine, Barts and The London School of Medicine and Dentistry, Mile End Hospital (London UK)

**Introduction** Arthroscopic repair of the rotator cuff (RC) became a routinary surgery and an increasing number of patients asks for home rehabilitation programs. Therefore the new challenge is to find a balance between mobilization to prevent shoulder stiffness and rest to prevent re-tear and excessive shoulder pain. A supervised rehabilitation, thanks to the presence of a physiotherapist, guaranties for the balance. The risk arises when the patient carries on a home-based rehabilitation. Will he be able to observe the correct balance? In this randomized prospective study, we compared the supervised versus the home-based rehabilitation in terms of shoulder pain and shoulder stiffness rate.

**Materials and methods** Sixty patients affect by large RC tear (3–5 cm) have been enrolled and divided into two groups (30 patients per group): the SV group which carried on a supervised rehabilitation and the HM group, which carried on a home-based one. Patients from both groups were homogeneous for age, gender and activity level; moreover all the patients underwent the same surgical technique (single row arthroscopic repair). The rehabilitation program was based on the one described by Koo and Burkhart in 2010. Patients from HM group received a detailed protocol with pictures for each exercise. Outcomes have been measured by visual analogue scale (VAS) and ROM measures. Stiffness has been defined as described by Burkhart in 2011, as patients' dissatisfaction of their ROM.

**Results** After the first post-operative month, patients from SV group demonstrated a lower pain degree and a lower incidence of shoulder stiffness compared to HM group ($p < 0.005$). A similar trend was seen after the second post-operative month. On the contrary, after the third post-operative month, there were no statistically significant differences between the two groups.

**Discussion** Our results demonstrated as supervised rehabilitation programs provide a better control of the balance between rest and mobilization, lowering the rate of shoulder stiffness and reducing pain during the first three post-operative months.

**Conclusions** In our opinion, the best balance between shoulder mobilization and rest is insured by the supervised rehabilitation.

### *A10–FOOT AND ANKLE*

### The percutaneous stripping for chronic Achilles tendinopathy treatment in sports

B. Magnan*[1], M. Bondi[2], E. Samaila[1], T. Maluta[1], C. Dall'Oca[1]

[1]Azienda Ospedaliera Universitaria Integrata Verona, Ortopedia e Traumatologia DU (Verona, IT);
[2]Dipartimento di Ortopedia e Traumatologia, AO Carlo Poma (Mantova, IT)

**Introduction** Historically the treatment of chronic Achilles tendinopathy has focused on the degeneration areas within the tendon; new therapeutic options are inspired by the substantial literature evidence, the neovascularization presence outside the tendon, in the peritenon ventral region, setting the goal to stop the neo-formed vessels and nerves that have been shown to accompany them. Among these, the minimally invasive surgical technique of "stripping" has been recently introduced for the treatment of chronic Achilles tendinopathy: purpose of this research is the analysis of the rationale appropriateness on which this technique is based and, secondly, the preliminary results evaluation of its application in chronic Achilles tendinopathy treatment.
**Materials and methods** Between October 2009 and September 2011, 12 patients underwent percutaneous stripping of Achilles tendon; 3 out of 12 patients were treated bilaterally, for a total of 15 procedures. In two patients a resection of heel Haglund deformity was combined. Patients were prospectively clinically evaluated pre- and post-operatively, at 3, 6 and 12 months and at final follow-up, using the VISA-A SCORE (The Victorian Institute of Sports Assessment-Achilles questionnaire), and the AOFAS SCORE (The American Orthopaedic Foot and Ankle Society Outcome Score); MRI was used for a comparison between the imaging at the same times.
**Results** The mean follow-up was 19.27 months. The mean AOFAS score varied from 59.33 to 87.07 at final follow-up and VISA-A score varied from 43.07 to 81.93 at final follow-up. Both the average scores obtained with VISA-A and AOFAS scores are classified as very good (scores above 80). The analysis of the scores at final follow up shows that the best results were found in 53.3 % of cases with the VISA-A score and 86.7 % with the AOFAS score. The percentages relate to poor scores (scores below 60) were 6.7 % for VISA-A score and 6.6 % for AOFAS score.
**Discussion** The pain resolution and an early return to sport activities represent the surgical therapy success for tendinopathy overload, especially for athletes.
**Conclusions** The Achilles tendon stripping is to be considered as a treatment that combines the strength of evidence of its rational, with the advantages of a minimally invasive surgery; preliminary results support the efficacy of the stripping technique in the percutaneous treatment of Achilles chronic tendinopathy.

### Treatment of arthritis of the ankle using fresh osteochondral allograft

M. Cavallo*, G. Pagliazzi, F. Castagnini, A. Mazzotti, D. Luciani, R.E. Buda, S. Giannini

Isituto Ortopedico Rizzoli (Bologna, IT)

**Introduction** Severe post-traumatic arthritis of the ankle poses a reconstructive challenge in the young and active patients. Surgical treatment typically relies on arthrodesis or prosthetic arthroplasty. Reliable procedures despite many drawbacks. A biologic method of repair for high grades ankle arthritis may be fresh osteochondral allografts. The purpose of this study is to report clinical results of a series of 57 patients treated with bipolar fresh total osteochondral allograft (BFTOA) transplantation.
**Materials and methods** Fifty-seven patients (mean age $32 \pm 11$ years) affected by post traumatic arthritis of the unilateral ankle joint grade III received BFTOA of the ankle. The ideal patient to allograft match was permitted through CT scan and X-rays. Patients evaluation was carried out clinically by AOFAS and radiographically by X-rays, CT scans and MRI. In order to limit the degenerative changes that are frequently observed in the transplanted tissue, 20 patients were advised to soft immunosuppressive therapy for 6 months after transplantation.
**Results** The mean preoperative AOFAS score was $29.5 \pm 10.9$. At the 12-months follow-up, the mean AOFAS score was $76.5 \pm 12$, after 24 months the mean score was $74.1 \pm 13.3$, while at final follow-up of $47.3 \pm 16.7$ months the AOFAS score was $72.5 \pm 13.3$. One medial malleolar fracture and two tibial graft malpositioning occurred as intra-operative complications. Good radiographic consolidation was evident at 5 months of follow-up in all the cases. Excellent and good results were achieved in 48 patients. Among the nine failures, one graft was revised and eight grafts were converted to arthrodesis. Bioptic specimens of the transplanted tissue have been performed in all the patients at one year after transplantation, showing cartilage viability in more than 90 %. The 20 patients treated with immunosuppressive therapy experienced an earlier functional recovery associated with better radiographic results at 12 months of follow-up ($p = 0.041$).
**Discussion** The rationale at the base of allograft transplantation is to implant a viable osteochondral segment, capable to survive the transplantation and to be fully integrated by the host. However, despite the encouraging results, the technically demanding procedure and the high failure rate still remain cause of concerns.
**Conclusions** Although the encouraging results, BFTOA used for total joint replacement is still experimental and might be considered as a salvage procedure in otherwise not solvable situations. A proper selection of the patients is therefore a key point.

### Tightrope for the treatment of ankle syndesmosis injuries: a multicentric study

V. Tempesta*[1], M. Feola[1], E. Gasbarra[1], M. Arduini[1], R. Iundusi[1], V. Tempesta[2], U. Tarantino[1]

[1]Università Tor Vergata (Rome, IT);
[2]Ospedale Card. G. Panico (Tricase, IT)

**Introduction** TightRope is a new technique for the treatment of the injury of the ankle syndesmosis. TightRope is a non-absorbable suture between two metal cortical buttons, implanted across the syndesmosis. A previous technique for the treatment of this disease is represented by trans-syndesmotic screws. Problems derived by trans-syndesmotic screws are represented by an excessive rigidity of the ankle, by the need of the removal, by the loosening and the breakage of the implant. TightRope shows optimal functional outcomes, it's well-tolerated and it doesn't need to be removed. It can be used in association with a fibular plate, introducing it through the holes of the plate, but also in isolated ankle diastasis.
**Materials and methods** We analyzed 26 patients treated for injuries of the ankle syndesmosis with TightRope from October 2008 to December 2012. The ankle fractures were classified using the AO Danis-Weber Classification. We performed a clinical follow-up with the Foot and Ankle Disability Score (FADI) and with plan X-ray at 1 month, 3 months, 6 months and one year.
**Results** Sixteen patients had a type B fracture, 7 a type C fracture, 3 patients had an isolated injury of the ankle syndesmosis without fracture. Most of patients had good functional outcomes (15 had a FADI score of 100 %), even if some patients showed moderate difficulty in coming up on their toes and going down stairs (6 had a FADI between 85 and 100 %). In three cases we removed TightRope: in two patients because we decided to remove the fibular plate, in only one with a type C fracture associated with an injury of the ankle syndesmosis we decided to remove the system because the patient referred a sense of constriction at the ankle, even if after the removal there wasn't an improvement of the symptoms, hence, they were not caused by TightRope, but a consequence of the injury.
**Discussion** In clinical studies present in literature there are few cases of complications of soft tissues, but in most number of patients are not present symptoms or complications directly linked to the technique. There are still few clinical studies to have a definitive evaluation of this system.
**Conclusions** TightRope represents a safe, simple to use and reliable technique. Our patients had no complications caused by this system. There are only few clinical studies and a limit of our study is represented by the little number of patients included.

### Articular reconstruction of the subtalar joint in calcaneus fractures with an original minimally invasive osteosynthesis

A. Battaglia*, M. Tangari, P. Catania

Ospedale San Giovanni Addolorata (Rome, IT)

**Introduction** In articular displaced fractures of the calcaneus, open reduction and internal fixation (ORIF) allows to obtain anatomic reduction with good functional results, but it is not always indicated, with delayed surgery time; furthermore, it is often associated to complications as infections, skin wound dehiscence and thrombophlebitis, with an high amount of costs. On the other side, minimally invasive osteosynthesis (MIO) has such advantages as un-delayed surgery time, even in emergency cases, making it possible to avoid compartmental syndrome with less edema, pain, infections and a much faster functional recovery.
**Materials and methods** Kirshner wires, external fixators, screws and small plates are usually used for minimally invasive osteosynthesis. From 2009, in our department, we are using successfully the MIROS System (minimally invasive reduction and osteosynthesis system). It consists of modified elastic steel wires (respect to traditional pins) that are clamped in an high resistant metallic device which allows to obtain fracture fragment stabilization according to calcaneus trabecular bone system. Accordingly, the thalamus may be elevated without the need to be preserved with a cast, with early mobilization of the involved limb.
**Results** From 2009, at a short follow-up, 25 patients had good functional and radiographic results, with no complications, even comparable to those obtained with ORIF.
**Conclusions** MIO and particularly MIROS allow to obtain, in displaced calcaneus fractures, the stabilization of the thalamus, respecting its trabecular organization. This is possible thanks to the elasticity of the wires and the complete modularity and adjustability of the system. This technique has demonstrated to be a very feasible option for the treatment of these challenging fractures.

### *A11–SPINE*

### Progression of vertebral compression fractures in osteoporotic patients

C. Corradini, S. Pasqualotto*, F. Boisio, D. Tradati, C. Verdoia

Clinica Ortopedica, Università degli Studi di Milano (Milan, IT)

**Introduction** Vertebral compression fractures (VCF) represent, among fragility fractures, the most common type with an unpredictable progression. Objective of the study was to verify, through the use of serial X-rays, analyzed with a computerized morphometry, the progression of VCF, in a group of osteoporotic patients. Subsequently it has been considered if the progression's entity was conditional on the site and the type of fracture.
**Materials and methods** Fifty-three osteoporotic patients between 54 and 87 years old with clinical and radiological diagnosis of acute VCF have been followed for a two-year period. The entity of the deformity was detected at 1, 2, 4, 6, 12 and 24 months of follow-up and the data obtained with MorphoXpress were compared using a statistical analysis.
**Results** It has been observed a progression of vertebral deformities especially in the first 4 months from the acute event within a variable range from 2 to 30 %. It has not been found a substantial link between the entity of the progression and the initial entity of the deformities and the type of fracture; whereas the site of the fracture influences the progression. The dorso-lumbar tract has shown both a higher grade of deformation in Genant's classification at the moment of the diagnosis as well as a more important progression in the course of time compared to dorsal or lumbar fractures. At 6, 12 and 24 months it has been revealed an additional progression of the deformity, although lower and inconstant.
**Discussion** These data show how a first grade VCF could, in a quite short period, progress, reaching the most serious grade of Genant's classification. This could led the orthopaedic in reconsidering some conservative treatments in a surgical way, in particular within the first 4–6 weeks from the acute event, both to reduce the pain as well as to avoid the gradual deformation of the vertebral body. In the light of the data gathered, it appears fundamental the need of an early follow-up, by examination in 2–3 weeks in order to monitor strictly a possible progression of VCF.
**Conclusions** The clinical relevance of the study lie in the demonstration that VCFs are inclined to progress beyond the conventional healing period of 3 months. Therefore the clinical and radiological evaluations, with the use of morphometric analysis, should be closer in order to monitor the progression of VCFs and to reconsider a change in therapeutic strategies even in a surgical way.

### The use of mini-invasive techniques in oncologic spine surgery

R. Ghermandi*, S. Colangeli, A. Gasbarrini, S. Bandiera, G. Barbanti Bròdano, S. Terzi, M. Girolami, S. Boriani

Istituto Ortopedico Rizzoli (Bologna, IT)

**Introduction** Mini-invasive surgical procedures are quite common in spine surgery nowadays. The use of these techniques could be extended to the treatment of spinal tumours in selected cases but, if indicated, must respect the Enneking and Campanacci oncological criteria. Aim of this study is to evaluate the results obtained using mini-invasive techniques for the treatment of tumours of the spine (primary or metastases) in a series of patients selected over 1500 cases.
**Materials and methods** In selected cases of benign primary tumour the use of mini-invasive techniques could be considered as curative with a reduction of surgical aggressiveness. In 7 cases of spine osteoid osteoma the intralesional excision was performed using video assisted technique (in 6 cases videoendoscopic procedure by posterior approach and in 1 case thoracoscopic technique). Even in case of spinal malignant tumour (primary or metastases) the use of mini-invasive technique could represent an alternative to traditional surgery with a lower rate of complications and morbidity. It could be also indicated if surgical treatment is required after local radiotherapy In 12 cases of myeloma percutaneous screw fixation was performed. In 15 cases of pathological fractures (metastases) vertebroplasty only was performed. In 7 cases of metastases of the spine palliative surgery was performed using mini-invasive video-endoscopic assisted debulking and percutaneous screws fixation. In 8 cases of spinal metastases percutaneous transpedicle approach was used to treat the lesion (3 cases of melanoma metastases treatment by electrochemotherapy and 5 cases of liver metastases treated by radiofrequency).

**Results** Mean follow-up was 2 years (1–4). In case of osteoid osteoma no local recurrence were observed. In case of myeloma or metastases the use of these techniques allows good pain relief and a rapid recovery after surgery particularly important if post-operative chemotherapy or local radiotherapy were needed.

**Discussion** Mini-invasive surgical procedure could be considered as an alternative to traditional spinal surgery.

**Conclusions** In case of spine tumour (primary or metastases) patient selection must be carefully performed and the treatment, even if mini-invasive, must absolutely respect the oncological criteria.

### *A12–INFECTIONS*

### Infection risk factors in the surgical treatment of the proximal third humeral fractures: a multicenter study

D. Blonna*[1], A. Massè[2], B. Battiston[3], A. Tellini[4], E. Bellato[1], S. Marenco[4], L. Mattei[1], N. Barbasetti del Prun[1], F. Fissore[1], F. Castoldi[1]

[1]Divisione Universitaria di Ortopedia e Traumatologia, Università di Torino, AO Mauriziano “Umberto I” (Turin, IT);
[2]SCDU di Ortopedia e Traumatologia, Università di Torino, AO S.Luigi Gonzaga (Orbassano, IT);
[3]Struttura Complessa a Direzione Ospedaliera afferente al Dipartimento di Ortopedia, Ortopedia e Traumatologia ad Indirizzo Traumatologia Muscolo Scheletrica, Città della Salute e della Scienza, AO CTO Maria Adelaide (Turin, IT);
[4]Prima Clinica Ortopedica, Università di Torino, Città della Salute e della Scienza, AO CTO Maria Adelaide (Turin, IT)

**Introduction** The rate and the risk factors for acute infection after surgery for proximal humeral fractures is not known with certainty. This is a multicenter clinical study that was done with the aim of measuring the incidence and risk factors for acute infection.

**Materials and methods** We report a retrospective multicenter study including 452 proximal humeral fractures. The delay of surgery, type and length of surgery, age, sex, type of skin preparation, antibiotic prophylaxis, concomitant fractures and co-morbidities were the factors considered to be potentially related to infections. The data were collected in three university hospital centres from 2004 to 2011. Univariate analysis was performed by measuring the odds ratio for binary variables and linear regression for continuous variables. Possible confounding factors were controlled by logistic regression analysis.

**Results** The average age was 62.1 years (range 14–94), 314 were female. Eighteen patients (4 %) had a deep infection. The factors that correlated with infection were length of surgery (ODDS 1.009, $p = 0.05$), pre-operative lavage with chlorhexidine gluconate (ODDS 0.13, $p = 0.008$) and antibiotic prophylaxis (ODDS 10.73, $p = 0.03$). A post hoc analysis confirmed that the delay of surgery potentially increased the rate of infection (ODDS ratio 1.71, $p = 0.06$). The lowest incidence of infection was observed when the patients underwent surgery within 48 h of trauma.

**Discussion** The knowledge of risk factors for acute infections is of great importance, and is the basis for developing prevention strategies.

**Conclusions** This study suggests that washing the shoulder with chlorhexidine gluconate and avoiding the use of first generation cephalosporin in favour of more effective prophylactic therapy are effective at reducing the risk for infection after surgical treatment for proximal humeral fractures. With the data available we cannot recommend operating within 48 h from trauma although some evidence exists in this direction.

### Revision of one hundred and twenty infected tibial pseudoarthrosis with loss of bone and soft tissue treated with epidermato-fascial osteoplasty according to Umiarov

R. Mora*, L. Pedrotti, B. Bertani, G. Tuvo, S. Lucanto

Clinica Ortopedica, Polo Universitario Città di Pavia, Università di Pavia, (Pavia, IT)

**Introduction** The aims of the management of these lesions are infection healing, soft tissue reconstruction, bone reconstruction and consolidation with preservation of the limb length. Reconstruction can be achieved with plastic surgery techniques and by means of bone grafts; however these methods present many disadvantages and risks. Compression-distraction techniques, in the form of the so-called bone transport or bifocal compression-distraction osteosynthesis, are an interesting alternative. Epidermato-fascial osteoplasty, first described by Umiarov in 1982, is a particular type of bone transport, which offers the advantages of removing infection, accurately classifying the phases of simultaneous bone and soft tissue regeneration and eliminating large tissue losses without previous closure of soft tissues or use of grafts, because the transported fragment takes fascia and skin along during the transport, and gradually closes the edges of the soft tissue gap.

**Materials and methods** One hundred and twenty patients were treated between 1986 and 2010, with a follow-up of 2–26 years. Average age was 34 years (range, 21–57 years). Time elapsed from trauma to surgery was between 5 and 30 months. 59 patients had previously 2 operations, 45 had 3 operations, 16 had 4 operations. Cultures were positive for *Staphylococcus* in all cases, and also for *Pseudomonas* in 38 cases. In all cases an Ilizarov device was applied, with oblique wire transport technique. The resection was from 6 to 18 cm (average: 12 cm). An adequate antibiotic therapy, based on the culture results, was performed.

**Results** One patient died 40 days after operation due to pulmonary embolism. In 119 patients treatment lasted from 7 to 18 months. Sixteen patients developed a superficial infection at 1 or 2 wire sites, in 24 cases breakage of 1 or 2 wires was observed. The overall results were divided into bone and functional results according to the Paley classification: in all cases healing of infection, bone reconstruction, consolidation at the docking site (in 9 cases with the aid of an autoplastic bone graft), and soft tissue reconstruction were observed. Functional results were very good.

**Discussion** The radical removal of the necrotic and infected parts of both bone and soft tissues is the most important element for the success of treatment, by compression-distraction techniques, in severe tibial infected nonunions.

**Conclusions** Our results demonstrate that the Umiarov technique can provide excellent results in the management of these difficult cases.

### Treatment of infected nonunions of the tibia: assessment of different surgical techniques

F. Centofanti*[1], A. Camardo[1], A. Cambria[2], L. Fisichella[2], D. Fenga[2], M.A. Rosa[2]

[1]Istituto "Codivilla Putti" GIOMI SpA (Cortina d'Ampezzo, IT);
[2]Scuola di Specializzazione in Ortopedia e Traumatologia, Dipartimento di Scienze Biomediche e delle Immagini Morfologiche e Funzionali, Sezione di Ortopedia e Traumatologia, Università degli Studi di Messina (Messina, IT)

**Introduction** Infected nonunion is not a rare occurrence and it is extremely serious. In fact, it is a problem for the patient, the physician and society due to long treatment times and for frequent bad outcomes. The authors, following a review of clinical cases, are intended to provide guidance on the choice of the surgical technique to be used.

**Materials and methods** In the study were evaluated 347 patients with nonunion of the tibia, observed between 2002 and 2011, comparing the different surgical techniques employed. External fixation with Ilizarov apparatus, with its different possibilities of use (195 cases); locked intramedullary nailing with locked nail (58 cases) or expansion nail (70 cases). Other methods have been used as first choice or in association with the previous: fibula osteotomy, retrograde nail, axial external fixation, fibula-pro-tibia and k-wire fixation.

**Results** At a mean follow-up of 15 months and a stabilization period of one year we achieved the following results. Patients treated with Ilizarov apparatus: excellent results: 62; good: 84; poor: 27; bad: 22. Forty-three cases out of 58 treated with locked nail have not needed further treatments; patients treated with expansion nailing: 62 stabilization of 70. Fibula osteotomy has led to good results in 6 on 8 cases performed as a single treatment.

**Discussion** Ilizarov apparatus was the more versatile technique that has allowed us to address many of the problems posed by this disease, thanks to its various mounting options. Counter is not free of complications. The blocked nailing technique is a method widely tested and with a good adaptability, but has more limited directions and must be applied after one or more times preparatory debridement.

**Conclusions** In treatment of infected nonunions of the tibia both external fixation techniques and the internal fixation ones can be used with satisfactory results to address the challenges and diversity of this serious disease. Eutrophic and oligotrophic nonunions, which occur closer to the epiphysis, with large bone loss and with active septic focus, found the gold-standard in Ilizarov apparatus: very adaptable technique, minimally invasive to soft tissues and bone; it allows to fill up large bone losses and allows an almost immediate full load.

### Ankle arthrodesis and leg lengthening in the tibial distal defect

A. Kirienko*[1], A. Peccati[1], E. Arnaldi[1], N.M.A. Portinaro[2]

[1]Istituto Clinico Humanitas (Rozzano, IT);
[2]Clinica Ortopedica, Università degli Studi di Milano (Milan, IT)

**Introduction** Septic pseudoarthrosis and distal leg osteomyelitis can require, in the most severe cases, the limb amputation, and the surgical treatment aims at eradicate the infection, making the whole limb more stable. Ankle arthrodesis is one of the treatment options, but it often leads to an iatrogenic leg shortening, which can be easily corrected with the Ilizarov method during the same treatment, using the same frame.

**Materials and methods** Between 2006 and 2011 we treated 13 patients (8 males and 5 female, meanly aged 42.17), affected by tibial bone defect (post-traumatic or a consequence of ankle prosthesis removal), osteomyelitis or septic pseudoarthrosis of the distal tibia, using the Ilizarov technique and the contemporary proximal leg lengthening. The mean pre-op AOFSAS score was 53.

**Results** All the patients underwent prosthesis removal (if present), resection of the distal pseudoarthrosis or osteomyelitis, proximal leg osteotomy and then proximal lengthening, bone transport and distal compression for ankle arthrodesis (single, double or triple arthrodesis). The mean lengthening was 5.5 cm. In 2 cases a new surgery was required (frame renewal and open cruentation of the pseudoarthrosis in the site of arthrodesis). After the frame removal all the patients used a plaster cast to walk, meanly worn for 4 weeks. No patients had pain after the plaster cast was removed. The clinical result has been judged by the patients themselves as excellent in 8 cases, good in 4 cases and poor in 1 case. The mean post-treatment AOFSAS score was 67; the best scores were registered when the subtalar joint was saved. The mean treatment time was 37 weeks, and the mean follow-up time has been 4.6 years.

**Discussion** Ankle arthrodesis with the Ilizarov external fixator presents many indications, and permits an easy correction of the iatrogenic leg shortening, beside the contemporary correction of any associated bony deformities.

**Conclusions** Ilizarov method can be considered as a limb-salvage procedure for severe cases when other surgical techniques for articular reconstruction or internal fixation failed, allowing the infection to be eradicated and the treated limb to be more stable.

### Effect of delayed surgery on bacteria colonization of the skin for proximal humeral fracture

D. Blonna[1], G. Banche[2], V. Trombetta*[1], A.M. Cuffini[2], V. Allizond[2], F. Castoldi[1]

[1]Reparto SCDU di Ortopedia e Traumatologia, A.O. Mauriziano Umberto I (Turin, IT);
[2]Dipartimento di Scienze della Sanità Pubblica e Pediatriche (Turin, IT)

**Introduction** Post-surgical infection is one of the most serious complication after surgery for proximal humeral fracture. It has been suggested that delay of surgery after proximal humeral fracture may increase the risk of developing an acute infection. The aim of this study was to figure out if the delay of surgery could increase the bacteria colonization of the skin.

**Materials and methods** A group of 25 patients with proximal humeral fracture have been recruited in orthopaedic emergency room. For each patient three skin culture swabs were executed in correspondence to the deltopectoral approach brushing on the skin surface respectively in the day of the fracture (day 0), the day after the fracture (day 1) and five days after the fracture (day 5). In the microbiological lab, with each skin swab, were developed bacteriological cultures on three different types of culture media: for staphylococci (MSA), aerobic bacteria (NA) and anaerobic bacteria (Schaedler). The data were compared using Student's *t* test.

**Results** In all the three types of culture media, an exponential increase of the bacterial charge was measured (cfu/ml). The *Staphylococcus aureus* increased from $4.5 \times 10^2$ (day 0) to $2.92 \times 10^3$ (day 1) to $8.0 \times 10^3$ cfu/ml (day 5) ($p < 0.05$). The *Propionibacterium acnes* increased from $7.68 \times 10^3$ (day 0) to $4.48 \times 10^3$ (day 1) to $1.46 \times 10^5$ cfu/ml (day 5) ($p < 0.05$). The coagulase-negative staphylococci increased from $7.27 \times 10^3$ (day 0) to $9.74 \times 10^4$ (day 1) to $4.80 \times 10^5$ cfu/ml (day 5) ($p < 0.05$).
**Discussion** The bacterial contamination on the patients skin for pathogenic bacteria before the surgery is an important variable to take in consideration for infections risk, in particular, the delay of surgery has proved to be a determining factor on the modify the bacterial charge on the skin.
**Conclusions** The delay of surgery increased exponentially the bacteria colonization of the skin in the deltopectoral approach including common pathogenic bacteria. This might justify the correlation between delay of surgery and risk of infection.

### *A13–PROSTHESES*

### High flex, motion-guided total knee arthroplasty: clinical and biomechanical mid-term follow-up

P. Antinolfi*[1], R. Caira[2], G. Placella[3], E. Sebastiani[3], G. Potalivo[4], G. Cerulli[5], A. Caraffa[3]

[1]Clinica Ortopedica e Traumatologica, Ospedale S. Maria della Misericordia (Perugia, IT);
[2]Universita degli Studi di Perugia (Perugia, IT);
[3]Clinica Ortopedica e Traumatologica, Università degli Studi di Perugia (Perugia, IT);
[4]Let People Move Research Institute, Nicola's Foundation ONLUS (Arezzo, IT);
[5]Nicola's Foundation ONLUS, Let People Move Research Institute, International Orthopaedic and Traumatologic Institute, Università degli Studi di Perugia (Perugia-Arezzo, IT)

**Introduction** The study of the design of the components of total knee arthroplasty is constantly evolving in the aim to obtain joint kinematics increasingly similar to the physiological one. Despite this, the kinematics of the arthroplasty represent an approximation of the actual recovery of the normal range of motion of the joint however allowing the recovery of a large part of the function of an otherwise painful joint. The first attempts to recreate normal knee kinematics, now dating back several years, were made using prosthetic designs called high-flex that have recently undergone further development to optimize the dynamic range.
**Materials and methods** In the Department of Orthopaedics and Traumatology at the Santa Maria della Misericordia Hospital in Perugia between 2009 and 2012, 109 high flexion, guided motion with anterior and posterior cam/post mechanism total knee prostheses (Journey BCS, S & N, Memphis, USA) were implanted. Eighty-five patients participated in a medium term follow-up. All had undergone prosthetic implant for primary knee osteoarthritis with varus axis, 46 women (55 %) and 38 men (45 %), mean age 67 years, mean BMI was 28.7.
**Results** In 3 cases (3.5 %) failure of the implant was reported requiring prosthetic revision: 2 were due to infection (at 5 and 6 months), the third for dislocation of the polyethylene liner at 27 months. The majority of patients were satisfied or very satisfied with the result, 90.6 % started doing most of the activities of daily life without pain, and 21 % of them said they had started physical activity or recreational sports. We evaluated 5 different scores in the post-operative period. Oxford Knee Score was mean of 34.51 that demonstrates a satisfactory result, while the KOOS: 74.64, WOMAC Knee Score: 25.3, Knee Society Score: 89.95 and Knee Society Functional Score: 84.81 were all in the range of excellent results.
**Discussion** The more recent prosthetic systems are supposed to reproduce joint mechanics and a function closer to normal anatomy by means of a high-flexion design which is accompanied by kinematics that reproduce better the differences between medial and lateral compartment and facilitate the kinematics of the patellofemoral joint.
**Conclusions** At medium follow-up the data collected were excellent in 90 % of the cases with a good level of return to normal daily activity providing promising results that must be validated by longer follow-ups in order to confirm any true benefits of these designs corroborated by objective functional data.

### Results in the revision of knee prosthesis for painful stiffness

F. Verde*, M. Massaro, M. Corbella, S. Romagnoli

IRCCS Istituto Ortopedico Galeazzi (Milan, IT)

**Introduction** The stiff knee can be caused by a flexion contracture or extension or by a contracture defined as mixed. We consider a stiff knee when the Range of Motion (ROM) is less than 60° after 6 months from total knee arthroplasty.
**Materials and methods** From 1988 to 2005 we performed 775 total knee arthroplasty revisions of which 168 unicompartmental prostheses. Out of 607 denture revisions, a medial approach was used in 56.5% of cases (lateral in 3.6%, a quadriceps snip in 7.2%, Y or V quadriceps plasty in 1.7%) while in 32% of cases an osteotomy of the tuberosity was performed.
**Results** There is a direct correlation between anterior knee pain and the level of the joint line according to the results of the revisions, while there is no direct correlation between pain and morphology of the patella. In our opinion, in cases of stiff knee with patella baja, the Y or V plasty of the quadriceps muscle may decrease the efficiency of the muscle. While the osteotomy of the tuberosity can increase the lever arm of the quadriceps muscle and therefore its efficiency.
**Discussion** It is important to avoid the tourniquet during these surgical procedure so as to allow a proper assessment of the length and flexibility of the quadriceps muscle and the extensor apparatus. The intensity and duration of physical therapy to reduce the deficit in active extension after proximalization of the tuberosity depends on the magnitude of the displacement of the tuberosity, the age of the patient and the duration of the deformity.
**Conclusions** In conclusion, the revision of a stiff total knee arthroplasty is always a difficult surgical procedure with a high failure rate given by the damage of the extensor mechanism and insufficient recovery of range of motion. These complications are not remediable with other surgical procedures. The tuberosity osteotomy is a good choice that does not require special surgical aids and the post-operative recovery does not change.

### Multicentre study on the learning curve in minimally invasive direct anterior approach for hip arthroplasty: the experience of the AMIAA Group

V. Alecci[1], M. Ceccarelli[2], C. Pagnuzzato[3], F. Pleitavino[4], P. Prati*[5], L. Romano[6], C. Trevisan[7], M. Trono[8], M. Valente[1]

[1]SOC Ortopedia e Traumatologia Ospedale San Polo, ASS 2 Isontina (Monfalcone, IT);
[2]Casa di Cura Santa Zita (Lucca, IT);
[3]Chirurgia Anca I, Istituto Ortopedico R. Galeazzi (Milan, IT);
[4]UOC Ortopedia e Traumatologia, AO Villa Scassi (Genoa, IT);
[5]UOC Ortopedia e Traumatologia Ospedale San Giovanni Bianco, AO Treviglio (San Giovanni Bianco, IT);
[6]UO Ortopedia e Traumatologia Ospedale di Tione-Madonna di Campiglio (Trento, IT);
[7]UOC Ortopedia e Traumatologia Ospedale di Lovere, AO Bolognini Seriate (Lovere, IT);
[8]UO Ortopedia e Traumatologia, Ospedale Infermi, AUSL Rimini (Rimini, IT)

**Introduction** Innovation is a crucial element in technical progress but the introduction of new methods must not have a negative impact on the final results. The direct anterior approach to the hip (DAA) has been credited for its low invasiveness with benefits in terms of reduced blood loss, pain and length of hospital stay but his learning curve has been considered challenging.
**Materials and methods** In this multicentre retrospective study, 6 orthopaedic centres in which DAA is used for hip replacement have provided data of their first 30 cases to assess the incidence of complications and their relevance on the clinical course, the positioning of the components and the functional result at follow-up.
**Results** In the 180 cases examined (109 females and 71 males, mean age 71.7 years, range 30–100) were implanted 156 arthroplasties and 24 endoprosthesis. 26 postoperative complications occurred in 25 patients (13.3 %). In 17 cases (9.4 %), the complication had at least one adverse result: in 10 cases (5.5 %) a lengthening of surgical time, in 8 cases (4.4 %) a lengthening of the time of recovery of the patient, in 6 cases (3.3 %) a lengthening of the time of weight bearing recovery, in 10 cases (5.5 %) a lengthening of the time of hospitalization. Only in 6 cases out of 180 (3.3 %) the complication led to a deterioration of the final outcome. No dislocation was reported. The positioning of the components was evaluated in a subgroup of 60 patients,: the average orientation of the femoral stem relative to the axis of the femoral was 0.9° ± 1.4° (range 0°–6°) and the cup inclination was 46° ± 7° (range 28°–64°). Three stems (5 %) showed a varus/valgus positioning >3° and 6 cups (10 %) had an inclination <35° or > 55°. In this subgroup function measured with the Harris hip score increased from 56 ± 20 pre-operatively to 89 ± 13 post-operatively. The average duration of surgery was 98 ± 28 min.
**Discussion** This study reports the learning curve of the minimally invasive DAA to the hip of six different centres throughout the country. Reported complications were few and in most of the cases graded as minor since they did not have any impact on the clinical course. Only in 3.3 % of cases there was a complication that negatively affected the final outcome.
**Conclusions** The introduction of the DAA has proved practicable with complication rates comparable to those recordable in the usual clinical practice. Innovation needs to be addressed through appropriate education program.

### ORAL COMMUNICATIONS

### *C28–HAND AND WRIST 1*

### Locking fixation for the treatment of distal radius fractures: evolution of the plates. Our experience

I. Cappelletti*, F. De Sessa, P. Lazzoni

Ospedale Evangelico Internazionale (Genoa, IT)

**Introduction** The surgical treatment of wrist fractures has two aims: the anatomic reduction of bone fragments and their stable fixation. The authors carried out a medium and long term revision on cases treated with different types of volar plates, which have developed significantly in the past few years, reporting the advantages of this mode of fixation and the problems encountered.
**Materials and methods** The study evaluated 234 distal radius fractures (classified according to AO guidelines) treated with volar fixation as per Henry's approach, with various types of plates. The aim is to observe the advantages and disadvantages of the different types of implants used, on the basis of the outcomes.
**Results** Two hundreds and two patients were followed-up retrospectively at a significant time using the DASH questionnaire, Modified Mayo Wrist Score, a wrist radiograph in the orthogonal projections, dividing the patients according to the fracture morphology according to the AO classification. All the fractures healed, regardless of the age and bone quality. The mean radiographic parameters at distance were 21.4 degrees of ulna angulation, 9.9 degrees of palmar tilt, 0.8 mm of negative ulna variance with average 1.6 mm articular step. The mean wrist arc of motion was 56 degrees of flexion, 62 degrees of extension, 80 degrees of pronation and 77 degrees of supination, with lower values in group C.
**Discussion** The plates features of low profile, shape, reduced pegs diameter, multidirectionality or pluridirectionality, tools simplicity are qualities that increasingly lead the general trend to the choice of operative intervention in order to meet the patients' high functional requirements.
**Conclusions** The volar plate proved reliable both in young patients and in elderly patients with osteoporosis. We believe that fixation through a double-sided plate for the support of the distal radius metaphysis can represent an evolution to obtain an increased stability of the radial styloid. The simplicity of the surgical tools and the choice of having the unidirectional or multidirectional choice for the pegs within the same plate guarantee higher versatility in a single fixation tool for different types of fractures and enable to obtain a better result, in shorter time. The decrease in surgical time also guarantees a reduced use of resources and a reduction of costs.

### The treatment of nonunion of the distal radius

S. Odella*, S. Scarponi, V. Pedrini, F. Torretta

Istituto Ortopedico Gaetano Pini (Milan, IT)

**Introduction** The nonunion of the distal radius occur only in 0.2 % of cases, the most frequent causes are to be found in the error in the processing or in the type of surgical technique choice or in its execution. The presence of an associated fracture of the ulna increases the instability and therefore the rate of nonunion.
**Materials and methods** At our UO surgery of the hand and wrist were treated from 2001 to 2011, 3500 fractures of the distal radius, we treated 5 cases of nonunion of the distal radius. In all cases the cause was to be found in a failure of the surgeon in the choice of treatment or in the execution of the surgical technique. The average age of patients is 48 years, with a women to men ratio of 3/2.
**Results** For the evaluation of functional results we used the DASH score and Mayo wrist score. We obtained 60 % of good results, bone healing, absence of pain, restoration of ROM and strength, 40 % of bad results: not reaching the bone healing, pain and functional limitation.
**Discussion** The nonunion of the distal radius is a rare complication, for which there is still considered the gold standard surgical treatment, the literature considers it necessary surgical treatment if, in addition to the non-healing of the fracture are associated with pain and misalignment of the fracture stumps.
**Conclusions** In the cases treated by us we have recognized as the main cause of the onset of nonunion of the distal radius mistake on the part of the surgeon in choosing the type of conservative treatment or surgery, or in the execution of the surgical technique itself. Trephination of the outbreak of nonunion, the autologous bone graft and the reduction and synthesis are stable elements that according to our experience can lead to healing.

### Different external fixation systems on distal radius fractures treatment

A. Palombi*[1], E. Gasbarra[2], R. Iundusi[2], U. Tarantino[2]

[1]Dottorato di Ricerca, Università degli Studi Tor Vergata (Rome, IT); [2]Università degli Studi Tor Vergata (Rome, IT)

**Introduction** Distal radius fracture are among the most frequent fracture lifetime. Their treatment is challenging, and, even if many kinds of fixation are accepted, there is not yet agreement on surgical management. Aim of this study is to evaluate and compare outcomes of different types of external fixation in treatment of distal radius fractures: bridging external fixators, non-bridging (dynamic) external fixators, elastic pinning, K-wires + cast.
**Materials and methods** Since September 2011 to May 2012, 89 patients with distal radius fracture underwent to surgical management. Seventy-six of them (50 women, 26 men) were recruited for a prospective study. They have been evaluated on radiographic, clinical and functional (objective and subjective) parameters at 2, 6, 12 weeks from surgery, and then at 6, 12, 24 months.
**Results** Major and minor complication were registered and classified as: superficial wound infection (9.2 %), reflex neurovascular dystrophy (5.3 %), minor neurological diseases (9.2 %), deep wound infections with secondary fixation failures (two patients). Final outcome was poor in 3 patients; all the other patient had good or excellent final outcome, and no complication were observed.
**Discussion** Slightly better short-term results were registered on functional scores (ROM and DASH) among external fixator patients (bridging and dynamic ef). K-wire + cast treatment showed to be the less comfortable system.
**Conclusions** Percutaneous fixation, such as elastic pinning or k-wires + cast immobilization, showed best results in simple, extra-articular fractures; external fixators are the best solutions in unstable articular fractures, wherein dynamic of seem to be preferable in non-comminuted displaced fractures. A careful evaluation of ROM during follow-up is on our opinion an important parameter, even if it is, unfortunately, operator-depending.

### Treatment with angular stability plates in the distal end of the radius complex fractures

A. Famoso*[1], G. Restuccia[1], L. Micciché[2], M.A. Rosa[2]

[1]UOC Ortopedia e Traumatologia “Garibaldi Centro” (Catania, IT); [2]Università degli Studi di Messina (Messina, IT)

**Introduction** Fractures of the distal end of the radius are among the most frequent skeletal injuries. They represent, according to international statistics, about one-sixth of the fractures treated in emergency departments and 75 % of forearm fractures. They occur more frequently in women with a incidence peak between 60 and 70 years. The objective of this study is to analyze the advantages and limitations of the technique ORIF for the treatment of fractures of the distal radius.
**Materials and methods** The authors examined a case series of 78 patients treated between 2010–2012, putting precise directions to internal fixation with only dorsal or palmtop plate, or to the summary with double dorsal and palmtop plate. In 65 patients was performed palmtop access, in seven a dorsal access and in six patients a double dorsal and palmtop access. Results were evaluated by analyzing the X-ray examination post-operatively and at a distance, the DASH questionnaire for the degree of patient satisfaction, the Mayo Wrist Score modified by Cooney for functionality, pain and range of motion.
**Results** The median follow-up is two years and half (4 to 1). All fractures healed with a mean healing time of about 5 weeks, there has been no evidence of necrosis in the articular fragments. The radial shortening was on average less than 1 mm (with a range of 0–2 mm), the loss of radial inclination was on average less than 5° (range 0°–4°). With the Mayo Wrist Scores were found 28 excellent results, 34 good, 10 discrete and 5 bad. The DASH score showed a high degree of satisfaction.
**Discussion** Although the considerable incidence of such injuries, there isn’t a uniformity of thought in the literature regarding their treatment. In the last two decades the use of the ORIF (open reduction internal fixation) technique is emerging thanks to the refinement of techniques and to the improvement of materials, such as to be now considered a tool of routine for the treatment of complex fractures wrist.
**Conclusions** The modern angular stability plates created and designed for the distal end radius fractures have greatly improved the functional results of the most complex lesions. The use of these plates has always allowed stable fixation, enabling early wrist mobilization and a more rapid return to daily activities of the patient.

### Osteosynthesis techniques’ comparison in fractures of distal epiphysis of radius: 60-month follow-up

A. Merenda*[1], G. Topa[2], S. Creaco[1], S. Marcellino[1], M.A. Rosa[1]

[1]Scuola di Specializzazione in Ortopedia e Traumatologia, Dipartimento di Scienze Biomediche e delle Immagini Morfologiche e Funzionali, Sezione di Ortopedia e Traumatologia, Università degli Studi di Messina (Messina, IT);
[2]Azienda Ospedaliera Bianchi Melacrino Morelli, “Ospedali Riuniti” (Reggio Calabria, IT)

**Introduction** Wrist’s fractures are one of the most frequent lesions observed by orthopaedist, also for the progressive increasing in the population average age (people over 65 are more than 20 % of general population). Management of these fractures is mainly surgical and with many options in treatment.
**Materials and methods** Authors performed a retrospective study on a series of 394 wrist fractures treated surgically between January 2007

and December 2012. One hundred and thirty-seven were males and 262 were females, with male/female ratio 1:2. All fractures were preoperative studied with X-ray in antero-posterior and latero-lateral, oblique projections and classified according with AO/ASIF criteria. Two hundreds and five cases (52 %) were classified as type A, 118 cases as type B and 70 as type C (18 %). Surgical techniques of osteosynthesis included: wires percutaneous synthesis, volar plate fixation, reduction and stabilization with external fixator. Patients were followed with clinical and radiological examinations. DASH and modified Mayo Wrist Score by Cooney were used to quantify post-operative functionality, pain and ROM.
**Results** Clinical and instrumental evidences were positive with good functional recovery and pain relief. Parameter results for the questionnaire DASH and the tab Mayo Wrist Score showed 88 % with satisfaction between excellent and good 12 % between fair and bad. Among complications we observed: vicious consolidation, stiffness with impairment of flexion–extension and supination, radio-carpal impingement.
**Discussion** The articular fracture of distal radius are very frequent and complex distribution of intra-articular fracture fragments makes reason to different types of osteosynthesis. A post-operative rehabilitation treatment protocol, customized for each patient, and type of surgery performed is required for a complete joint recovery.
**Conclusions** Fractures of distal radius should be considered as fractures with articular involvement. Our target is to aim an anatomical reduction. The osteosynthesis of articular surface can be performed using volar plate. The system with percutaneous wire is a viable alternative that allows the patient to perform normal daily life functions, related to an early functional recovery. The external fixator is a valid treatment especially in comminuted fractures with good results.

### New materials in hand surgery: the pyrocardan in TM osteoarthritis

P. Ghiggio*[1], R. Lombardo[1], M. Pettiti[1], L. Trifilio[1], F. Bergamin[2], G. Nobile[1]

[1]ASL To4 (Ivrea, IT);
[2]ASL To3 (Turin, IT)

**Introduction** Arthritis of the first carpometacarpal joint (CMC) is doubtless the most frequent and widespread disease which affects hand's joint. On the basis of clinical illness severity and X-ray imaging we can treat it with a conservative or surgical approach. Eaton's classification just resumes the illness with four clinical and X-ray parameters. Following Eaton's scale we can treat this disease in conservative way, generally in first stages. A surgical approach must be chosen in most advanced stages using either arthroplasty or tenoplastics in association with total or partial trapeziectomy. Some borderline early situations can be treated with the pyrocardan following correct indications.
**Materials and methods** The pyrocardan is a shaped spacer in pyrocarbon that allows a painless articular function restoration. In our opinion the correct indication is the Eaton's grade 2. The surgical approach is by a dorsal incision, a capsular rectangular flap is performed, the osteoarticular surfaces resection in economic and is followed by a cut adjustments in order to position the spacer in the correct way. Sometimes the APL can be used to effort the capsule to give it a satisfying strength. The implant is available in seven sizes. Ten patients were treated (seven females and three males) at Eaton's scale 2. A cast for three weeks is used in the post-operative care followed by an adequate physiotherapy.
**Results** The follow-up was in a range from six to twelve months. Results have been evaluated with La Caffinière method, considering pain (VAS scale), functional restoring and X-ray (TM ray axiality and implant position). Patients had a good degree of satisfaction and we are encouraged to go on with this method.
**Discussion** Pyrocardan is a valid implant when the surgical choice needs a very strong material, with high biocompatibility and isoelasticity with bone. Moreover the surgical procedure is not much invasive and, in case of poor results and complications, consents to apply other wider procedures such as ligaments or arthtroplasties and trapeziectomy.

### *C29–HAND AND WRIST 2*

### Nonunion of the carpal navicular: surgical treatment and PRP

A. Sanfilippo*[1], G. Letizia Mauro[2], G. Salomone[1], M. Sallì[2], A. D'Arienzo[1], V. Colombo[2], M. D'Arienzo[1]

[1]Clinica Ortopedica e Traumatologica, Università degli Studi (Palermo, IT);
[2]Cattedra di Medicina Fisica e Riabilitativa, Università degli Studi (Palermo, IT)

**Introduction** Nonunion is an important complication of carpal navicular fractures. These fractures lead to pseudarthrosis in a relatively high percentage of cases (5–15 % according to the literature) when the gap between the fragments is more than 1 mm. The aim of this study is to report the medium term results of a reconstruction technique with cannulated screw combined with platelet rich plasma (PRP) in Herbert fractures C (late consolidation), D1 (fibrous union without deformity) and D2 (pseudarthrosis with initial deformity).
**Materials and methods** Between April 2008 and May 2011, 15 patients (12 male and 3 female) with ages ranging from 30 to 52 years (average 41.6 years) were treated with the cannulated screw and platelet gel technique. In 3 patient the diagnosis was of late consolidation, in 9 cases of nonunion without deformity and in remaining 3 cases of nonunion with initial deformity. Before and after the treatment the patients were followed-up clinically (pain, wrist ROM and muscular strength) and radiologically.
**Results** Union was achieved in all the patients: in 12 cases we obtained excellent results with total resolution of pain, completely recovery of articular ROM and a 90 % return of normal muscle strength. In 2 cases the outcome was considered good for occasional pain and a moderate decrease in motility and muscle strength. Only in one case the result was bad persisting unchanged pain and functional impairment.
**Discussion** Our experience with the technique combined of osteosynthesis with cannulated screw with PRP, used as a filler and a stimulator of bioreparative and regenerative processes, showed advantages such as: simple instrumentation, easy technique, visual control of the times of reduction and synthesis, safety of PRP and its low cost.
**Conclusions** We consider that our good results in the treatment of the nonunions of the carpal navicular is ascribable, well as the choice of the type of osteosynthesis, to osteogenic capacity of PRP and to post-operative rehabilitation treatment both able to restore the wrist and hand functionality to normal daily activities.

### Replacement with APSI implant of scaphoid proximal pole: clinical results with follow-up at two years

M. Rosati*, A. Poggetti, G. Ciapini, L. Dolfi, M. Lisanti

Clinica Ortopedica Traumatologica (Pisa, IT)

**Introduction** Nonunion and necrosis of proximal pole of scaphoid are important challenges for the orthopaedic surgeon. Some authors recommend scaphoid replacement with: silicone, autologous tissue, cobalt-chromium alloys. Perquinot (2000) suggested prosthetic proximal pole scaphoid replacement with pyrocarbon APSI implant.

**Materials and methods** From 2008 to 2012, were treated 16 patients (2 SNAC I, 12 SNAC II, 2 SNAC III): all patients were male, average age 46 years, 10 heavy workers. In 65 % dominant hand involved. Patients were subjected to clinical evaluation (Krimmer's tests, study with Jamar) and X-rays in 2 projections before and after surgery. The APSI implant has applied with dorsal approach and capsular joint denervation. Very important for us steady reconnection of dorsal capsule, often with a mini bone anchor on the radius. In 4 cases it was necessary to associate a styloidectomy. The patients were evaluated with an average follow-up of 27.2 months with clinical evaluation, Jamar dynamometer test and X-rays.

**Results** We observed clinical improvement in all patients (VAS: from 7.5 to 0.8): recovery of the extension (from 45° to 65°) flexion (from 31° to 58°) of the wrist and radial deviation (from 8° to 17°); unchanged ulnar deviation. Improvement of grip strength (from 14.3 to 33.2 kg). In 2 cases it was necessary a second surgery due to the instability and dislocation of the implant (1 dorsal and 1 volar). We have obtained 11 excellent results (average of 86 points), 3 good (average 75 of points) e 2 moderate (65 points) after evaluation with Krimmer test. X-rays showed in 13 cases the restoration of carpal height; in 3 cases this index remain unchanged. The patients returned to their daily activities within an average of 5.4 weeks.

**Discussion** The replacement of proximal pole of scaphoid with APSI is indicated in young patients, when the synthesis or reconstruction of the proximal pole is not possible, while in elderly patients may be a rescue strategy. The pyrocarbon implant requires little sacrifice of bone and it is very well biological tolerated. APSI allows to avoids further deterioration and carpal collapse prevent the degenerative changes to SNAC or SLAC wrist, respecting the biomechanics. Restoration of dorsal intercarpal ligaments is the milestone for the implant stability.

**Conclusions** Replacement of proximal pole of scaphoid with APSI has proved to be a reliable technique for treatment of nonunion and avascular necrosis following fracture of scaphoid, validated by the results with follow-up to more than two years.

### Evaluation of the performance of the TMC Maia implant for the treatment of basal thumb osteoarthritis

M. Stamilla*[1], J. Teissier[2], F. Alkar[2], T. Gaudin[2]

[1]Catania, IT;
[2]Montpellier, FR

**Introduction** With 10 years of experience with use of TMC implants, we created, with a Lepine group, a TMC implant that in our opinion has superior performance to previous. The objective of this study is to evaluate the performance of the TMC Maia implant of the basal thumb osteoarthritis based on a retrospective and monocentre study with a minimum follow-up of 3 years.

**Materials and methods** This is a retrospective study performed. A total of 100 TMC Maia prosthesis for 94 patients (6 bilaterals cases) were included in the study between June 2005 and September 2007. There were 86 females and 8 males with a mean age at the time of surgical procedure of 68.4 years (range 26–79). The mean follow-up was 45.3 months. The main aetiology was basal thumb osteoarthritis.

**Results** Out of these 100 prosthesis 89 were evaluated: 93.6 % of patients were satisfied or highly satisfied. Post-operative pain was greatly improved (evaluated at 0.8 with Alnot score). The mean Kapandji opposition score was at 9.7 and the mean Quick Dash score at 9.9/100. Grip and pinch strength was restored: 5.3 kg for the key-grip and 22.1 kg for the grasp. Survival rate of the TMC Maia prosthesis at 4 years is 95.6 %. Radiographic imaging showed 8.2 % of radio-lucent lines but no sinking of the metacarpal stem, 17.4 % of the trapezial borders with 7.9 % of sinking cups. Complications are rare and only three subsequent revision surgeries where required.

**Discussion** Although not however accepts the use of TMC prosthesis in thumb osteoarthritis, it is true that the alternatives classically advocated is trapeziectomy with suspension and interposition tendinoplasty, the TMC arthrodesis and the pyrocarbon implant. None of these surgical techniques has proven alone to be to efficient and superior to the others in obtaining immediate result, long-term reliability and to be free of complications.

**Conclusions** The Maia prosthesis is an improved version of the Arpe prosthesis and helped resolve the issue of sinking stems while decreasing the trapezial complications and widening the indications thanks to a wide range of collars. The results obtained from our series lead us to recommend the TMC Maia prosthesis as the gold standard treatment in the thumb osteoarthritis, taking in consideration the others surgical techniques in case of failure.

### Anatomical and functional considerations regarding trapezio-metacarpal arthritis and a possible new clinical classification

M. Rubino*, S. Guarino, D. Pagani, V. Sansone, B. Maurilio

Istituto Ortopedico Galeazzi (Milan, IT)

**Introduction** Trapezio-metacarpal (TM) joint laxity is a key factor in the clinical and radiographical development of TM arthritis, and we believe that any new classification should reflect this.

**Materials and methods** We have divided patients suffering from TM joint arthritis into two major groups. Joint laxity is the defining characteristic of the first group, often with only minor radiographical alterations. The patients in this group are typically female. In the second group, the radiographical appearance is closely related to the clinical symptoms, and the patients who fall into this category are normally male, or females who have demanding, manual jobs.

**Results** This new classification aims to provide the specialist with a treatment algorithm, according to the nosological group.

**Discussion** The first major group is that of trapezio-metacarpal instability (TMI), and is divided into three sub-groups. In the first sub-group (TMI1) fall patients with joint laxity, modest but persistent pain, with minimal, or no radiological evidence of TM arthritis. Patients are normally very young women. Treatment consists of a thumb brace to be worn at night. In the second sub-group (TMI2) patients will have suffered from joint laxity for some time, and have significant pain. Radiographs show signs of initial TM arthritis. Surgical treatment is required. In the third sub-group (TMI3) patients will have chronic TM joint laxity with associated hyperextension of the metacarpophalangeal joint, which in the most extreme cases will result in a Z deformity of the thumb. There will be radiographical evidence of advanced TM joint arthritis. The treatment is surgical, but as patients normally report little pain, they will often refuse surgical treatment. The second major group is called trapezio-metacarpal overload (TMO), and comprises patients with TM arthritis resulting from excessive loading, and is divided into two sub-groups. The first sub-group (TMO1) includes both male and female patients who have demanding manual jobs, and who have clear radiographical signs of TM arthritis. Surgical treatment is required to treat the pain and functional limitations that these patients suffer in their working activities. In the second group (TMO2), patients have had a past history of TM pain, but have now achieved an acceptable functional equilibrium. Radiographically, the TM joint is in almost spontaneous arthrodesis. Usually, no specific treatment is required.

**Conclusions** We believe that this classification is a simple method for categorizing patients and indicating treatment—whether conservative or surgical. Furthermore, almost any patient who presents with pain in the TM joint can be classified according to the proposed system.

### *C30–MISCELLANEOUS*

### Protocol of surgery without blood in the hip and knee surgery experience on 2600 patients

M. Scardino*, F. Astore, F. Daria, G. Mazziotta, G. Grappiolo, F. Martorelli, A. Gurgone

Istituto Clinico Humanitas (Milan, IT)

**Introduction** The surgical and anaesthetic techniques developed in recent years have made possible with the use of a medical protocol appropriate to avoid the assignment of blood bags homologous and autologous blood replacement in primary installations of hip and knee. The protocol requires that the patient in a standardized and shared and implemented with the cooperation of all the doctors of the administrative staff and paramedics.
**Materials and methods** One thousand and eight hundreds (1800) patients were selected to be of primary total hip replacement (THR) and 800 patients for primary knee replacement (TKR) all patients included in the protocol of bloodless surgery are followed in three phases, the first for preparing the patient, the second involves anaesthetic and surgical methods to reduce the loss of blood, and the third involves the recovery of blood loss and the control on the prescription of blood and a thromboprophylaxis staff.
**Results** In these two years we have minimized the use of autologous and homologous blood by 67 % the first year and 72 % in the second. The value hb the resignation was not less than 8.5 hb for all patients. No side effects due to the appearance of anaemia have been found.
**Discussion** If a protocol is implemented and shared with all staff participation, and if they are used all the available devices is convenient safe and effective.
**Conclusions** By implementing this protocol on 2600 patients there was an economic saving on expenditure related to the blood of 24,000 Euros the first year and 36,000 Euros in the second.

### Knee osteoarthritis and visco-supplementation: advantages of the only one intra-articular infiltration with a cross linked high molecular weight hyaluronic acid

A. Ammendolia

Dipartimento di Scienze Mediche e Chirurgiche, Università di Catanzaro “Magna Graecia” (Catanzaro, IT)

**Introduction** The osteoarthritis reduces the visco-elastic features of the synovial fluid, modifying the mechanisms of the lubrication and supporting the activity of the inflammatory cells and lytic enzymes. The visco-supplementation using hyaluronic acid demonstrated a real and prolonged efficacy, correlated to his weight and molecular structure. This longitudinal perspective study evaluates the advantages of the only one intra-articular infiltration with a cross linked hyaluronic acid with high molecular weight in the treatment of the knee osteoarthritis.
**Materials and methods** We enrolled 112 patients, 64 women (average age 64.3 ± 7 years) and 48 men (average age 61.2 ± 4 years), affected by knee osteoarthritis II–III grade Kellgren-Lawrence, diagnosed by standard X-rays. We excluded all patients underwent to physical therapy and/or intra-articular infiltrations during the last six months. The clinical evaluation was performed using WOMAC score and all patients were subjected to an intra-articular infiltration with 4 ml of cross linked sodium hyaluronate with a high molecular weight (Monovisc). The mean follow-up was of 11 months (6–18 months). The results were statistically evaluated by Student’s *t* test for paired data.
**Results** In the 62.5 % of the patients we observed a significant reduction of the WOMAC score without any side effect and all were able to be back to the daily activities after 48 h from the infiltration.
**Discussion** The Monovisc has an interaction with the proteoglycans, producing aggregates able to hold a lot of water, promoting: homeostasis restoration of the extracellular matrix and of the cartilage elasticity; pain relief, connecting to the CD44 synovial receptor. It was demonstrated by reported data, these effects depending from the very high molecular weight and from the presence of the cross linked connection, that make the hyaluronic acid able to have an interaction with proteoglycans, pro-inflammatory cells and pain mediators peptides.
**Conclusions** Collected data about 112 patients demonstrate that the only one intra-articular infiltration consent to obtain good clinical results at the follow-up, reducing infection risks always possible with 2 or more infiltrations, like reported in literature (Roos J et al., J Bone Sp, 2004).

### Lower legs myodystrophia: complications and treatment

M. Calogero*[1], J. Negreanu[2], A. Costa[2]

[1]Università degli Studi di Milano (Milan, IT);
[2]Ospedale Niguarda “Ca’ Granda” (Milan, IT)

**Introduction** Calf implants are a procedure to increase size and shape of the calf. I gave preliminary reports in 1988 during the SICPRE Congress in Genova. Three thousands and five hundreds (3500) patients had bilateral or unilateral augmentation for leg ipoplasia with fat transplantation or soft silicone implant or hyaluronic acid. We present the treatment in complications and unsatisfactory results. Under local anaesthesia and with the patient supine, both legs are prepared with the knee joints extended.
**Materials and methods** The incision is made down the popliteal line, if this is possible, on the view incision 2 cm under and 2 cm lateral of tibial border and, if the implant is removed at least 1 year ago, is possible to increase the volume with dissection under the gastrocnemius fascia. Using the Calogero dissector, blunt dissection is carried along the avascular plane, under fascia over the gastrocnemius muscle after break. On the pocket created the implant is inserted. Is possible to achieve a good result with lipofilling if is it obtainable.
**Results** The total series had a complications rate of 11 %: breakdown, seroma, traumatic hematoma, wound problems and dislocation. All of this complications maybe solved.
**Discussion** Calf implants is safe and is a simple surgical technique, the scar is very short and satisfactory results follow when surgical indication is adequate.
**Conclusions** Satisfactory results in complications have usually followed with adequate solutions in technique and implant. The problem rate has dropped markedly with more careful techniques and better patient selection.

### Combined treatment with external fixation and MSC for the treatment of pseudoarthrosis. Clinical observation

F. Verdoni[1], E. Panuccio*[2], A. Memeo[3]

[1]Struttura Dipartimentale Allungamento e Fissazione Esterna degli Arti, Istituto Ortopedico Gaetano Pini (Milan, IT);
[2]Ortopedia Pediatrica, Istituto Ortopedico Gaetano Pini (Milan, IT);
[3]Struttura Complessa di Ortopedia Pediatrica, Istituto Ortopedico Gaetano Pini (Milan, IT)

**Introduction** Aim of this study is to observe the effectiveness of percutaneous mesenchymal stem cell grafting for treatment of pseudoarthrosis.
**Materials and methods** From February 2011 to December 2012, 11 consecutive cases of fractures nonunion were treated with percutaneous autologous bone marrow grafting included 6 males and 5 females, ranging in age from 20 to 71 years, with an average of 40 years. All the cases were traumatic fractures involving 7 of tibia, 4 of femur. In all the cases were performed external fixation before marrow grafting. The time from injury to therapy were from 6 to 12 months, with an average of 8.5 months. The type of nonunion included atrophic in 10 cases, hypertrophic in 1 cases. In 3 cases it was necessary to perform three injections, 2 injection in 5 cases, 1 injection in 3 cases, the interval was 1 month.
**Results** All the 11 cases were followed-up for from 5 to 22 months with an average of 14 months. Two of them were not observed obvious callus after 3 months from the 3rd injection, judged unsuccessful therapy, changed to perform autologous bone grafting the follow-up ended. The other cases obtained bone union during 3 to 12 months with an average of 6.5 months, the follow-up ended at the time of external fixation removal.
**Discussion** Skeletal healing is primarily a biological process, and depends upon cellular response. The most productive source of cells that influence osteogenesis is considered to be autologous marrow. But stable internal or external fixation is the premise. Excessive bone defect, the gap more than 5 mm and misalign requiring rectification is not appropriate for this therapy.
**Conclusions** The technique of percutaneous autologous bone marrow injection provides a very safe, easy and reliable alternative to open bone grafting, especially for early intervention in fracture healing process.

### *C31–KNEE-MISCELLANEOUS I*

### Correction of the flexed knee with the Ilizarov distraction method

A. Kirienko*[1], A. Peccati[1], E. Arnaldi[1], N.M.A. Portinaro[2]

[1]Istituto Clinico Humanitas (Rozzano, IT);
[2]Clinica Ortopedica, Università degli Studi di Milano (Milan, IT)

**Introduction** Rigid flexion of the knee can cause physical disability. In the most severe cases, acute correction of soft tissues deformities or osteotomies can lead to important complications, such as paralysis of the common peroneal nerve, knee subluxation, hypercorrection, skin necrosis and deformity recurrences. Such complication can be avoided performing progressive correction of knee flexion with the Ilizarov techniques.
**Materials and methods** Between 1996 and 2011 we treated 19 knees belonging to 15 patients (9 males and 6 females, meanly aged 28.87 years) with the Ilizarov external fixator. Knee contracture was due to spastic condition of the CNS (4 cases), sequelae of poliomyelitis (3 cases), arthrogryposis (3 cases), hemimelia (3 cases), post-traumatic condition (2 cases). The mean pre-operative ROM was 45°–135°. 4 patients couldn't walk because of bilateral knee flexion.
**Results** Deformities were corrected according to Ilizarov's distraction principles, with the closed method (anterior hinges and posterior distraction); patients affected by arthrogryposis or poliomyelitis also required an osteotomy of the femur or the leg. In 12 cases associated bony deformities were corrected and in 6 cases associated limb length discrepancy was treated. No patients required soft tissues release. The mean time-in-frame was 5 months, and the mean ROM at the frame removal was 0°–30°, while the mean ROM at 1-year follow-up was 0°–75°. All the patients affected by bilateral knee flexion could walk, with or without braces, 1 year after the surgery. Two patients have residual deformities and require braces or orthoses to walk. In 2 cases a mild residual knee instability remains.
**Discussion** Circular external fixation is an effective option for the treatment of knee flexion contracture, and permits the simultaneous correction of associated bony deformities or limb length discrepancy. Recurrences seem to depend on the aetiology of the contracture, not on the chosen treatment and are more frequent in patients with bilateral involvement.
**Conclusions** Progressive soft tissues correction and articular surfaces re-alignment with circular external fixator can be considered as a valid treatment option, especially when soft tissues release or extemporary correction can't be performed. The main advantage of this technique is the possibility to treat associated bony deformities (angular deformities or length discrepancies).

### The effect of anterior cruciate ligament reconstruction on the progression of osteoarthritis

D. Rosa*, G. Ciaramella, G. Balato, V. Crispino, N. Auletta, G. Granata

Dipartimento di Ortopedia, Università Federico II (Naples, IT)

**Introduction** Anterior cruciate ligament rupture (ACL) is one of the most common traumatic injuries of the knee and has a devastating effect on the level of athletic activity and quality of life. ACL injuries often cause knee joint laxity and instability, leading to pain and varying levels of disability. An ACL tear is often associated with meniscal tears, chondral lesions and the onset of post-traumatic osteoarthritis. The objective of this study was to evaluate the incidence of osteoarthritis in the operated knee in comparison with the contralateral knee after a tendon patellar autograft ACL-reconstruction and to evaluate to which level patients regain activity after reconstruction.
**Materials and methods** We retrospectively evaluated 50 patients with complete lesion of the ACL surgically treated with arthroscopic ligament reconstruction with bone-patellar tendon-bone (BTB) autograft. Clinical assessment included anamnestic data, a complete physical examination and testing with KT-1000 arthrometer. All patients were also subjected to evaluation with the International Knee Documentation Committee (IKDC) and Tegner scores pre-injury and post surgery. After surgical treatment, again, have been used cards subjective evaluation KOOS (Knee Injury and Osteoarthritis Outcome Score) and WOMAC and radiographic classification according to Kellgren-Lawrence.
**Results** The mean follow-up was 150 months (from 204 to 96 months). At last follow-up there was a significant increase in clinical score compared with preoperative data. We compared the values of KOOS, Tegner, IKDC and WOMAC rating scales from surgery to the last follow-up. The degree of patient satisfaction was medium–high. We also recorded a resumption of activities at a level almost comparable to that pre-trauma. In most of the cases was found OA grade I-II according to Kellgren-Lawrence.
**Discussion** ACL injuries are often associated with chondral lesions and meniscal tears. Development of post-traumatic OA is a multifactorial event. An instable knee lead to a quickly progression of the cartilage lesion until the onset of a post-traumatic OA. Meniscectomy or meniscal tear accelerate this process. Altered load characteristics, hematoma, subchondral bone bruises, inflammation, synovitis, capsular damage, surgical trauma also contribute to cartilage degeneration. More research needs to be done to determine factors that explain the development of OA and to describe the management of chondral lesions associated with ACL tears.
**Conclusions** ACL reconstruction restores knee stability and allows the patient to resume his o her pre-injury activities but does not prevent the development of post-traumatic knee OA.

### Osteochondral lesions of the knee. A forty-month follow-up of the TRUFIT system

F. Giacco*[1], D. Di Marzo[2], M. Saporito[2], F. Boniforti[2]

[1](Reggio Calabria, IT);
[2](Rome, IT)

**Introduction** The TRUFIT system is a scaffold made of polylactic-co-glycolic acid (50 %), calcium sulphate (40 %) and glycolic acid (10 %), that is used for the treatment of osteochondral lesions. The aim of our study is to evaluate the clinical and functional outcome at 40 months from the transplantation.
**Materials and methods** Nineteen knee osteochondral lesions were treated, 15 on the medial condyle and 4 on the lateral one. Patients were 11 males and 8 females with a mean age of 52 years old (21–63).
**Results** From the 19 patient selected, 12 passed 40 month clinical follow-up. Four cases underwent anterior cruciate ligament (ACL) reconstruction simultaneously to the TRUFIT transplantation. Clinical evaluation of the 12 patient reported a mean Hospital for Special Surgery Score (HSS) of 82 points and no pain on weight-bearing. Complete articular function was assessed by clinical examination and by satisfaction degree of the patients. Radiografic exam showed the presence of the TRUFIT plug in all cases.
**Discussion** Osteochondral lesions treated with a TRUFIT plug transplantation showed no relapse of the lesions, stability of the implant and positive clinical outcome after 40 months follow-up.
**Conclusions** We consider that the TRUFIT plug can be a valid treatment of osteochondral lesions in patient with stable knee joint and intact menisci.

### Anterior cruciate ligament reconstruction in patients over 50

C. Terzaghi*, C. Legnani, E. Borgo, A. Ventura

UOSD Chirurgia Articolare Mininvasiva Istituto Ortopedico G. Pini, Banca Regionale del Tessuto Muscolo-Scheletrico (Milan, IT)

**Introduction** ACL reconstruction in middle-aged people is an issue of debate. Conservative treatment has traditionally been reserved for patients over 50 who do not perform highly demanding activities. With increased average age and life expectancy, activity level is rising among the population and recent studies underline that conservative treatment provides inadequate results, with increased risk of residual instability and chronic associated injuries.
**Materials and methods** Between 2004 and 2009, 45 patients (average age 54.3 years) underwent primary ACL reconstruction with hamstring. In 12 cases medial meniscectomy was performed, in 7 cases lateral meniscectomy. Patients were assessed after an average follow-up of 4.7 years with physical examination (including range of motion, pivot-shift test, and instrumented knee laxity measurement), the KOOS score and the International Knee Documentation Committee scoring system. Standard radiographs were taken, and degenerative changes were graded according to the Ahlbäck radiologic classification of arthritis.
**Results** A significant improvement in knee function and symptoms was reported in most patients, with increased KOOS and IKDC scores ($p < 0.001$). The outcomes of clinical assessment and instrumented laxity testing were clearly improved when compared with preoperative status ($p < 0.001$). The level of osteoarthritis did not statistically increase at follow-up ($p =$ not significant).
**Discussion** The growing body of evidence is changing the approach of surgeons towards surgical treatment in patients aged 50 years and older with ACL deficiency. Physiological age and activity level are more important than chronological age as deciding factors when considering ACL reconstruction. Key symptoms leading to surgery are considered recurrent giving-way episodes during daily activities, which affect the quality of life of the subject, presence of a soft end-point Lachman sign and combined positivity in the pivot-shift test, absence of significant underlying osteoarthritis.
**Conclusions** ACL reconstruction can be successful in appropriately selected, motivated older patients with symptomatic knee instability who want to return to participating in highly demanding sport and recreational activities. Operative treatment showed favourable outcomes in most of the ACL-reconstructed patients with regard to knee stability, osteoarthritis progression, and patient satisfaction in a cohort of subjects aged 50 years or older.

### Platelet-rich plasma intra-articular injections versus visco-supplementation as a treatment for early osteoarthritis: a randomized double blind study

E. Kon, G. Filardo, A. Di Martino*, S. Patella, B. Di Matteo, F. Perdisa, M.L. Merli, M. Marcacci

Laboratorio di Biomeccanica/NaBi, Istituto Ortopedico Rizzoli (Bologna, IT)

**Introduction** The influence of growth factors (GFs) on cartilage repair is not yet widely studied and their application in clinics is still experimental, even if some studies demonstrated a positive influence on cellular proliferation and differentiation. Platelet rich plasma (PRP), a blood product rich in GFs, seems promising for cartilage healing. Aim of this study is to evaluate and compare the efficacy of PRP and visco-supplementation (hyaluronic acid, HA) injections for the treatment of chondropathy or early osteoarthritis of the knee.
**Materials and methods** One hundred and ninety-two patients were enrolled following precise inclusion and exclusion criteria. The study involved patients affected by chondropathy and either early osteoarthritis. All patients underwent an autologous blood harvesting to obtain PRP and then they were randomized to receive either PRP or HA. A cycle of 3 weekly injections was administered blindly. All patients were clinically evaluated at the enrolment and at 2, 6, and 12 months follow-up with IKDC, EQ-VAS, TEGNER, and KOOS scores. ROM, joint effusion, adverse events and patient satisfaction were also recorded. All the evaluations were performed blindly. At the moment 109 patients have reached the final evaluation of 12 months of follow-up.
**Results** No complications or major adverse events occurred among study subjects. Only minor adverse events were detected in some patients, as mild pain reaction and effusion after the injections, in particular in the PRP group, but they lasted for no more than a few days. At the follow-up evaluations, both groups showed a significant improvement in terms of function and quality of life. The preliminary comparison between the outcomes of the two groups showed no statistically significant difference; only a trend slightly favorable for the PRP group was observed in patients with low degree of articular degeneration (Kellgren-Lawrence score up to 2).
**Discussion** PRP is a simple, low cost, and minimally invasive approach to treat chondropathy and early OA. The clinical results of our study are encouraging and suggest this method may be used to treat the degenerative articular pathology of the knee, especially for patients with lo lower degree of articular degeneration. However, the benefit is limited and no significantly better than HA.
**Conclusions** The completion of this study will better clarify the potential of this biologic procedure.

### *C32–TRAUMATOLOGY 9*

### Management of long bone nonunion with the diamond concept: our institutional experience

S. Gudipati*, V. Ciriello, N. Kanakaris, P. Giannoudis

Academic Department of Trauma & Orthopaedic Surgery, University of Leeds, Teaching Hospitals NHS Trust (Leeds, UK)

**Introduction** Recently the Diamond Concept has been advocated in the management of complex aseptic nonunion of long bones which emphasizes the simultaneous implantation of a growth factor, a scaffold, and osteoprogenitor cells with revision of fixation where indicated. The aim of this study was to report the first clinical results on the management of long bone non-unions using the Diamond Concept.
**Materials and methods** This is a prospective study under taken at a regional tertiary referral centre from January 2008 to December 2012. Inclusion criteria where patients with long bone non-unions that were treated with the Diamond Concept. Exclusion criteria were hypertrophic, pathological, and infected nonunions. Data collection included demographics, initial-fracture-pattern, method-of-stabilisation, previous surgical intervention, time-to-revision of fixation, complications, time-to-union, complications and functional outcome. Revision of fixation was carried as appropriate for optimisation of the mechanical environment. Clinical union was defined as painless full weight bearing whereas radiological union was defined as the presence of mature callous bridging to at least 3 cortices. The minimum follow up was 12 months (range 12–32).
**Results** In total 64 patients (34 males) met the inclusion criteria with a mean age of 45 years (17–83) at the time of injury. The majority of the fractures were femoral (54.68 %) followed by tibia (34.38 %), humerus 4.68 %, radius 3.13 %, and clavicle in 3.13 %. The median number of previous interventions was 1 (range 1–5). Eighty-one point twenty-five percent (81.25 %) of patients underwent revision of fixation, in 9.35 % of patients no revision of implant but only grafting was performed of which 2 patients needed dynamization. In all cases biological enhancement consisted of RIA graft, BMP-7 and concentrated bone marrow aspirate. One patient with a femoral nonunion, sustained a mechanical fall after the fixation and had metal work failure and required revision of the fixation and successfully healed 8 months later. One patient with a femoral non-union is still under observation and has 2 cortices healed so far. Three patients developed superficial wound infection (one was MRSA), 1 had DVT, and 1 had HO. The overall success rate was 63/64 unions at a mean time of 6 months (3–12).
**Discussion** In this study a high incidence of union rate was achieved by the application of the Diamond Concept. This strategy allowed restoration of the optimal mechanical and biological environment and facilitated fracture healing.
**Conclusions** The Diamond Concept should be considered in the surgeon's armamentarium especially in cases where difficulty of fracture healing is anticipated.

### The closed osteosynthesis with locked compression nail in the pseudarthrosis of femur, tibia and humerus

G.B. Morace*, A. Dei Rossi, C. Calzavara, P. Esopi

ASL 13 (Dolo, IT)

**Introduction** It is known that the non-consolidation of a fracture may depend on both intrinsic and extrinsic factors, the first related to the patient and the fracture's typology, and the second either to the choice of treatment, or to its incorrect execution.
**Materials and methods** In the last eight years we have treated 22 cases of pseudarthrosis—of which 10 of tibia, 8 of femur, and 4 of humerus—using just the locked compression nail, without attacking the fracture focus. In 15 cases the seat of the lesion was the diaphysis, while in the other 7 it was the diaphyso-metaphyseal area. Seven of 22 fractures had been treated with external holder, 4 with plates and screws, while the remaining 11 had been treated with locked compression nail. Only in 5 cases could the non consolidation be ascribed to the previous exposure, while in the remaining 17 it seemed to be determined by the inadequate coaptation or by a relative instability of the fracture stumps. We therefore chose to proceed to a new osteosynthesis with locked compression nail: after reaming the canal and inserting the locked nail, we used the screwed-in system which, acting on the screw in the dynamic hole, allows the compaction of the stumps. This choice met the requirements of stability and, in many cases, was conditioned by the degree of the soft tissue damage.
**Results** Only in one case was it necessary to revitalize again the focus and to put corticospongiose grafts, while in the remaining cases the fracture seemed to be completely healed between 3 and 7 months.
**Discussion** Yet, the non consolidation of the long bone fractures is often strictly linked to mistakes or inaccuracies in the osteosynthesis technique. It is therefore necessary to face this possibility with methods that may guarantee the best results. The presence of scars and the precarious soft tissues conditions—which are sometimes associated to these lesions—determine the kind of treatment and often contraindicate aggression to the fracture focus.
**Conclusions** The closed osteosynthesis with compression-locked reamed nailing appears as a valid solution in the treatment of the diaphyseal and metadiaphyseal pseudarthrosis of femur, tibia and humerus, both for the undeniable stability advantages and for the reduced invasive property, which is often a necessary condition where cutis and soft tissues are damaged.

### Treatment of bone nonunions with microsurgical corticoperiosteal flap from the medial femoral condyle

M. Guzzini*, C. Civitenga, M. Guidi, C. Dominedò, A. Ferretti

Ospedale S. Andrea, Università "La Sapienza" (Rome, IT)

**Introduction** The corticoperiosteal flap has been described for the treatment of nonunion with bone loss up to 13 cm. This graft is nourished from the descending genicular artery pedicle. It consists of periosteum with a thin layer of outer cortical bone and include the cambium layer, which has a good osteogenic capacity. The aim of this study is to present the results of consecutive series of patients treated by this technique for nonunions, reviewed at minimum follow-up of one year.
**Materials and methods** Nine patients (2 females, 7 males) were surgically treated from January 2011 to March 2012 with a corticoperiosteal flap for bone nonunions. There were 3 carpal scaphoid, 2 humerus, 1 radius, l ulna, 1 tibial bone and 1 femur. The latter was performed with pedicle and the others were free. Among free flaps, the arterial anastomosis was performed with end to side suture in 7 cases and end to end suture in 1 case and in all cases the veins was sutured with end to end suture. All patients were operated by the same surgeon. Every patient but three underwent a previous surgical treatment. We evaluated bone loss, surgical time, microsurgical time, time of bone union, morbidity of the donor site and complications.
**Results** The mean bone loss at the time of surgery was 20.5 mm (6 to 35), the mean surgical time was 6.25 h (4 to 8.5), the mean microsurgical time was 85 min (50 to 120). All patients but one healed. The mean time of bone union was 6.5 months (2 to 11). Failure occurred due to a new injury on the operated arm, resulting in a fracture of the radius with deformity on the site of the graft. No patient reported knee instability or ROM limitations, the mean VAS score was 3 (rate 0–6). The time of recovery to normal walking activity was 2 days (rate 1–4).
**Discussion** The highly osteogenic nature of the periosteum combined with its excellent vascularity after microvascular or pedicled transfer achieves a high success rate in treating difficult nonunions where conventional bone grafts have either failed or are not suitable.
**Conclusions** We believe that, due to the minimal morbidity on the donor site, this flap can be considered also as a first choice to treat patients with bone nonunions. For these matters, we believe this technique has a greater indication in nonunions of the upper limb.

### Use of amniotic membrane in pseudoarthrosis treatment

L. Burri*, G. Bertoni, P. Segnana

Ortopedia e Traumatologia (Treviso, IT)

**Introduction** The amniotic membrane provides a biological support that stimulates the healing of nonunion. These are often difficult to treat and require grafting from the iliac crest or the application of bone substitutes.
**Materials and methods** We treated 15 cases of pseudoarthrosis of long bones with the application of a piece of amniotic membrane after fracture's trephination and stable synthesis. They were 9 males and 6 females, mean age 50 years. We then assessed the results both from radiographic and clinical point of view, the follow-up was of 12 months.
**Results** In all the cases have come to nonunion healing, on average after about 3–4 months. No cases needed other surgical treatment.
**Discussion** The results obtained, albeit with a limited number of cases, indicate that the amniotic membrane may be a factor stimulating the consolidation of the nonunion. The use is simple, but for its characteristics cannot be applied in those cases with loss of substance, if not in combination with other materials.
**Conclusions** We can use the pieces of amniotic membrane as osteogenic stimulus in case of nonunion, with excellent results. This prevents blood loss and complications related to the donor site of the grafting from iliac crest. The size and consistency however restrict the use to those cases which do not require osteoconductive support.

### DIF (dynamic internal fixator)

C. Battiato*[1], E. Sartorello[2]

[1]Azienda Ospedaliera ASUR Marche, Area Vasta 5 (Ascoli Piceno, IT);
[2]Azienda Ospedaliera Regione Veneto (Bassano del Grappa, IT)

**Introduction** The authors herewith show the results obtained in diaphyseal as well as distal and proximal epiphyso-metaphyseal femoral fracture treatment by means of the DIF innovative system. Such system belongs to the so-called internal fixators category, i.e. angular stability plates which have been modified so as to become dynamic, when required. Within the action limit of the external fixators, this system, therefore, allows modulating the need to increase compression. The biomechanics osteo-implant system theory is discussed and the clinical and radiographic results obtained in fractures and pseudoarthrosis treatment are shown.
**Materials and methods** The DIF was applied to 10 patients with different femoral fracture with patterns at risk of pseudoarthrosis. In all these patients the system was dynamized at a period between the 40th and the 60th day after the implantation. X-rays were taken every 30 days. The follow-up was for all the 10 patients carried on until the radiographic evidence of bone repair.
**Results** In all the patients we observed a fast bone recovery and no case of implant failure.
**Discussion** We observed that this internal fixator modified to become dynamic, can offer one more option to the bone healing process. Within the action limit of the external fixators, this system, therefore, allows modulating the need to increase compression.
**Conclusions** We think that DIF allows the possibility of increasing fracture site compression when charging during the healing process. Furthermore, the possibility of a plate-sliding system discharges the angular flexion pressure (flexing movement) becoming a sliding movement and thus an axial compression.

### Atrophic forearm nonunion: analysis on 44 cases

M. Rampoldi[1], R. Viscito[2]

[1]Ospedale CTO (Rome, IT);
[2]Policlinico Tor Vergata, Università Roma II (Rome, IT)

**Introduction** Fracture healing, functional and anatomical recovery were studied in an homogeneous group of atrophic forearm nonunion treated by rigid internal fixation and autologous bone grafts eventually associated with osteoinductive factors. Aim of the study is to evaluate the results of this method and the influence of osteoinductive adjuvants in fracture healing.
**Materials and methods** Forty-four atrophic nonunion of the forearm (31 males and 13 females, mean age 47 years) were operated by internal rigid fixation with plates and autologous bone graft taken from the iliac crest. In 5 cases a contemporary subtraction osteotomy of the ulna was performed. Nineteen patients had associated osteoinductive factors (BMP-7, demineralised bone matrix DBX, concentrated bone marrow). In 26 cases both bones were affected (59 %) while in 18 (41 %) only the radius or the ulna were interested. All patients had at least one previous surgical procedure; initial fracture was closed in 30 cases (68 %) and open (Gustilo II and III) in 14 (32 %). Mean time between the first operation and the treatment of the nonunion was 13 months.
**Results** All fractures united in 3 to 9 months. Fractures treated with associated adjuvant factors healed in 4 to 7 months. Complete functional recovery was achieved in 80 % of initially closed fractures but only in 37 % of open fractures. Incongruence of distal radio-ulnar relationship was observed in 5 cases (11 %); incomplete forearm rotation was noted in 8 cases. No complications were referred on the bone graft donor site.
**Conclusions** Stable plate fixation associated to autologous bone graft lead to unite the majority of atrophic forearm non-union without complications. Use of osteoinductive factors may reduce the required amount of bone graft and shorten the time of union but its value is not yet demonstrated. Bone shortening, up to 5 cm, can be functionally well tolerated. Functional recovery seems dependant on initial lesion and recovery of distal radio-ulnar joint congruency.

### *C33–TRAUMATOLOGY 10*

### Tibial pilon nonunion: classification

M. Massobrio*, G. Pellicanò, P. Albanese, G. Antonietti, U. Bianco, G. Corsi

Dipartimento Scienze Anatomiche, Istologiche, Medico-Legali, dell'Apparato Locomotore, Università "La Sapienza" (Rome, IT)

**Introduction** We identified the anatomical-radiographic features of tibial pilon nonunion in order to create a classification (not existing at the moment) for diagnostic and therapeutic purposes.
**Materials and methods** We studied the clinical records of the patients who were operated on for tibial fracture from 2001 and 2011 (total 1015 patients). We picked the fractures who reached the distal joint surface (57 patients, 5 % of all tibial fractures): the tibial pilon fractures (type B and C, Muller classification). We also considered the ones associated to fibular fractures. Tibial pilon nonunion was diagnosed in 26 patients, mean age 48 years old (17–80), 11 rights tibias, 15 left tibias, 16 men, and 10 women. In all cases diagnosis was made through X-rays and CT scans.
**Results** In 10 patients the nonunion was located in the metaphyseal-diaphyseal region, either atrophic or hypertrophic. In 4 cases the nonunion was located in the medial malleolus, in the anterior and posterior tibial margin, and in the lateral malleolus (total nonunion). Two patients with tibial pilon fractures, later presented with lateral malleolus nonunion only, 4 with tibial malleolus nonunion. The nonunion affected the posteroinferior tibial margin (3rd malleolus) in 5 cases, the anteroinferior tibial margin in 5 cases. Some patients presented 2 or more separated sites of nonunion. Syndesmosis lesions were associated in 2 cases to posterior malleolus nonunion, in 3 cases to anterior margin nonunion.
**Discussion** Our study confirms the low incidence of this complication in tibial fractures, nevertheless it has a high incidence comparing to tibial pilon fractures. This can be explained by the particular anatomy of the distal tibia that promotes vascular complications and open fractures which expose the fracture hematoma and scatters its osteogenetic stock. Another cause is the missed identification of the tibial pilon fracture, sometimes mistaken for a bimalleolar fracture. The routine execution of CT scans with 3D reconstruction is essential.
**Conclusions** The distal tibial articular fracture can evolve in nonunion, with partial or total involvement of the distal tibia's components, never described before. The metaphyseal-diaphyseal nonunion is the most frequent in the distal tibial fractures. Nevertheless we must consider all consolidation defects of the tibial pilon such as: 1. lateral malleolus nonunion; 2. medial malleolus nonunion; 3. tibial anteroinferior margin nonunion; 4. tibial posteroinferior margin nonunion; 5. metaphyseal-diaphyseal nonunion; 6. total nonunion of the tibial pilon.

### Aseptic nonunion of femur: intramedullary nail or plate and massive bone graft?

M. Di Liddo*, M. Mosca, A. Shehu, S. Giannini

Istituto Ortopedico Rizzoli (Bologna, IT)

**Introduction** When a fracture has no autonomous capacity to heal 9 months after injury and shows no clinical and radiographic signs of consolidation, we have a nonunion. Nonunion represents the failure of the system of surgical reduction and synthesis of a fracture which leads to the failure evolution of reparative callus in bone tissue.
**Materials and methods** In the clinic at the Rizzoli Orthopaedic Institute in the past 5 years were treated 31 patients with aseptic nonunions of the femur in 15 cases using the synthesis with nail and in 16 cases the synthesis with plate and massive bone graft. Patients with nonunion on nail treated with new nailing, were subjected to removal of the nail, reaming of the canal with larger diameters and application of adequate diameter nails preferably without opening the focus of nonunion. Patients with nonunion on plate were subjected to the opening of the focus of nonunion and new synthesis preferably with lateral plate and medial massive bone graft cortex.
**Results** In 15 nonunion treated with nail in two cases there has not been the consolidation, while in 13 cases occurred on average after 4 months. In the 16 nonunions treated with plate and cortical graft the consolidation occurred in all the cases, on average after 5 months, but in 3 cases it was necessary to perform a new synthesis with plate and cortical graft.
**Discussion** The nail technique is considered in the literature the most effective for the treatment of aseptic nonunion of the femur, is technically easier but hides some pitfalls. In particular when the nonunion is localized in the metaphyseal or when multiple fragments expand the canal section. The plate with cortical graft show great stability of the synthesis and biological effect, ensuring very good results, but the technique is more complex, bleeding and limb shortening are superior and should be avoided by carefully rupture of cortical graft because otherwise there would be a loss of the stability of the system.
**Conclusions** the nail is the means of synthesis to prefer in the treatment of nonunions of the femur, it should always performed one locking static and dynamic one. When you are forced to open the focus of nonunion, in the atrophic forms and in the metaphyseal or when there is an enlargement of the intramedullary canal is to be preferred rigid synthesis with plate and cortical graft.

### *C34–TRAUMATOLOGY 11*

### Bone marrow concentrate as an osteogenic support in the treatment of forearm nonunions

E. Gasbarra*, C. Rao, M. Feola, V. Tempesta, U. Tarantino

Università Tor Vergata (Rome, IT)

**Introduction** The aseptic nonunion of the forearm represents a complication of fractures of the radius and/or ulna, with a high incidence in the case of open fractures, in presence of co-morbidities, following early mobilization or inadequate reduction or osteosynthesis during the first surgery. This study assessed consolidation, restoration of anatomy and functional recovery in a group of patients with atrophic nonunion of forearm treated by removal of the hardware, debridement of the site of the fracture, apposition of bone substitutes and stem cells taken from the iliac crest and new osteosynthesis.
**Materials and methods** From October 2011 to December 2012 we performed 8 surgical procedures for nonunion of the forearm in 7 patients (7 men, including one bilateral case) with a mean age of 32.4 years (range 29–38) after a mean interval from fracture of 17 months (range 5–60). Nonunions involved only one bone, ulna in 62.5 % of cases and radius in 37.5 %. The patients had prior treatment with plates (4 cases), intramedullary systems (2 cases) or both (2 cases); the initial fracture was closed in 5 cases (62.5 %) and open in 3 (37.5 %). The bone marrow taken from iliac crest was processed through an automated system that allowed the separation of the fraction with higher content of stem cells.
**Results** All fractures healed between 3 and 6 months, with radiographic signs of consolidation. A deficiency of pronation and supination was found in 2 cases that underwent a fracture of both bones in the distal third of the forearm and with persistent pain at wrist joint. In 6 patients (85.7 %) we observed an almost complete functional recovery. There were no complications at the site of surgery or during the bone marrow aspiration.

**Discussion** In literature, several techniques are described for the treatment of nonunion of the forearm, also contemplating the use of autologous grafts, allografts with the support of platelets and bone marrow. These latter represent an excellent opportunity for healing ensuring an osteogenic support to the process of consolidation.

**Conclusions** A stable fixation with plates is able to lead to consolidation without complications in most cases of fractures of the forearm. The use of concentrated bone marrow has given excellent results in the treatment of nonunion of the forearm so it suggests that it can be used also in primary osteosynthesis.

### Distraction osteosynthesis in the treatment of hypertrophic nonunions of the long bones

B. Bertani*, L. Pedrotti, G. Tuvo, C. Chittaro, M. Bettani, S. Lucanto, F. De Rosa, R. Mora

Clinica Ortopedica e Traumatologica, Polo Universitario “Città di Pavia” (Pavia, IT)

**Introduction** Hypertrophic nonunion results from mechanical failure of fracture treatment performed by osteosynthesis systems. Inter-fragmentary pathological movements lead to bulky periosteal callus formation, although they hamper the development of the endosteal callus. Nevertheless it is histologically demonstrated that bone extremities have a rich vascularisation, which makes the nonunion viable and still able to induce the healing biological response.

**Materials and methods** From 2000 to 2010 we treated 80 patients (52 men and 28 women), ranging in age from 20 and 68 years, affected by hypertrophic aseptic nonunion, associated with shortening, with or without angular deformity. A total of 14 humeri, 22 femurs and 44 tibias were reviewed. Five to forty-two months occurred between trauma date and treatment starting time. In all patient circular external osteosynthesis was carried out with the Ilizarov apparatus. The first phase consisted of gradual distraction of the nonunion site (1 mm per day) in order to achieve deformity correction. In a second phase treatment was discharged by periodical distraction (0.5–1 mm per day each 10 days). Clinical and instrumental (radiographic, ultrasonographic, extensimetric and histological) investigations were carried out on the 15th and 30th post-operative day, and from then on, every 30 days up to radiographic healing.

**Results** In a lapse time between 3 and 10 months, all nonunions recovered and the bone anatomical axis was restored. Complications encountered throughout the treatment included superficial (41 cases) and deep (8 cases) pin tract infection, transient peroneal nerve palsy (1 case).

**Discussion** Distraction is considered to be a mechanical stimulus which induces bone differentiation in biologically active nonunion (mechano-transduction), in total agreement with Ilizarov tension-stress theory. Molecular basis still remain unclear, even if it is well known that tension-stress leads to fibrous area cells sprain, along the direction of its application. Cells shape changes, resulting similar to fibroblasts; a modification occurs with expression of bone matrix RNA proteins, cells release pro-inflammatory cytokines (IL-6) and morphogenetic proteins able to induce precursor osteogenic cells differentiation.

**Conclusions** Distraction osteosynthesis, according to Ilizarov method, provides an efficient stimulus to induce nonunion tissue direct ossification.

### *C35–ARTHROSCOPY*

### Treatment of osteochondritis dissecans of the knee using bone marrow mononucleated cells transplantation by mean of arthroscopy (one step technique): results and RM-T2 mapping evaluation

L. Ramponi*, R. Buda, F. Vannini, M. Cavallo, S. Natali, A. Olivieri, G. Pagliazzi, S. Giannini

Istituto Ortopedico Rizzoli (Bologna, IT)

**Introduction** Osteochondritis dissecans of the knee (OCD) is an osteochondral lesion who interest femoral condyle in adolescent age ore young adults and the ideal management remains controversial. During the juvenile age non surgical treatment could be adequate, nevertheless surgery is often required. The rationale of bone marrow derived cells transplantation (BMDCT) is based on the capability of the multipotent cells, along with their microenvironment to differentiate and regenerate both the cartilaginous and subchondral bone layer and may be adequate for osteochondritis dissecans regeneration. The goal of this study is to evaluate and report the clinical and MRI findings for the treatment of osteochondritis dissecans of the knee with BMDCT transplantation.

**Materials and methods** Between 2006 and 2011, 14 patients (27.6 $\pm$ 15 years) affected by OCD of the medial femoral condyle, were treated between by BMDCT. Patient evaluation included IKDC and KOOS score, X-rays and MRI (Mocart score) preoperatively and at 42.4 $\pm$ 20.4 months follow-up. At the final follow-up qualitative evaluation by T2 mapping was also performed. The bone marrow-derived cells were harvested from posterior iliac crest, concentrated in the operatory room, loaded on a hyaluronic acid scaffold with platelet-gel and arthroscopically implanted in the same surgical session (one-step).

**Results** IKDC score improved from 48.2 $\pm$ 11.2 to 78.3 $\pm$ 16.0 at final follow-up ($p < 0.0005$) and KOOS score from 60.2 $\pm$ 12.9 to 83.4 $\pm$ 18.3 ($p < 0.0005$). MRI (Mocart score) showed a satisfactory regeneration of the lesion site, while T2 mapping confirmed the presence of hyaline cartilage at the reparative site.

**Discussion** The results obtained in this series treated with bone marrow derived cells transplantation are very satisfying under clinical and imaging aspects. MRI-T2 mapping showed a regenerated hyaline cartilage at the reparative site with regeneration of subchondral bone.

**Conclusions** The one step technique demonstrated to be a good and reliable option for the treatment of OCD, furthermore it overcomes many disadvantages of the techniques traditionally used. A larger case series and long term evaluation are needed in order to confirm the validity of the procedure.

### Arthroscopic revision surgery in shoulder instability

S. De Giorgi*[1], A. Castagna[2], B. Moretti[1]

[1]Università degli Studi di Bari (Bari, IT);
[2]Istituto Clinico Humanitas Rozzano (Milan, IT)

**Introduction** After an arthroscopic shoulder stabilization, a risk, even if low of recurrence does exist. The aim of this study was to evaluate the role of arthroscopic capsuloplasty in the treatment of failed primary arthroscopic treatment of glenohumeral instability.

**Materials and methods** We retrospectively examined at a minimum of 3-years follow-up (mean 4 years and 8 months), 22 patients who underwent arthroscopic treatment between 1999 and 2007 who had recurrent anterior shoulder instability with a post-surgical failure. A multivariate statistical analysis was performed with SPSS 11.0 (SPSS Inc, Chicago IL) to evaluate mean difference with a confidence interval of 95 % ($p < 0.05$) in comparison between intra-operative pathological findings (Bankart, ALPSA, SLAP, capsular lesion, drive through sign), age of patients at first dislocation, at first surgery, number of initial dislocations, interval between first dislocation and first surgery, number of dislocations between first and second surgery, age at second surgery and ROWE score at final follow-up.
**Results** We observed after revision surgery an overall failure rate of 8/22 (36.4 %) including frank dislocations, subluxations and also apprehension that seriously inhibit the patient's quality of life. Data analysis showed that 4/22 (18.2 %) patients had true dislocations, whereas 1/22 patient (4.6 %) had subluxations (clunks) and 3/22 patients (13.6 %) had severe painful apprehension. Mean score on follow-up using the Rowe assessment was 74 points. No significant differences were observed between the age of patients at the time of the first dislocation, at first surgery, number of initial dislocations, interval between first dislocation and first surgery, number of dislocations between first and second surgery, age at second surgery and the Rowe score at final follow-up. With a confidence interval of 95 % ($p < 0.05$), no correlation was found between the intra-operative pathological findings (Bankart lesion, ALPSA lesion, SLAP lesion, PHAGL lesion, drive-through sign), and the Rowe score at the final follow-up.
**Discussion** In our sample the total percentage of recurrence was 36.4 %, more than the reported studies by Kim, Neri, Creighton, Franceschi, Barnes. Our study is the first one considering the residual painful apprehension of the shoulder. Without it, the percentage of recurrence decreases to 22.8 % (18.2 % of true dislocations and 4.6 % of subluxations), more similar to the previous reported results.
**Conclusions** Shoulder arthroscopy allows good identification of intra-articular lesions. In the absence of severe glenoid or humeral bone loss, HAGL lesions and poor tissue quality, it is a fair surgical option in the treatment of previous surgical failures. The rate of failure remains superior to the primary arthroscopic treatment.

### Predisposing factors for recurrence of shoulder instability after arthroscopic stabilization treatment

R. Loschi*[1], G. Matino[1], G. Leo[1], P. Baudi[1], E. Rossi Urtoler[2], F. Catani[1]

[1]Azienda Policlinico-Universitaria (Modena, IT);
[2]Ospedale "Ramazzini" (Carpi, Modena, IT)

**Introduction** The recurrence of shoulder instability after arthroscopic stabilization is a major mid-long term complication. The aim of this study is to analyze the incidence of risk factors in the failure of this technique in order to indicate the correct surgical treatment.
**Materials and methods** From November 2006 to November 2009, 62 patients with anterior instability of the shoulder by arthroscopic stabilization with anchor were treated. Due to the loss of certain demographics in the wake of recent earthquakes, only 37 patients (30 males and 7 females), could be assessed clinically. The mean age was 27 years, with a mean follow-up of 61 months. Patients were evaluated by the ROWE, UCLA, DASH and ISIS scoring systems. Analysis consisted of predisposing factors (age, type of sports activity, joint laxity) the type of glenoid bone injury (by PICO method with TC-2D), the type of Hill Sachs lesion (with TC 3-D), and the type of suture performed.
**Results** We obtained a mean of 84 points by ROWE scoring, 32 points with the UCLA scoring system, and DASH scoring averaged 3.4 points. The recurrence rate was 29.6 % at 5 years follow-up. Analysis with Fisher's exact test showed that the statistically significant factors for recurrence ($p < 0.05$) were associated with the patient's level of activity and the use of absorbable anchors. Other factors to consider, although results were not statistically significant, are: the width and position of Hill Sachs lesion associated with Bony Bankart > 15 %, and age less than 20 years.
**Discussion** Recurrent dislocation after arthroscopic treatment can be reduced if predisposing factors are recognized in the pre-operative evaluation. Our study, in concordance with the international literature, highlights the importance of the type of bone lesion and its localization. Particular importance is given to the level of sporting activity carried out by the patient and the use of absorbable anchors.
**Conclusions** The identification of risk factors is essential to reduce the incidence of recurrence to mid-long term. The careful study of bone lesions and identification of specific risk factors (age, level of activity) have changed our restorative approach, encouraging the use of remplissage Hill Sachs lesion when associated with a glenoid defect and the use of anchors in inert or osteoinductive material.

### Arthroscopic tensioning of medial retinaculum in adolescent patients affected by chronic patellar instability

M. Mariani, D. Mascello*, O. Mazza, M. Crostelli

(Rome, IT)

**Introduction** In our series we evaluated the efficacy of surgical arthroscopic tensioning of medial retinaculum in adolescent patients affected by anterior knee pain due to chronic patellar instability, without patellar dislocation.
**Materials and methods** Before surgery all patients have been treated by physical therapy for at least one year. From February 2008 to November 2010 we operated 8 adolescent patients (7 females, 1 male, mean age at surgery 15 years and 2 months, range 14–17 years) affected by anterior knee pain due to chronic patellar instability, by surgical tensioning of medial retinaculum, using arthroscopic plication by PDS wire. No patient ever had any patellar dislocation. In 2 patients with severe lateral patellar tilt we associated lateral release. Three patients have been operated bilaterally. Patients have been evaluated by clinical exam evaluating patella mobility by medial–lateral glide test. Lysholm and Tegener evaluating tests have been completed before surgery and at follow-up. All patients have been examined before surgery by knees X-rays (standard anterior-posterior and lateral views and patella axial view with knee bent to 45°) and by knee MRI to exclude intra-articular associated lesions.
**Results** At mean 34 months follow-up (range 27–60 months), according to Lysholm evaluating scale, we obtained 6 excellent results (75 %), 1 good result (12.5 %) and 1 fair result (12.5 %), enhancing mean score from 52 to 91 points. Activity level, according to Tegener evaluating scale, improved from mean 3.1 to mean 6. Later glide test reduced from mean 2.9 to mean 1.2 and medial glide test reduced from mean 1.9 to mean 1.2. We had two minor complications (1 hemarthrosis and 1 granuloma due to PDS wire), that solved spontaneously in a few months.
**Discussion** Medial retinaculum arthroscopic tensioning is a surgical procedure with sharp and narrow indications. We use this treatment in patients affected by chronic patellar instability without patellar dislocation. We treat adolescent patients only after skeletal growth completion.
**Conclusions** Our cases, even if limited in number of patients and in medium-term follow-up, underline the efficacy of surgery, that obtained clinical improvement and activity level enhancement in all patients.

### *C36–KNEE 6*

### Autologous chondrocytes implantation (ACI) of second generation: clinical and radiological long term follow-up

D. Rosa*, G. Ciaramella, R. Esposito, G. Granata, N. Auletta, S.l. Di Donato, G. Balato

Dipartimento di Ortopedia, Università Federico II (Neaples, IT)

**Introduction** The treatment of chondral lesions is still a great challenge to an orthopedic surgeon. The autologous chondrocytes implantation, offers a real opportunity of reconstruction of chondral defects with a fabric with characteristics comparable to those of hyaline cartilage.

**Materials and methods** In our department we performed 20 ACI of II generation. The series includes 17 males and 3 females. In 40 % of cases it was a lesion of traumatic origin, 12 patients were actually suffering from osteochondritis dissecans (OCD). All patients were assessed by IKDC, Knee Examination Form and Tegner activity score. Postoperatively was also used the evaluation board KOOS (Kneeinjury and Osteoarthritis Outcome Score). Ten patients underwent MRI to check regularly that they have been evaluated by an experienced radiologist with the scale Mocart (Magnetic Resonance Observation of Cartilage Repair Tissue).

**Results** The mean follow-up was 86 months (range 13630 months). At the last follow-up there was a significant increase in clinical score compared with preoperative data. We compared values of Mocart score of MRI performed at 12 months (range 630 months) from the implantation and at the last follow-up. A significant increase in its value has been recorded.

**Discussion** The proper ability to repair cartilage is very scarce since it is devoid of blood supply, lymphatic and nervous, so the surgical management of these lesions is a continuing problem. MRI reveals the main investigation in diagnosis and long term evaluation of patients surgically treated.

**Conclusions** Our series, according to literature, highlight the clinical and functional efficacy of second generation autologous chondrocyte implantation in treatment of cartilage lesions.

### The orthobiology in the treatment of the monocompartmental medial arthrosis of the knee: results from a distance

A. Siclari*, M. Piras, T. Aramo, M. Vigna Suria, E. Boux

Ospedale Infermi (Biella, IT)

**Introduction** The orthobiology is an innovative sector of the orthopaedics which privileges the tissue reconstruction using the tissue own resources. Since lot of years, there are two different kind of treatment for the knee monocompartmental arthrosis: the prosthetic substitution and the osteotomy. One of the problems of the latter was that the present cartilage lesion showed only a partial improvement with a frequent arthrosic evolution. To prevent this evolution a treatment of the cartilage lesion with AMIC technique has been added to the additional medial osteotomy for the treatment of the mono-compartmental arthrosis.

**Materials an methods** From January 2007 to December 2009, 45 cases of monocompartmental arthrosis of the knee have been treated with shinbone's osteotomy high in addiction with plate and wedge with medial base in tricalcium phosphate by the OTIS company (SBM France); as cartilage scaffold it has been used the chondrotissue (BioTissue AG, Switzerland). The average age was of 54.5 years (43–69). The follow-up varies from 35 to 52 months with an average of 42 months. The preoperative radiographic evaluation has been conducted measuring first the MAD (mechanical axis deviation), then studying the mLDFA, the MPTA and the JLCA. The endoarticular evaluation of the cartilage lesion has been conducted with RMN. The patients accepted to the procedure were all patients with shinbone's originated alterations of the MAD and chondral lesions of 3$^{rd}$ degree or more, pain from almost 3 months, not improved with conservative treatments. For the clinical follow-up it has been used the KOOS with monthly controls up to 6 months, then semestral; the radiological follow-up has been made annually with MAD, MPTA and patella's height index measuring.

**Results** The KOOS' average preoperative value has increased from 48.8 to 92.7 after 3 years. The MAD's average value has passed from 21 to 11 and the MPTA from 80 to 91. In the treated cases it has never been discovered mechanical failures of the synthesis mean, healing difficulties or iatrogenic fractures. The articular evaluation has been made with fibroarthroscopy after a year from the treatment.

**Discussion** The clinical and radiographical results of this treatment were good, with an evident increase of the medial articular line space after 3 years from the treatment.

**Conclusions** To add to the classical procedure of the additional medial osteotomy an endoarticular treatment of the cartilage lesion seems to give good perspectives for a non prosthetic treatment of the monocompartmental medial arthrosis of the knee.

### Low field MRI in the knee cartilage pathology

D. Rosa*[1], G. Balato[1], E. Soscia[2], S.L. Di Donato[1], A. Di Meo[1], V. Crispino[1]

[1]Dipartimento di Ortopedia, Università Federico II (Neaples, IT);
[2]Istituto di Biostrutture e Bioimmagini IBB, CNR (Neaples, IT)

**Introduction** The cartilage injury of the knee represents a extremely frequent pathology and may be the initial phase of an arthritic and, besides, can cause painful symptoms. In fact, the diagnosis of these lesions is still very complex due to the low sensitivity of instrumental examinations, so, the detection of chondral lesions is occasional during arthroscopies in knees suffering from other joint associated diseases. MRI investigation is very sensitive and specific in the diagnosis of meniscal and ligamentous injury, but does not provide sufficient diagnostic confidence for cartilage, especially if the examination is not targeted with specific sequences. The aim of our study is to validate the qualitative sequences for cartilage obtained from low-field equipment that have lower costs and a better distribution on the territory, by determining the relations between changes of the extracellular matrix and the MRI scans obtained in vivo.

**Materials and methods** We used an examination of RM with dedicated equipment to echo sequences with various gradients weighted in T1 and T2 in order to generate relaxometric maps (T2 and T2 rho) for the evaluation of changes in water and in 10 patients with arthrosis and waiting for joint replacement surgery. Intra-operatively were taken from the resection of the lateral tibial plateau osteochondral samples. These specimens were examined histologically by assessing the degree of cartilage degeneration, the content of GAG (glycosaminoglycan) and the content of water. These parameters were compared with values obtained from the relaxometric maps.

**Results** There is a significant correlation between the values of the relaxometric maps and the histological grade of cartilage degeneration, the content of GAG and the water content.

**Discussion** Using an MRI exam performed with dedicated equipment at low field, which have lower operating costs and greater compliance by patients, it also offers the possibility of early diagnosis of lowgrade cartilage lesions and also a closer examination and non-invasive evolution of these lesions.
**Conclusions** The present study shows that the maps obtained with low field equipment can be used as a routine technique in the study of non-invasive cartilage lesions, in order to expand the number of diagnostic centres able to identify correctly a cartilage lesion with the tools currently available.

### *C37–KNEE-ACL I*

### Clinical, instrumental and biomechanical 10-year follow-up of patients who underwent anterior cruciate ligament reconstruction with the LARS synthetic ligament

E. Sebastiani*[1], E. Pennesi[2], G. Placella[1], F. Manfreda[2], A. Speziali[1], M. Tei[1], G. Potalivo[4], G. Cerulli[3]

[1]Clinica Ortopedica e Traumatologica, Università degli Studi di Perugia (Perugia, IT);
[2]Università degli Studi di Perugia (Perugia, IT);
[3]Nicola's Foundation ONLUS, Let People Move Research Institute, International Orthopaedic and Traumatologic Institute, Università degli Studi di Perugia, (Perugia-Arezzo, IT);
[4]Let People Move Research Institute, Nicola's Foundation ONLUS (Arezzo, IT)

**Introduction** The use of synthetic material for ACL reconstruction has been proposed since the early 80's. High failure rates have been reported in literature, hence this procedure was not popular in the following decade. The LARS synthetic ligament (ligament advanced reinforcement system) is classified as a second generation ligament designed to eliminate the most common complications such as the risk of rupture, synovitis and lack of fibroblast repopulation. The aim of this study was to evaluate, at a long-term follow-up (more than 10 years), whether the good results obtained at the short term visits, had been maintained.
**Materials and methods** Thirty-one patients, between 45 and 66 years of age (mean age 56.4 years) were retrospectively enrolled; all had undergone surgery for ACL reconstruction with the LARS synthetic ligament between 2000 and 2004; they have been evaluated clinically, radiographically and using evaluation scores such as Lysholm Score, IKDC, and Tegner Activity Scale. A biomechanical assessment was performed for each patient including gait analysis, stabilometry, isokinetic evaluation of the muscle of the lower limb and KT-1000.
**Results** The outcome of the follow-up has been encouraging: the average Lysholm score obtained was 96.06, the average IKDC was 90.97. The Tegner Activity Scale decreased from 4.1 (2–9) pre-operatively to a current 5.8 (4–10). Good–excellent results were also obtained at clinical examination and at the biomechanical evaluation. Three of the 31 patients had to undergo further surgery for rupture of the synthetic ligament (one of these bilateral). Synovitis or other major complications were not observed.
**Discussion** The use of a synthetic ligament for ACL reconstruction has a limited indication but it avoids the complications of donor site morbidity and allows faster recovery. The improvement of new materials, in terms of biocompatibility and resistance, as well as the improvement in surgical techniques, has lead to the use of synthetic materials to be proposed again, with satisfactory results.
**Conclusions** The synthetic ligament can be considered a viable alternative to autologous or heterologous graft for ACL reconstruction as long as the indications are correctly interpreted. With an average follow-up of more than 10 years, we have shown how the results are encouraging with minimal complications, and currently the risk of synovitis is almost nil, as already demonstrated in the literature.

### Kinesthetic recovery during the first 12 months after ACL reconstruction in athletes

C. Corradini, D. Tradati*, S. Pasqualotto, F. Boisio, C. Verdoia

Clinica Ortopedica, Università degli Studi di Milano (Milan, IT)

**Introduction** Neurosensorial ACL properties characterize joint stability in both trauma and following functional recovery. Despite of the evolution of surgical techniques no recently studies have been performed in this direction. In particular, the date of return to sport is still fixed at 6 months after the surgery and the proprioceptive recovering mechanisms and events happening during this period haven't been fully explained yet. The purpose of this study was to measure and evaluate the trend of the kinesthetic ability of the subjects who undergone ACL reconstruction during the first 12 months after surgery.
**Materials and methods** We examined 48 athletes between 18 and 37 years old, with isolated ACL rupture. All of them underwent reconstruction using autologous STG. Twenty athletes without previous knee distortions were considered as control group. Kinesthetic ability evaluations were performed pre-operatively and at 2, 3, 6 and 12 months after surgery by means of the kinesthetic stabilometric platform KAT-2000 (Breg, Ca, USA). The test protocol lies in the execution of two monopodalic and two bipodalic tests.
**Results** Postoperatively there was a significant improvement in all stabilometric tests. During the first 3 months tests we observed the greatest kinesthetic improvement (70 % of the total recover, $p < 0.01$). At 6 months no statistically significant differences between reconstructed patients and control group were detected. In pre-operative single stance tests no statistically significant differences were highlighted between healthy and injured limb; however in post-operative tests healthy leg has shown an earlier improvement than the injured one despite of having followed a similar trend. We also observed an high pre-operative variability in distribution, which started decreasing at 2 months until the control at 12 months.
**Discussion** ACL injury results in a functional instability of the knee with abnormal kinesthetic values, and consequently of the motor pattern; this is highlighted by the stabilometric data related to the healthy limb which follow the progress of the contralateral. The data seem to suggest that, re-evaluating the stabilometry of an athlete who undergone ACL reconstruction, we can allow a proper functional recovery planning in order to anticipate a return to sport even before the 6 months reported in the literature.
**Conclusions** The clinical relevance of this study lies in the demonstration the importance of ACL reconstruction in order to achieve a complete functional recovery and the role of the kinesthetic evaluation in order to establish the correct timing for a proper return to the sport activity.

### ACL reconstruction anatomic all-inside technique: surgical technique and results

A. Ruffilli*, R. Buda, G. Pagliazzi, M. Baldassarri, E. Ferranti Calderoni, F. Castagnini, S. Maraldi, S. Giannini

Istituto Ortopedico Rizzoli (Bologna, IT)

**Introduction** Reconstruction of the anterior cruciate ligament (ACL) has become a common procedure with many techniques described in the literature. Recently technical improvements led to the development of minimally invasive techniques capable to obtain anatomic ACL reconstruction. Aim of this study is to describe the clinical results obtained with an anatomic, single bundle all-inside technique using triplicated or quadruplicated autologous semitendinosus tendon.

**Materials and methods** Thirty-two male athletes with unilateral ACL injury were enrolled in the study. The technique encompassed the unconstrained creation of an half tunnel both on the tibial and on the femoral side using guide pins that became retrograde drills. Femoral and tibial fixation was achieved with second-generation cortical suspensory fixation devices with adjustable graft loop length. The patients were followed-up with clinical examination, International Knee Documentation Committee (IKDC) scoring system and KT-1000 arthrometer with a minimum 12 month follow-up duration. In the post-operative a radiographic evaluation of the positioning of the femoral half-tunnel was performed.

**Results** We experienced an intra-operative rupture of a retrograde drill, and a traumatic failure. Normal ROM was observed in all patients at follow-up. Significant improvement was seen on Lachman test and pivot-shift test between pre-operative and last follow-up. KT-1000 side-to-side difference averaged 0.7 ± 0.2 mm. Average IKDC subjective score at follow-up was 95.7 ± 3.1, IKDC objective score was normal in 24 cases (A) and nearly normal in 8 cases (B). The centre of the femoral footprint was at 25.7 ± 2.9 % along the Blumensaat line and to 28.9 ± 1.5 % of the condylar height. All patients resumed sport activity at pre-injury level.

**Discussion** The use of sockets instead of tunnels associated to the use of second generation cortical suspensory devices permit to reduce the bone loss and guarantee the best bone-graft contact enhancing the ligamentization process of the graft. The use of one single tendon triplicated or quadruplicated represent an optimal solution for the knee biomechanics.

**Conclusions** The described technique showed to be minimally invasive and effective in restoring knee stability.

### Anterior cruciate ligament lesions: comparison between 4 different reconstruction techniques

M. Baldassarri*, R. Buda, G. Pagliazzi, A. Ruffilli, A. Olivieri, E. Ferranti Calderoni, S. Giannini

Istituto Ortopedico Rizzoli (Bologna, IT)

**Introduction** Anterior cruciate ligament (ACL) reconstruction with hamstrings is a common procedure. Many techniques and different fixation devices have been described in literature. The aim of this study was to retrospectively review the results obtained among 4 different ACL reconstruction techniques using hamstrings.

**Materials and methods** Eighty patients (mean age 27.5 ± 5.2 years) who underwent ACL reconstruction were evaluated at a mean follow-up of 26 ± 5 months. Twenty patients underwent an ACL reconstruction using the "Over the top" technique (group 1), 20 underwent the trans-tibial femoral half-tunnel drilling (group 2), 20 underwent antero-medial femoral half-tunnel drilling (group 3) and 20 underwent the all-Inside technique (group 4). The 4 groups were homogeneous in sex, age, time elapsed from injury to surgery, meniscus or cartilage associated lesions and follow-up. Patients were evaluated with objective and subjective IKDC score and Tegner Activity Scale.

**Results** No intra-operative complications and failure were experienced. Subjective IKDC score obtained in the group 1 was significantly lower with respect to the group 4 (88.3 ± 11.1 *vs* 97.1 ± 1.4, $p = 0.013$) and to the groups 2 and 3, even if no statistically significant relationship was evident (93.9 ± 5.8 and 93.1 ± 1.4 respectively, $p = 0.065$). The objective IKDC score was normal (A) in 85 % of patients in the group 1, in 90 % of patients in the groups 2 and 3 and in 95 % of patients in the group 4. Tegner score showed no statistically significant differences among all the four groups.

**Discussion** The non anatomic reconstruction with "Over the top" technique showed the worst clinical results and a higher incidence of rotational instability. Trans-tibial and antero-medial femoral half-tunnel drilling showed similar clinical results, but incapacity to reproduce the original femoral ACL footprint with the trans-tibial drilling, and the technically demanding step of the antero-medial femoral tunnel drilling, remains the major drawbacks. The all-inside technique showed to be minimally invasive and effective in restoring knee stability, with the better clinical outcomes reported in this series.

**Conclusions** Anatomic reconstruction techniques provide a better restoration of knee kinematics compared with the over the top technique. The all-inside technique is more anatomic than the trans-tibial technique, and showed to be simpler with respect to the antero-medial technique.

### Anterior cruciate ligament reconstruction and return to sport activity: postural control as the key to success

F. Franceschi*[1], R. Papalia[1], A. Tecame[1], S. Vasta[1], N. Maffulli[2], V. Denaro[1]

[1]Università Campus Bio-Medico (Rome, IT);
[2]Centre for Sports and Exercise Medicine, Barts and The London School of Medicine and Dentistry (London, UK)

**Introduction** The risk for re-tear following anterior cruciate ligament (ACL) reconstruction is influenced by several hormonal, neuromuscular, biomechanical and anatomic factors. One of the most important negative prognostic factors which markedly increase the risk for re-tear of the ACL is the presence of high knee abduction moment (KAM) which can be measured immediately after a vertical jump landing on both feet. In this study we evaluate the values for KAM in two groups of patients who underwent ACL reconstruction followed by a specific rehabilitation protocol focusing on recovery of muscular strength, proprioception and joint stability.

**Materials and methods** Since November 2011 we conducted a prospective investigation on two groups of 20 female professional athletes (the sample was uniform for age, body weight, height, sport practiced and time to surgery) with clinical and radiological evidence of ACL tear. One group of patients underwent reconstruction using hamstring tendon graft, while for the second group a patellar tendon graft was used. Post-operative rehabilitation was identical for both groups. Clinical outcomes were evaluated pre-operatively, at 3 months and at 6 months postoperatively by measuring clinical scores (IKDC and Lysholm scores) and by performing functional test for stability (single leg hop, time hop, crossover triple hop, KAM test).

**Results** All patients showed statistically significant clinical improvements in when compared to preoperative values ($p < 0.0001$). No intergroup significant difference was observed in all clinical scores and functional tests with the exception of KAM test. The group treated undergoing reconstruction with hamstring tendon graft had values for KAM exceeding the normal range (1°–3°) while in the other group values were within this range. This difference was statistically significant ($p < 0.0001$).

**Discussion** ACL reconstruction using grafts obtained harvesting hamstring muscles was recognized as the gold-standard technique in women since it's less invasive and provide better aesthetic results; however women showing high KAM values in their healthy knee with high functional demand (elite athletes) could not benefit from this approach because of its higher risk for re-tear in this population.

**Conclusions** ACL reconstructive surgery using patellar tendon graft, in association with a particular rehabilitation protocol centred on strength, proprioception and stability restoration can produce satisfactory values for KAM within the physiological range.

*C38–KNEE-ACL II*

### Two-year follow-up of clinical outcome in all inside ACL reconstruction

A. Quaglia*, C. Bait, M. Cervellin, E. Prospero, A. Redaelli, P. Volpi

Humanitas Clinical and Research Center (Rozzano, IT)

**Introduction** One of the most recent introduction in anterior cruciate ligament (ACL) reconstruction is the all inside technique. One of the main features of this procedure is the construction of half femoral and tibial tunnel and preservation of the anterior tibial cortex. The aim of our study was to describe the clinical results of patients treated with all-inside technique for ACL reconstruction after a two years follow-up. We also assessed the clinical results of a second group of patients treated with the conventional technique of single-bundle ACL reconstruction with semitendinosus and gracilis (ST-G). Study design is case series.

**Materials and methods** We performed ACL reconstruction in 40 patients that were divided in 2 groups of 20 patients each. The patients of one group were treated with transtibial all-inside technique with only ST. The second group was subjected to ACL reconstruction with the traditional single-bundle transtibial technique using ST-G. We recorded clinical data at a mean follow-up of 24 months using the IKDC, Vas, Lysholm scale and Tegner score.

**Results** The VAS score for the group of traditional technique was 84.6 ± 12.6, while the score for the all inside group was 81.6 ± 13.1 with a not statistically significant difference between the two groups. As far as the Lysholm scale in the group of traditional ACL reconstruction we got 35 % of “good results” (7 patients) and 65 % of “excellent results” (13 patients). In the all inside we got results “sufficient” for 4 patients (20 %), “good results” for 7 patients (35 %) and “excellent results” for 9 patients (45 %) (ns). The median Tegner score was 6.5 (2–10) for traditional reconstruction group and 6 (1–9) for the group all inside (ns). In relation to IKDC, 50 % of patients operated with standard technique were classified as class A, 45 % as class B, 5 % were included in class C. As for all inside group, 55 % were classified in class A, 40 % in class B and 5 % in class C. No patients were classified in class D group.

**Discussion** The results of our study seem to suggest that, concerning return to sport and recovery of joint function, the all inside technique for ACL reconstruction shows similar results compared to the traditional technique.

**Conclusions** The all inside technique is a viable option in the hands of an experienced surgeon which allows to obtain benefits regarding to tibial cortex preservation and thus the possible association of this technique with high tibial tibial osteotomies.

### Double bundle ACL anatomic reconstruction technique with no bone and joint hardware

D. Comba, C. Pesce*, E. Balboni, V. Casale

(Turin, IT)

**Introduction** According to the most recent literature, the anatomic double bundle techniques for ACL reconstruction seems to provide a better control of rotational stability of the knee. Most of the previously described surgical techniques in anatomic ACL reconstructions include various fixation systems with intra-tunnel hardware. An autologous graft constructed with semi-tendinous and gracilis tendons and a ToggleLoc Femoral Fixation device (ZipLoop Technology, Biomet, Inc.) can be used in a double-bundle ACL Reconstruction without any additional intra-bone or joint fixation. Aim of this prospective randomized study was to evaluate if the absence of hardware (fixation devices) inside the bone tunnels has any influence on the clinical results of anatomic ACL reconstructions. We hypothesized that the presence of an intra-tunnel hardware would affect on osseointegration of the tendon graft and could influence the final result of the reconstruction.

**Materials and methods** Fifteen patients (group A) with isolated ACL rupture, were operated at our institution with this original ACL reconstruction technique and clinically evaluated at minimum 12 months follow-up. The results (IKDC evaluation form, Lysholm score, Tegner scale and KT-1000 measures) were compared to those obtained with a control group (group B) of 15 patients operated with conventional anatomic double bundle ACL reconstructions using intra-tunnel hardware (Bio-Intrafix, DePuy, Inc) tibial fixation.

**Results** All outcomes of the 2 groups were significantly improved compared to the preoperative evaluation. No significant differences between the 2 groups were found for Lachman test, KT-1000 measurements and pivot shift test ($p = 0.37$). IKDC score were no significantly different ($p = 0.25$), while Tegner scale and Lysholm score revealed higher values ($p < 0.048$) for the group A (no hardware inside the bone tunnels).

**Discussion** Complexity of the surgical procedure (time consuming) and presence of large amount of intra-bone hardware are common in the anatomic ACL reconstruction. This no hardware in bone technique seems to provide an optimal knee stabilization and reduce costs and surgical time.

**Conclusions** The absence of hardware inside the bone tunnels is not clinically relevant. Further investigations on functional differences between the 2 groups, regarding post-operative pain and return to sport activities would be necessary to complete this study.

### Biomechanics of the medial patellofemoral ligament: viscoelastic behaviour

G. Criscenti*[1], G. Placella[2], E. Sebastiani[2], M. Tei[2], A. Speziali[2], G. Potalivo[3], G. Cerulli[4]

[1]Let People Move Research Institute, Bioengineering Division, Centro d’Ateneo “E. Piaggio”, University of Pisa (Arezzo-Pisa, IT);
[2]Università degli Studi di Perugia, Clinica Ortopedica e Traumatologica (Perugia, IT);
[3]Let People Move Research Institute, Nicola’s Foundation ONLUS (Arezzo, IT);
[4]Università degli Studi di Perugia, Nicola’s Foundation ONLUS, Let People Move Research Institute, International Orthopaedic and Traumatologic Institute (Perugia-Arezzo, IT)

**Introduction** The MPFL is the primary passive stabilizer that resists lateral translation of the patella. The time- and history-dependent viscoelastic behaviour of this ligament depends from the complex interaction among collagen, elastin, proteoglycans and water and it’s usually described by the quasi-linear viscoelastic theory (QLV) proposed by Fung. The objective of this study is the description of viscoelastic properties of the medial patellofemoral ligament.

**Materials and methods** In this work, we used an alternative approach whereby the QLV theory can be applied to experiments which utilize a slow strain rate in order to avoid experimental error such as overshoot and vibration. Ligament behaviour can be described using five material constants: A and B describe the instantaneous elastic response; C, $\tau1$ and $\tau2$ describe the reduced relaxation function. Fifteen human MPFLs were tested to evaluate the time and history-dependent viscoelastic behaviour. The specimens were fixed with sandpaper and cyanoacrylate in a standard clamp of an Instron

5965 materials testing machine. In order to reduce tissue hysteresis, the sample was preconditioned by a series of ten cycles until the strain of 3 % with a strain rate of 0.1 %/s, and then a stress relaxation test was performed by elongating the MPFL to 6 % strain and held for 60 min. Strain at the mid substance was measured using Vicon optical system to track four markers placed on the ligament.
**Results** In order to obtain the best fitting of the experimental data, the parameters were evaluated using the Levenberg–Marquardt algorithm. The obtained values of the parameters A and B are 1.21 MPa (SD 0.96) and 26.03 (SD 4.16) (dimensionless) respectively and describe the instantaneous elastic response. The value of the parameter C is 0.11 (SD 0.02) (dimensionless) while the relaxation times $\tau 1$ and $\tau 2$ measured 6.32 s (SD 1.76) and 903.47 s (SD 504.73) respectively.
**Discussion** The obtained results adequately describe the viscoelastic behaviour of the ligament emphasizing the initial linear elastic contribution of the material and the subsequent relaxation.
**Conclusions** The QLV theory describe with a good approximation the viscoelastic properties of MPFL. The study of these properties is fundamental to understand the MPFL's contribution as stabilizer and for the selection of the correct methods of repair and reconstruction.

### Biomechanics of the medial patellofemoral ligament: tensile behaviour

M. Tei*[1], G. Criscenti[2], E. Sebastiani[1], G. Placella[1], A. Speziali[1], G. Potalivo[3], G. Cerulli[4]

[1]Clinica Ortopedica e Traumatologica, Università degli Studi di Perugia, (Perugia, IT);
[2]Let People Move Research Institute, Bioengineering Division, Centro d'Ateneo "E. Piaggio", University of Pisa (Arezzo-Pisa, IT);
[3]Let People Move Research Institute, Nicola's Foundation ONLUS (Arezzo, IT);
[4]Nicola's Foundation ONLUS, Let People Move Research Institute, International Orthopaedic and Traumatologic Institute, Università degli Studi di Perugia (Perugia-Arezzo, IT)

**Introduction** The MPFL is the primary static stabilizer of the patellofemoral joint, tensions the patella during knee flection and is commonly injured when lateral patellar dislocation occurs. The objectives of this work are the description of mechanical properties of the isolated tissue and the structural properties of femur-MPFL-patella complex (FMPC).
**Materials and methods** The mechanical properties of human MPFL were evaluated via a uniaxial tensile tests of isolated ligament and femur-MPFL-patella complex in anatomical position. In this study, 15 isolated ligaments and 15 FMPC were tested. The isolated ligament was fixed with sandpaper and cyanoacrylate in a standard clamp of an Instron 5965 materials testing machine. Four markers were used to evaluate the displacement with Vicon optical system. In order to reduce tissue hysteresis, the isolated ligaments were preconditioned by a series of ten cycles until reaching the strain of 3 % with a strain rate of 0.1 %/s and then extended at 0.3 %/s until failure. In order to evaluate the structural properties of the FMP complex, the femur was fixed in a custom clamps and was internally rotated so that the line of the posterior femoral condyle was 37° ± 2° to the horizontal and the patella was fixed in a custom clamp with bone cement. The FMP complex were preconditioned with 10 cycles of elongation between 0 and 2 mm and then extended at 10 mm/min until failure.
**Results** The ultimate stress of the isolated ligament was 16 MPa (SD 11), the ultimate strain was 24.3 % (SD 6.8) and the Young Modulus was 116 MPa (SD 95). The ultimate load of the FMPC was 145 N (SD 68), the ultimate elongation was 6.4 mm (SD 1.6) and the linear stiffness was 36.3 N/mm (SD 14.1).
**Discussion** The results obtained from the tensile tests are similar to the ones present in scientific literature and show that the MPFL is the primary static stabilizer of the patellofemoral joint.
**Conclusions** The evaluation of the tensile mechanical/structural behaviour of the MPFL is fundamental to understand its contribution as a stabilizer and for the selection of the methods for its repair and reconstruction.

### The loss of extension test (LOE test): a new clinical sign for the anterior cruciate ligament insufficient knee

G. Piu*, F. Caputo, M. Sanna, C. Sanna, M. Salvi

Unità di Chirurgia Protesica e dello Sport-Gruppo Kinetika (Cagliari, IT)

**Introduction** This prospective study was created to evaluate the reliability of a new clinical test, which we termed the loss of extension test (LOE test). The LOE test investigates the loss of the normal maximum passive extension (MPE) of the knee affected by anterior cruciate ligament tear, as compared to the healthy knee.
**Materials and methods** The study was divided into two consecutive parts. Part 1 was designed to assess the normal MPE side-to-side difference in a healthy population. In Part 1,100 healthy adults were enrolled. Part 2 was designed to evaluate the LOE test reliability in injured knees. In Part 2 we included 196 selected patients.
**Results** Part 1. The average MPE side-to-side difference in the healthy population was not statistically significant. Part 2. The overall average MPE side-to-side difference of the injured group was 10.1 mm ± 14.1 (min −20; max 60) and was not statistically significant. An anterior cruciate ligament tear was found in 121 knees out of 196 patients. The average MPE side-to-side difference in the ACL insufficient group was 16.9 mm ± 13.4 (min −20; max 60) and was statistically significant ($p < 0.0001$). The LOE test accuracy was 83.7 %, specificity was 93.3 %, sensitivity was 77.7 %, positive predictive value was 95 % and negative predictive value was 72.2 %.
**Discussion** The reliability of the LOE test is comparable to that reported in the literature for the Lachman test and dynamic tests.
**Conclusions** The LOE test could represent a useful tool for the diagnosis of the anterior cruciate ligament insufficient knee.

### *C39–KNEE-MISCELLANEOUS II*

### Reconstruction of the medial patellofemoral ligament: a biomechanical comparison of two techniques

G. Placella*[1], G. Criscenti[2], M. Tei[1], A. Speziali[1], E. Sebastiani[1], G. Cerulli[3]

[1]Clinica Ortopedica e Traumatologica, Università degli Studi di Perugia (Perugia, IT);
[2] Bioengineering Division, Let People Move Research Institute; Centro d'Ateneo "E. Piaggio", University of Pisa (Arezzo-Pisa, IT);
[3]Nicola's Foundation ONLUS, Let People Move Research Institute, International Orthopaedic and Traumatologic Institute, Università degli Studi di Perugia (Perugia-Arezzo, IT)

**Introduction** In recent years, the scientific community has been focused its attention on the medial patellofemoral ligament as patellar stabilizer and numerous surgical techniques have been developed, both anatomical and not-anatomical, for the reconstruction of this ligament. The purpose of this study is to compare the through tunnel technique, which has achieved the best biomechanical results in literature, with a technique developed by prof. Cerulli that respects the anatomy of the ligament using gracilis and semitendinosus tendon as a graft.

**Materials and methods** In this study, 18 fresh frozen anatomic specimens randomly divided into two groups were tested. The specimens of the first group were reconstructed with the through tunnel technique using semitendinosus or gracilis tendons. In this case, a transverse tunnel on the patella and one on the femoral condyles were made to attach the ends of the graft with absorbable interference screws. In the second group a semicircular tunnel on the medial side of the patella were made. The graft was passed and anchored to the femur with an absorbable interference screw. Subsequently the samples were dissected, the femur was fixed with a custom-made clamp and was rotated internally so that the line of posterior femoral condyle was $37° \pm 2°$ to the horizontal. The patella was fixed with bone cement in a custom clamp and the samples were preconditioned with 10 cycles of elongation between 0 and 2 mm and then extended at 10 mm/min until failure.

**Results** The ultimate load obtained with the through tunnel technique was 171 N (SD 51) while the one obtained with the technique with the patellar semicircular tunnel on the medial side was 213 N (SD 90).

**Discussion** The obtained results are not significantly different ($p > 0.05$) from the ones obtained with the native MPFL. The advantage of the technique with semicircular tunnel is the use of a single screw, a more equal distribution of stress on the patella and the relative simplicity of the surgical procedure.

**Conclusions** The technique developed by prof. Cerulli for reconstruction of the medial patellofemoral ligament allows a better stabilization of the tendon graft, a better distribution of the loads on the patella and respects the anatomy of the native ligament.

### Postero-medial tibial plateau nonunion

M. Massobrio*, G. Pellicanò, P. Albanese, G. Antonietti, U. Bianco, G. Corsi

Dipartimento di Scienze Anatomiche Istologiche Medico-Legali e dell'Apparato Locomotore, Università "La Sapienza" (Rome, IT)

**Introduction** The postero-medial tibial plateau nonunion is the less known cause of post-traumatic knee deformity and it leads to precocious knee degeneration. There are no studies in literature concerning this location and its treatment.

**Materials and methods** We studied the clinical features and the treatment's options in a group of 21 patients, mean age 44.6 years old (19–77), who came to our out-patient clinic and later diagnosed with postero-medial tibial plateau nonunion. Diagnosis was made through X-rays and CT scans performed from 6 to 14 months after the fracture synthesis. All patients were treated surgically using the anterior Insall approach extended in the postero-medial region. We performed the synthesis removal, submeniscal arthrotomy, nonunion site curettage and resection, reduction, and new synthesis (with plate and screws in 17 cases, with external fixation in 4 cases). In 9 patients we performed a bone autograft from the iliac crest. Patients started articular mobilization from the 1st postoperative day. Full weight bearing was allowed from 60 to 90 postoperative days. Follow-up is from 1 to 5 years.

**Results** The clinical assessment at the end of the treatment showed an increase in the Lysholm-II Knee Score from a mean score of 34, to 72. All nonunions were reduced anatomically. In all patients knee extension was complete, knee flexion was more than 90° in 14 patients, between 60° and 90° in 7 patients.

**Discussion** We identified similar radiological findings in all patients. The postero-medial region of the tibial plateau was affected by nonunion because of a missed radiological identification, or of the fragment's insufficient reduction and synthesis. The best results were obtained in the patients treated precociously, without waiting for the nonunion's radiological aspects to become obvious. The knee CT scan is essential in patients who underwent synthesis for a tibial plateau fracture if they present with pain or a varus knee deformity.

**Conclusions** Postero-medial tibial plateau nonunion must be identified in order to perform a treatment that will lead to knee ROM reactivation, functional recovery, and to avoid precocious joint degeneration.

### Nanohydroxyapatite promotes healing process in open wedge high tibial osteotomy: a CT study

P. di Sette*, A. Ferretti, R. Iorio, F. Conteduca, G. Argento, M. Pagnottelli, D. Mazza, A. Redler, G. Bolle

Ospedale Sant'Andrea, Università "La Sapienza", (Rome, IT)

**Introduction** Open wedge high tibial osteotomy (OWHTO) is a common procedure for the treatment of symptomatic varus misaligned knees. Healing of bone graft used to fill the osteotomy gap is an important issue. The aim of this paper is to evaluate The effectiveness of adding nano-hydroxyapatite (nHA) to heterologous bone graft by measuring bone density of tibial osteotomy gap.

**Materials and methods** Twenty-six patients (26 knees) were operated by open wedge high tibial osteotomy (OWHTO) and randomly divided in two groups: group A, where the osteotomy gap was filled with only heterologous bone graft; group B, where the osteotomy gap was filled with heterologous bone graft and nano-hydroxyapatite (nHA). All patients undergone CT examination within 1 week after operation (time 0), after 2 months (time 1) and 12 months (time 2). CT volume acquired Hounsfield units (HU) were calculated and mean values of bone density on three-planes measured.

**Results** At time 0, the mineral density of group B appears significantly higher compared with group A (223.8 + 83.1 HU; $p < 0.05$). At time 1, the mineral density of group B decreases while in group A it remains unchanged (191.3 + 75.7 HU; $p < 0.05$). At time 2, group B mineral density further decreases reaching the values close to the mineral density of normal bone (224.4 + 24.2 HU). In contrast with this, in group A the mineral density increases, (425.2 + 61.6 HU; $p < 0.05$).

**Discussion** This study focuses on the process of osteointegration of bone grafts and the possible benefits induced by nano-molecular hydroxyapatite treatment. In our study we evaluated, with CT scans, the degree of osseointegration of the bone grafts we implanted. We did not find in literature any study on quantitative assessments made with CT scans, which has evaluated the level of osseointegration of bone grafts used after OWHTO.

**Conclusions** To our knowledge this is the first study to evaluate bone density after OWHTO with multislice CT method. The data of the present study suggest a better osteointegration of the heterologous graft when the nano-hydroxyapatite is added.

### Combined arthroscopic treatment of tibial plateau fractures associated with tibial intercondylar eminence avulsion

A. Parma*, R. Buda, A. Ruffilli, E. Ferranti, S. Natali, M. Baldassarri, S. Giannini

Istituto Ortopedico Rizzoli (Bologna, IT)

**Introduction** The tibial plateau fractures associated with avulsion of the intercondylar eminence are an uncommon occurrence. An isolated lesion of the spine is relatively frequent in children, while in adults usually occurs in association with other joint injuries. The aim of this study was to describe a technique for minimally invasive arthroscopic treatment of these lesions and evaluate the long-term results.
**Materials and methods** Twenty-five patients, mean age of 22.2 years were treated with arthroscopic technique from 2000 to 2010. Fractures of the tibial plateau were divided according to the classification of Schatzker, while the tibial eminence lesions, according to Meyers and McKeever. Pre-operative evaluation was clinical, radiographic and TC scans. The percutaneous fixation of the fracture was possible in all cases in view of the type of fracture (Schatzker type I, II, IV). All subjects were treated with: arthroscopic reduction of tibial plateau fracture and synthesis, reduction and fixation of tibial eminence by ligamentotaxis, using two different techniques (single tunnel or double tibial tunnel). Patients were reassessed an average of 90 months (range 34–139 months) by clinical examination, IKDC, KT-1000 arthrometric evaluation and X-ray (under weight-bearing).
**Results** The mean subjective IKDC was 83.3, while in objective IKDC 44 % of the patients were classified as grade A (40 % grade B, 12 % grade C, 4 % grade D). The degree of laxity measured KT-1000 from one extreme to the other was less than 2 mm in all cases. Nine patients, with Schatzker fracture type II presented a residual depression of the tibial plateau outside (average 0.7 mm range 0–4 mm). There was no significant difference between the two techniques of fixation of tibial eminence fracture.
**Discussion** The use of an arthroscopic technique makes it less surgical trauma and allows you to treat associated intra-articular injuries (meniscal, osteochondral gaps). The benefits include: less devascularization of fragments, reduction of postoperative pain and swelling, earlier recovery of motion with less rigidity and less risk of infection. The treatment of the lesions associated with the tibial spine allows to restore sufficient functionality ligamentous thanks to the correct tensioning of the anterior cruciate ligament.
**Conclusions** The combined arthroscopic treatment of fractures of the tibial plateau associated with tibial intercondylar eminence avulsion has proven able to provide a sufficiently effective synthesis of the fracture and, simultaneously, to ensure a satisfactory stability of the knee in the long term.

### *CS01–RESIDENTS' ORAL COMMUNICATIONS 1*

### Biomechanical evaluation of magnesium-based interference screws for the ACL reconstruction

A. Speziali*[1], M.M. Tei[1], E.K. Kim[2], N. Sasaki[2], P.J. McMahon[2], L.-Y. Woo Savio[2], G. Cerulli[1]

[1]Let People Move Research Institute (Perugia-Arezzo, IT);
[2]Department of Bioengineering, University of Pittsburgh (Pittsburgh, USA)

**Introduction** We have developed a novel magnesium (Mg)-based metallic interference screw for ACL reconstruction. The advantages of this device include bioabsorbability, osteoconductivity, plus minimal MRI distortion and its degradation can be controlled. We hypothesize that the Mg-based screws could achieve the same level of graft fixation than that of the titanium (Ti)-screws.
**Materials and methods** Five pairs of skeletally mature goat knees underwent ACL reconstruction using bone-patellar tendon-bone (BPTB) autograft. At the femur, the graft fixation was randomized with Mg-based screw or Ti-screw. Afterward, the femur-ACL-tibia-complex (FATC) was dissected and tensile tested by mean an Instron tensile testing machine (Model-4502) with a protocol including three 100 cycles loading and unloading tests between 20–70 N (C1), 20–105 N (C2), and then 20–70 N (C3). After one hour of recovery graft slippage between each cyclic test was measured. Subsequently, the FATCs were loaded to failure, and linear stiffness and ultimate load were obtained from the load-elongation curve.
**Results** The graft slippage following the cyclic tests (C1–C3) was 0.8 ± 0.6 mm in the Mg-based screws compared to 0.8 ± 0.3 mm in the Ti-screws, and there was no statistical significant difference between the two fixation devices (paired Student's $t$ test, $p = 0.9$). Also, no statistical significant difference was found in linear stiffness (50.7 ± 7.5 N/mm for the Mg-based screws and 51.3 ± 13.1 N/mm for the Ti-screws, $p = 0.9$). For the ultimate load there was a statistical difference (414.8 ± 85.7 N and 467.7 ± 23.5 N, respectively, $p = 0.3$).
**Discussion** The osteoconductivity of Mg-alloy screw as well as its programmed degradability offer advantages for graft healing within the bone tunnels. The ductility of the metallic screws also could overcome complications of the polymer screws including their breakage.
**Conclusions** Confirming our hypothesis, we have demonstrated that the novel Mg-based metallic screw could achieve a comparable level of fixation and better ultimate load of a BPTB graft as that of the traditional Ti-alloy screws.

### The role of graft tensioning for the knee stability during anterior cruciate ligament reconstruction: systematic review of level I and II therapeutic studies

A. Speziali*[1], M.M. Tei[1], G. Placella[1], E. Sebastiani[1], F. Manfreda[1], A. Amanti[1], P. Antinolfi[2], G. Cerulli[1]

[1]Let People Move Research Institute (Perugia-Arezzo, IT);
[2]Clinica Ortopedica e Traumatologica (Perugia, IT)

**Introduction** In USA, more than 100,000 ACL reconstructions per year are performed. However, wide differences can be reported between the graft tensioning techniques. The purpose of this systematic review is that to assess the role of graft tensioning for the stability of the knee following ACL reconstruction at the early follow-up.
**Materials and methods** Two reviewers (AS and MT) performed a systematic search using three electronic database, Cochrane Controlled Trials register, Medline and Embase, on the primary ACL reconstruction using quadrupled semitendinosus and gracilis (QSTG) and bone patellar tendon bone (BPTB) in randomized controlled trials of level I and II of evidence published from January 2001 to December 2011. Therapeutic studies included a minimum of 12 months follow-up and at least an arthrometric evaluation of the anterior-posterior tibial translation (APTT).
**Results** Twenty studies were included for the final examination. Overall, 1697 patients underwent primary ACL reconstruction in 73.8 % using QSTG and in 26.2 % using BPTB. The mean age of the patients was 27.6 years. APTT was 1.6 ± 0.9 mm, 2.0 ± 0.8 mm and 2.2 ± 0.2 mm for manual graft tensioning, for a tensioning between 40 to 80 N and for a graft tensioning between 90 to 150 N, respectively.
**Discussion** Improper graft tensioning for ACL reconstruction is a key-point that can lead to overtensioning and excessive tibio-femoral constraint or it can lead to undertensioning and subsequent knee instability. Clinical scientific evidence of a reliable method for graft tensioning is still lacking.
**Conclusions** We had reported that the manual graft tensioning achieved a better knee stability at the early follow-up. However, manual tensioning could be affected by intra-operative variability. From the other side, there is no scientific evidence on the role of graft tensioning on the knee stability during ACL reconstruction.

### How post-operative complications after spinal surgery affect the final outcome in Rett syndrome patients. Long-term follow-up

L. Labianca, C. Calderaro*, V. Di Sanzo, P. Rota, A. Carducci, F. Turturro, A. Montanaro, M. Guidi, R. Hoedemaeker, A. Ferretti

Azienda Ospedaliera Sant'Andrea, Università "La Sapienza" (Rome, IT)

**Introduction** Rett syndrome (RS) is associated with a neurological form of C-shaped thoracolumbar kyphoscoliosis. The aim of this study was to evaluate pre-operative curve severity, intra-operative data (bleeding and surgery-time), neuromuscular impairment and prevalence of postoperative complications in order to clarify how they could affect the preoperative decision-making process, regarding time of surgery and postoperative care.
**Materials and methods** From 1989 to 2012 twenty-one patients with Rett syndrome and neuromuscular scoliosis were treated by the same surgeons, with posterior spinal fusion and Luque or Hybrid Instrumentation. Preoperative medical conditions and postoperative complications were recorded. All events that protracted the hospital stay or the healing period, required an additional surgical procedure, or affected final result were considered major complications. Rate of complications was analyzed according to the severity of the curve, intra-operative bleeding and recovery stay in postoperative intensive care unit (PICU) with Chi square test.
**Results** At surgery mean age was 12.4 years (range 6–17) and mean postoperative follow-up was 12.9 years (range 2–24). Pre-operative mean value of scoliosis was 80° (range 47°–112°). Post-operative value 33° (range 20°–50°). Mean preoperative pelvic imbalance was 20° (3°–32°), post-operative 11° (2°–16°). Mean recovery-stay in PICU was 12.3 days (range, 2–61 days). GMFCS reported 7 grade V, 6 grade IV, 8 grade III. We recorded 13 total complications (61 %) in 10 of 21 patients (47.6 %): 8 major complications in 6 patients (2 cases of deep infection with instrumentation removing and 6 cases of stay in paediatric PICU due to respiratory impairment more than 7 days, in one case cardiac arrest caused death after 60 days in PICU) and 5 minor in 4 patients (1 case of late mechanical failure, without loss of correction and 4 cases due to respiratory impairment; recovery time in PICU $> 4$ days and $<7$ days).
**Discussion** Scoliosis is a frequent orthopaedic problem in Rett syndrome. Complications rate is still remarkable in patients that underwent spinal surgery and is still to clarify how could affect the final result. In our series the prevalence of deep infection is similar to other neuromuscular scoliosis treated in our centre (9.5 % *vs.* 9.1 %), and comparable with data in literature (10–25 %).
**Conclusions** Despite the high complications rate (61 %), it seems not to be strictly related with the failure of the procedure so at final follow-up the success rate is high. Moreover it is mandatory to manage this kind of patients with a dedicated paediatric PICU high skilled in neuromuscular disorders and respiratory care.

### Use of neridronic acid in the treatment of osteogenesis imperfecta type I. Four-year results report

C. Mela[1], L. Saturno[2], F. Milia[1], P. Lisai[1]

[1]Clinica Ortopedica Sassari (Sassari, IT);
[2]Ospedale SS Annunziata (Sassari, IT)

**Introduction** Osteogenesis imperfecta gathers a wide range of genetic disorders that involves with different degrees of severity type 1 collagen, which is commonly present in bones, skin, tendons, scar tissue. The defect may be qualitative or quantitative, with large variety of phenotypes. It leads to a lower bone density, higher risk of fracture, even for low energy traumas, skeletal deformities, joint hyperlaxity, growth defects, dentinogenesis imperfecta, blue sclerae, hearing impairment. Authors report about efficacy of neridronic acid in increasing bone density and lowering fracture rate in patients with osteogenesis imperfecta.
**Materials and methods** This study has been conducted following 26 patients, 9 to 65 years old, in a 4-years' time between 2008 and 2012. One group has been treated with 75 mg of neridronic acid intravenously every three months; another group has been treated with 25 mg of neridronic acid intramuscular every three months as well. Blood tests and urine analysis were carried out to keep monitoring bone turnover, also computerized bone mineralometry and spine and femur X-rays were performed.
**Results** In a 4-years' time lowering of the bone turnover, increasing of bone density and a drop of fracture rate have been reported.
**Discussion** Neridronic acid acts on mevalonate pathway, inhibiting farnesyl-pyrophosphate-synthase enzyme. Interruption of this step leads to a quick osteoclast apoptosis. At the moment is the only bisphosphonate based drug licensed in Italy for the treatment of osteogenesis imperfecta. Efficacy in intravenous administration is not different from intramuscular administration and few side effects have been reported by the patients during therapy, which shows a good tolerability.
**Conclusions** Neridronic acid is effective to increase mineral bone density and to decrease fracture rate. At the moment is the first choice treatment in patients with osteogenesis imperfecta. This treatment has shown a double action, physical and psychological. On one hand in fact patients have a real benefit from the bisphosphonates effect on the bone tissue. On the other hand they gain self confidence and independence in everyday activities and it helps to deal with a disease that still affects life quality.

### *CS02–RESIDENTS' ORAL COMMUNICATIONS 2*

### Predicting factors in damage control orthopaedics

M. Marinelli, N. Giampaolini*, N. Orlando, L. de Palma

UNIVPM Scuola di Specializzazione in Ortopedia e Traumatologia (Ancona, IT)

**Introduction** Surgical orthopaedic timing in poly-traumatised patients is a debated issue. The definitions "early fracture fixation", "early total care" and "damage control surgery" have been used over the past years and there have been numerous attempts to identify the predicting factors of the poly-traumatised patients outcome and the elements that can guide the surgeon to choice the most appropriate surgical time.
**Materials and methods** All the poly-traumatised patients of orthopaedic interest that reached the Emergency Room of our hospital from 1/1/2008 to 31/12/2010 have been included in this study. For each patient orthopaedic surgical timing and clinical and laboratory findings collected at the Emergency Room have been considered. A statistical analysis was conducted to find if there is a significant correlation among these parameters and the mortality.
**Results** The percentage of patients that underwent orthopaedic surgery within 12 h from the trauma was 72 %, while the remaining 28 % was treated after this time threshold. None of the patients surgically treated died. The clinical and laboratory parameters that have a statistically significant impact on the mortality are: PA $> 90$ mmHg, thrombocytopenia, hematocrit $< 30$ % and haemoglobin concentration $< 10$ mg/dl.

**Discussion** Our analysis of orthopaedic surgical timing in this group of patients shows some findings that agree with the majority of the evidences described in the international literature. The parameters that have been demonstrated to predict poly-traumatised patients outcome should guide the surgical timing and should always be measured at the Emergency Room. In the group of patients observed, some elements of critical importance such body temperature and Glasgow coma scale have not been registered at the patient's access.
**Conclusions** Our data show that the clinical features and the laboratory findings, measured at the access to the primary health care of the poly-traumatised patients, that have a statistically significant correlation with the mortality, are blood pressure $< 90$ mmHg, platelets $< 150000/mm^3$, hematocrit $< 30$ % and haemoglobin $< 10$ g/dl. In our study we didn't find a relationship between mortality and orthopaedic surgical timing, heart rate and patient's age, these data are in contrast with the current literature.

### Total elbow replacement in non traumatic diseases: long term follow-up

M. Zanelli*, F. Pogliacomi, D. Aliani, F. Calderazzi, M. Corradi, F. Ceccarelli

Dipartimento di Scienze Chirurgiche, Clinica Ortopedica e Traumatologica, Università degli Studi di Parma (Parma, IT)

**Introduction** In the past, operative procedures such as arthrodesis, resection and resurfacing arthroplasty were the only way to improve pain, joint instability and stiffness associated with various pathologies of the elbow. In the late 1970s, total elbow replacement was developed with results which have progressively improved. The choice of the implant depends on the underlying pathology, on the degree of damage of the capsuloligamentous structure, and last but not least, on surgeon's preference.
**Materials and methods** Twenty patients affected by degenerative or inflammatory diseases were treated by semi-constrained total elbow replacement. All subjects, after a minimal follow-up of 5 years, were clinically assessed by the Mayo Elbow Performance Score (MEPS), by the DASH (Disabilities of the Arm, Shoulder and Hand) questionnaire before and after surgery and by radiological investigation in order to evaluate component positioning and to detect signs of prosthesis mobilisation.
**Results** Mean MEPS and DASH before surgery and at follow-up was respectively 57.8/55.7 and 87.5/26.1. Radiographic analysis showed that in 18 of the 19 cases no prosthesis loosening or failing were observed. In one patient, prosthetic aseptic mobilization was observed 17 months post-operatively, requiring implant revision. Another patient developed joint stiffness during flexion as consequence of impingement between coronoid process and anterior flange of the prosthesis; despite this complication the patient was satisfied of the surgical procedure. Post-operatively, one patients exhibited signs of superficial infection which resolved by targeted intravenous antibiotic administration and another ulnar nerve neuropraxia which spontaneously disappeared 6 months after surgery. One patient developed deep *Staphylococcus* infection. Radiographic investigation failed to show signs of mobilization and treatment consisted in targeted antibiotic administration associated with soft tissue surgical debridement.
**Discussion** The results of this study confirmed the validity of this procedure in these selected cases and showed a significant improvement of their elbow function and quality of life after surgery. A precise surgical technique is mandatory in order to prevent complications. Particular attention has to be given to preserve soft-tissues and in positioning and cementation of the prosthetic components.
**Conclusions** Total elbow replacement in patients affected by degenerative or inflammatory diseases can be considered a consolidated technique characterized by satisfactory long-term results.

### Treatment of idiopathic clubfoot: comparison between two methods

G. Piarulli*, D.E. Gallone, A.D. Cossio, M. Turati, G. Zatti

Azienda Ospedaliera San Gerardo (Monza, IT)

**Introduction** Clubfoot is a three-dimensional congenital deformity of foot which needs an immediate corrective treatment. The present study aims to compare two different treatment methods adopted in our institution between 1992 and 2008.
**Materials and methods** We compared retrospectively 60 children with congenital talipes equinovarus from grade 2 to grade 4 according to Dimeglio classification. Patients were divided in 2 groups: group A included 31 children (47 feet of which 13 of grade 2, 33 of grade 3 and 1 of grade 4), group B included 29 children (42 feet of which 13 of grade 2, 25 of grade 3 and 4 of grade 4). Method A consists in serial application of long-leg casts in gradual correction for 4 to 6 weeks starting from the second week of life followed by daily manipulation carried out by a skilled physiotherapist and by parents. Method B involves, in addition to casting and physiotherapy, functional taping and application of custom-made thermoplastic splint for 1 to 2 years to maintain the correction achieved with manipulations. Exclusion criteria were grade 1 deformities, as these were corrected with physiotherapy only, and non idiopathic deformities. Clinical outcome was evaluated using McKay's morphologic and functional score after a minimum four-year follow-up. We considered the use of Achilles tendon lengthening with posterior capsulotomy as failure of the non-surgical treatment.
**Results** The percentage of feet that required surgical treatment dropped from 95.7 % of method A to 16.3 % of method B ($p < 0.05$). We didn't observe any statistically significant difference in the grade of correction obtained.
**Discussion** In spite of comparable morphological and functional results, the introduction of method B drastically reduced the need for surgical treatment and hence potential related complications.
**Conclusions** In consideration of above mentioned findings we currently threat most of the patients with method B. A persistent commitment of parents and healthcare staff is nevertheless essential to achieve these results.

### Evaluation of cell viability in tenocytes from autografts and allografts in knee anterior cruciate ligament (ACL) reconstruction

A. Mancò*[1], S. D'Ascenzo[2], I. Giusti[3], A. Dal Mas[4], V. Dolo[3], V. Calvisi[1]

[1]Scuola di Specializzazione in Ortopedia e Traumatologia, direttore Prof. V. Calvisi, Università degli Studi dell'Aquila (L'Aquila, IT);
[2]Dipartimento di Medicina Clinica, Sanità Pubblica, Scienze della Vita e dell'Ambiente; Università degli Studi dell'Aquila (L'Aquila, IT);
[3]Dipartimento di Medicina Clinica, Sanità Pubblica, Scienze della Vita e dell'Ambiente; Scuola di Specializzazione in Patologia Clinica, direttore Prof.ssa V. Dolo, Università degli Studi dell'Aquila (L'Aquila, IT);

[4]UOC Anatomia Patologica Ospedale Civile "San Salvatore" (L'Aquila, IT)

**Introduction** In ACL reconstruction, which is often essential for restoring knee joint stability, one of key aspects is the biological quality of the graft, whether it is autologous or from a tissue bank: the latter, in recent years, had a great appeal, not only for patients, both for the absolute poor peri-operative morbidity and, mainly, for a boasted earlier return to sport activity. Taking into account these expectations our purpose was to assess the viability of the cells isolated from different types of tendon samples.
**Materials and methods** Tenocytes were isolated from 13 samples, 7 of them were autologous samples (gracilis and/or semitendinosus), 5 were from allograft (peroneal tendons) and 1 was Neo-ACL (revision after hamstring autograft); all these samples were minced and tenocytes migrated from such explants and adhered to Petri dishes forming a monolayer. Cells were grown in DMEM/HAM S F-12 supplemented with 10 % FCS and a mix of antibiotics until reaching full confluence, then detached using a trypsin-like enzyme and characterized by immunohistochemistry (by vimentin, a nonspecific marker classically used in characterization of tenocytes) and Western blotting (by Scleraxis, a specific marker currently used for these cells).
**Results** Autologous and Neo-ACL samples showed good cell viability, with achievement of full confluence in about 6–8 weeks. In allograft samples, instead, was not possible to observe any growth in the same interval. The cells, seeded on glass slides, were strongly positive for vimentin; moreover, in the cell extracts was possible to show the presence of Scleraxis, using specific antibody, proving that cells are effectively tenocytes.
**Discussion** To assess the biological quality of the samples used in surgical practice, we isolated and characterized the cellular component of tendons, tenocytes, using a technique that requires tendon mincing and following cultivation in a controlled environment. Samples showed good cell viability both in autologous and in Neo-ACL samples; on the contrary, in allograft samples was not possible to show cell growth.
**Conclusions** Scientific evidences show a substantial overlap of clinical results using autograft and/or allograft. Our simple but significant experience, since demonstrates the absence of viable cells in allograft samples, raises doubts on the ligamentization of the graft, although it doesn't exclude that they can work as a scaffold.

### Medial femoral neck fractures treated with cannulated screws and biophysical stimulation: clinical and radiological follow-up at 2 years

S. Mangano*, L. Costarella, S. Gioitta Iachino, F.R. Evola, V. Pavone, G. Sessa

Azienda Universitaria Ospedaliera Vittorio Emanuele-Policlinico (Catania, IT)

**Introduction** Medial femoral neck fracture represents 3 % of all fractures and regards, above all, elderly women, following on low-energy traumas. The gold standard of treatment is total hip arthroplasty; however, in stable or slightly displaced fractures (Garden type I and II) it is possible to opt for minimally invasive osteosynthesis with cannulated screws. The purpose of the following study is to evaluate, both clinically and through digitalized radiology, patients affected by medial femoral neck fracture stabilized with three cannulated screws, and treated for 3 months with biophysical stimulation with pulsed electromagnetic fields (PEMF).
**Materials and methods** The study group consisting of 45 patients operated within 24 h after the trauma (on average 6–10 h), between January 2011 and September 2012, was subdivided into 2 groups: those stimulated with PEMF for 8 h per day over 90 days (group 1), and those not stimulated (group 2). The first group was made up of 23 patients (14 men and 9 women, average age 61 years, range from 48–74 years) of which 10 with Garden fracture type I and 13 with type II who underwent biophysical stimulation from 3 days after the operation. The second group is made up of 22 patients (12 women and 10 men), average age 65 years (range from 42–89 years) of which 10 Garden type I and 12 type II. The clinical follow-up, using Harris hip score, and radiographic evaluation was carried out at 1, 3 and 6 months from the synthesis and subsequently at 15 months.
**Results** The osteonecrosis of the femoral head and the loss of alignment represented the end point after which hip replacement is performed. The average follow-up of 15 months (range 5–25) showed: 85 % success in the first group, complete healing within 90 days, and 5 cases of failure; in the second group the success percentage was 68 %, healing after 90 days, and 7 cases of failure.
**Discussion** As confirmed by literature, stimulation with PEMF, inhibiting reabsorbing processes and increasing bone formation, is shown to reduce time needed for bone healing, and prevent osteolysis around the screws. Moreover, it limits the inflammatory process and pain, and stimulates revascularization of the femoral head.
**Conclusions** The results at our disposal show that stimulation with PEMF accelerates healing of medial fractures of the femoral head treated with cannulated screws. The protocol we adopted, in light of the evidence, should be standardized in the therapeutic treatment of selected cases.

### Evaluation of static balance after reconstruction of ACL by means of specific software based on Microsoft Kinect

F. Del Prete*, F. Masia, F. Mela, P. Zedde, A. Melis, A.F. Manunta

UOC Ortopedia e Traumatologia (Sassari, IT)

**Introduction** Surgery of ACL lesions is one of the main topics especially concerning graft choices and methods of fixation. Regardless the type of graft and surgery technique, reconstruction of ACL has the aim of guaranteeing static and dynamic stability with an almost full range of motion.
**Materials and methods** This study shows clinical and instrumental assessment of 10 patients (3 women and 7 men) between 18 and 50 years old, who underwent surgery for ACL reconstruction between 2006 and 2012 (5 DTSG and 5 TR; in one case both ACL had been previously reconstructed with DTSG). The evaluation of patients before surgery showed a IKDC score between 30–35 %, while a score between 80–85 % in the post-operative after 7 months. The evaluation of articular stability of the knee was carried out by means of KT 2000 and a specific software for static balance developed by Area3 (Alghero) and Aqvatech Engineering (Turin). This software is based on Microsoft Kinect. The acquisition consisted of 3 trials for the left lower extremity and 3 trials for the right one. Each trial, 30 s lasting, was preceded by a preliminary phase of 10 s with the purpose of achieving a specific average-position as a standard for each patient.
**Results** The system showed some static and proprioceptive asymmetries varying from −3 and −81 % for the extremity undergone surgery in 7 cases out of 10. In two cases not important difference has been observed. In one case a benefit for the damaged extremity has been observed (surgery dating about two years earlier). In the only case in which both ACLs were reconstructed, a significant deficit for the extremity which has undergone surgery more recently has been observed. Due to the small number and type of distribution of the examined sample, it's not possible to highlight significant differences between the sexes.

**Discussion** The analysis of the outcomes shows asymmetries of static and dynamic type between the surgical and non-surgical extremities with a deficit of static type for the surgical knee in 70 % of cases. One case, in which both knees underwent ligament reconstruction, shows a larger deficit for the extremity which underwent surgery more recently.

**Conclusions** It's possible that different physio-pathological mechanisms, not yet analyzed, may lead to a decrease of such deficit in the long run. The latter could also be influenced by different factors related to patient response, type of surgery performed and post-operative rehabilitation.

### Total hip arthroplasty after acetabular fracture

A. Pantalone*, D. Vanni, A. Puzzo, M. Berloco, A. Barbati, B. Flacco, S. Sogari, V. Salini

Clinica Ortopedica e Traumatologica (Chieti, IT)

**Introduction** In the management of acetabular fractures, total hip arthroplasty (THA) plays an important role. This includes immediate total hip replacement for certain rare cases and late reconstruction following failed non-operative or operative treatment, leading to symptomatic arthritis, post-traumatic avascular necrosis of the femoral head, malunion or non-union.

**Materials and methods** A retrospective study was performed on 37 patients (27 men and 10 women with a range between 19 and 70 years) treated for pelvic ring and acetabulum fractures between January 2008 and May 2012, at Orthopaedic and Traumatology Department of Chieti. The fractures were classified according to Tile (pelvic ring) and Judet & Letournel (acetabulum). Actually, Total hip arthroplasty was performed for avascular necrosis of the femoral head in 2 cases.

**Results** After 2 years, the average Harris Hip Score improved from 37 points pre-operatively to 88 post-operatively. To date, no patient needed a revision surgery.

**Discussion** THA is a more severe intervention in patients with posttraumatic arthritis or avascular necrosis of the femoral head, especially if an internal fixation of the acetabulum was previously performed: the mean operating time was longer, there was a higher transfusion need, more patients needed bone grafting and there was a higher incidence of intra-operative hip instability. Preoperative factors including infection and nonanatomic restoration of hip centre would predict a poor outcome of THA after acetabular fracture. In order to achieve a high survivorship rate of hip prosthesis, optimizing the anatomy of the reconstructed hip center at reconstruction is paramount (Pascarella R. et al., Aggiorn Club Ital Osteosint 2012).

**Conclusions** The treatment of acetabular fractures is complicated and depends on numerous factors including age, functional status, fracture pattern, degree of displacement, associated injuries, and surgical experience. Indications for operative management of acetabular fracture are rapidly expanding and evolving. Our experience and results found in literature suggest that an open, anatomic reduction and internal fixation afford the best opportunity for joint preservation and minimize the risk of posttraumatic osteoarthrosis. The indications for total hip replacement after failed treatment of acetabular fractures could be classified into two groups: avascular necrosis of the femoral head and symptomatic post-traumatic or postoperative arthritis. Despite obvious challenges, advances in fracture management and cementless acetabular fixation in THA demonstrate improved results for post-traumatic arthritis and avascular necrosis of the femoral head following acetabular fracture (Bellabarba C. et al., J Bone Joint Surg 2001; Sermon A. et al., Injury 2008; Ranawat A. et al., J Arthroplasty 2009).

### *CS03–RESIDENTS' ORAL COMMUNICATIONS 3*

### Risser's plaster corset for the treatment of adolescent idiopathic scoliosis: is it still relevant today?

L. Zottarelli*, G.A. La Maida, B. Misaggi

Istituto Ortopedico Gaetano Pini (Milan, IT)

**Introduction** Plaster corset, according to Risser technique, has been for a long time a milestone of the conservative treatment of the adolescent idiopathic scoliosis. Primary outcomes are the correction or at least the arrest of the scoliotic curve and the hump's reduction. Usually, the average duration of the treatment ranges from 7 to 9 months. At the removal of the last plaster corset, the patient is attended to wear an orthopaedic one, most frequently a Lyonnese: this second part of the treatment continues until the conclusion of the bone maturity. The correct indication for the Risser plaster corsets are angular curve between 35 and 50 Cobb degrees, in patient with an high risk of scoliosis' progression.

**Materials and methods** This study is a unicentric retrospective cases series. Level of evidence is V. One hundred and eighty-three patients were included in this study. Of these, 155 (84.69 %) were females, 28 (15.3 %) were males: F/M ratio was 5.5. The mean age at the treatment's beginning was 12.3 years (range 9–16 years). Concerning bone maturity, described according to Risser Test, average level was 1.8 (range 0–4). The mean duration of the plaster corset treatment was 225 days (range 135–282 days). The mean clinical-radiological follow-up, after the last corset removal, was 303 days (range 267–389).

**Results** The main scoliotic curves had an average Cobb angle of 40.3° (range 37°–52°) and 32°(range 19°–38°) at the beginning and at the conclusion of the treatment respectively. The mean correction ratio was 17 % (range 5–31 %). At the outpatient follow up, 157 (85.8 %) patients did not present a significant curve relapse, while in the remaining 26 (14.2 %) a clinical and radiological curve progression was observed. In this phase, the mean Cobb angle of the main curves was 36°(range 20°–46°), with a correction ratio of 11.8 % (range 8–21 %).

**Discussion** In literature there is not a common consensus about the use of plaster corsets in the treatment of adolescent idiopathic scoliosis. The most recent papers advise and encourage their use upon corrected indications and motivated patients. Our study agrees with these last statements, validated by an high success rate (85.8 %) and by a low risk of complication (1.06 %). Moreover, as handed down by the history, plaster corsets have much more correction index than orthopaedic ones.

**Conclusions** In conclusion, in our study, the conservative treatment according to Risser's plaster corsets remains even now efficient, useful and mostly precious for the treatment of the adolescent idiopathic scoliosis with angular values ranging between 35 and 50 degrees.

### Safety and efficacy of the shoulder pain treatment by using infiltrations of methylprednisolone acetate in diabetic patients

D. Blonna[1], L. Mattei*[1], N. Barbasetti del Prun[1], F. Fissore[1], R. Rossi[1], D.E. Bonasia[2], A. Marmotti[1], F. Castoldi[1]

[1]Divisione Universitaria di Ortopedia e Traumatologia, AO Mauriziano "Umberto I", Università di Torino, (Turin, IT); [2]Prima Clinica Ortopedica, Città della Salute e della Scienza, Università di Torino, AO CTO Maria Adelaide (Turin, IT)

**Introduction** Cortisone subacromial infiltration is an effective short-term treatment for shoulder pain and could be potentially useful in diabetic patients. The risk–benefit ratio of an infiltration in diabetic patients is not yet known. The aim of our study is to assess the risk–benefit ratio of subacromial infiltration with cortisone in diabetic patients.
**Materials and methods** Thirty diabetic patients with shoulder resistant pain to 6 months of conservative treatment were included in the study. The inclusion criteria were: severe night pain (VAS $\geq$ 7), manifestations of subacromial bursitis, absence of severe glenohumeral arthrosis. Patients were asked to monitor their glycemia 4 times a day for a week. Patients with good glycemic control (glycemia < 300 mg or glycated hemoglobin < 7.5 %) have been infiltrated in subacromial space with methylprednisolone acetate 40 mg (depomedrol) and lidocaine 2 % 3 ml. Glycemia was measured 7 days later. The effectiveness evaluation was performed by using constant score (CS), oxford shoulder score (OSS), subjective outcome determination score (SOD) with 1 month and 1 year follow-up. Statistical analysis was performed using Student's *t* test and Fisher's test.
**Results** Of the 30 patients initially included, 20 completed the study (7 were excluded because of not controlled glycemia and 3 were lost at follow-up). At the 30 days control, the patients reported an improvement (SOD score = 3) with significant improvement in both CS and OSS (CS from 43 to 52, $p < 0.05$; OSS from 28 to 32, $p < 0.01$). At one year follow-up, OSS and SOD scores were improved compared to the 30 days follow-up. In first day fasting glycemia increased from 125 to 154 mg/dl ($p < 0.01$). Mean values were normalized within 7 days from infiltration. Four patients had a isolated post-prandial glycemia > 300 mg/dl. No difference was observed between patients under subcutaneous insulin therapy or under oral hypoglycemic therapy.
**Discussion** The improvement was mainly due to day and night pain reduction. The infiltration revealed to be safe in all patients.
**Conclusions** In properly selected patients, subacromial infiltration with acetate methylprednisolone, revealed to be safe and effective in reducing pain as a short term treatment option in non-responsive patients to conservative treatment.

### Pedicle fenestrated screws and pedicle expanding screws in spine surgery: our experience

L. Babbi*, S. Bandiera, S. Colangeli, A. Gasbarrini, G. Barbanti, S. Terzi, R. Ghermandi, S. Boriani

Istituto Ortopedico Rizzoli (Bologna, IT)

**Introduction** Pedicle screws positioning are nowadays the gold standard in spine surgery arthrodesis. Osteoporosis is the main cause of hardware failure in elderly population and new treatment options are continuously been investigated. Different systems have been designed to avoid aseptic loosening of screws: fenestrated screws, expanding screws, hydroxyapatite covered screws. We present our experience with fenestrated and expanding pedicle screws for posterior stabilization in selected patients.
**Materials and methods** From September 2006 to February 2012, 90 patients underwent fenestrated (83 patients) and expanding (8 patients) pedicle screws positioning. The diagnosis were traumatic fractures in osteoporotic bone, tumors, post traumatic deformity, degenerative discopathy, infectious spondylodiscitis, fractures in coagulopathy and revision surgery in hardware failure. Three hundreds and thirty-five cemented fenestrated screws and 36 expanding screws were finally placed.
**Results** After an average follow-up of 12 months (2–35 months) we registered 6 complications in the group of patients treated with cemented fenestrated screws: leakage of cement into the canal in 4 patients (4.3 %) was showed at postoperative CT scan (1 patient had a reversible peripheral nerve deficit that healed after 14 months of physiotherapy). In one case, intraoperative fluoroscopy showed a leakage of the cement into the canal; this patient was submitted to a laminectomy in order to remove the cement and no post-operative neurological deficits were detected. Finally we had one case of 2 distal screws loosening in a patient with multiple myeloma treated with thoraco-lumbar stabilization after oncological therapy (follow-up 11 months). At a mean 1.5-months follow-up no complication was observed in the group of patients treated with expanding screws.
**Discussion** Fenestrated screws placement offers better mechanical stability than conventional screws, but risks of cement leakage can occur as we described; in our series of patients no clinical complications occured.
**Conclusions** Fenestrated screws are recommended in selected cases were bone quality is considerably low because, despite a longer surgical procedure, they can reduce the re-intervention rate due to screws failure. Our initial expanding screws employment, according to encouraging biomechanical evidences, seems enhance bone anchoring in osteoporotic bone and improve patients outcome in spinal fusion surgery; additional results are required.

### Acetabular reconstruction with the Burch-Schneider ring: outcomes at 18 months

D. Vanni*, A. Pantalone, T. Scuccimarra, A. Rossetti, M. Borri, A. D. Apolito, C. Colucci, V. Salini

Clinica Ortopedica e Traumatologica (Chieti, IT)

**Introduction** Acetabular component revision is a real challenge for orthopedic surgeons. A pre-operative bone stock and bone defect detailed evaluation is a crucial point. In order to obtain an absolute stability, the natural or synthetic graft have to restore, over time, the depleted bone stock (Winter E. et al., J Bone Joint Surg Am 2001).
**Materials and methods** The Burch-Schneider acetabular ring provides a large contact surface between the acetabular component and the bone, allowing to the load forces to be distributed along the ileum and ischio-pubic branches (Kawanabe K. et al., J Arthroplasty 2011). From January 2009 to January 2012, 10 patients (mean age: 71 years) with prosthetic acetabular component loosening, underwent to a revision surgery using Burch-Schneider acetabular ring. All patients were pre-operatively classified according to Paprosky score. A clinical (Harris Hip Score and Merle Daubigné Score) and radiographic follow-up (Benson and Gill Classification) was performed at 1, 3, 6, 12 and 18 months (Regis D. et al., J Arthroplasty 2012).
**Results** To date, no patients needed to a secondary revision surgery and there were no cases of infection. During the follow-up, no radiographic signs of early mobilization were detected. The bone grafts, natural or synthetic, were integrated with a gradual bone stock recovery.
**Discussion** Life expectancy increase and first implant earlier age are responsible for a progressive growth revision rate. The early mobilization and the bone stock gradual recovery could be achieved only through the absolute stability of the implant and the appropriate prosthetic components (Tidermark J. et al., J Orthop Trauma 2003). Therefore, the bone substitutes have only a bone-restorer role (Vanni D. et al., Int J Immunopathol Pharmacol 2011).

**Conclusions** The Burch-Schneider acetabular ring is effective. It allows the patient early mobilization. This implant represent a viable alternative in prosthetic acetabular component revisions associated with serious bone defects, preventing the secondary mobilization, especially in patients with poor bone stock (Ilchmann T. et al., Acta Orthop 2006).

### PRP+ microfractures: prospective randomized study for the treatment of chondral lesions

A. Mancò*[1], R. Goderecci[2], A. Rughetti[3], V. Calvisi[1]

[1]Scuola di Specializzazione in Ortopedia e Traumatologia, Università degli Studi dell'Aquila (L'Aquila, IT);
[2]CLM in Medicina e Chirurgia, Università degli Studi dell'Aquila (L'Aquila, IT);
[3]Servizio Immunotrasfusionale, ASL 1, Ospedale Civile San Salvatore (L'Aquila, IT)

**Introduction** Over the years, in the treatment of chondral lesions of the knee, many surgical techniques (arthroscopic or not) tried to find the best clinical and histological outcome. Seeing the clinical efficacy of bone marrow stimulation techniques and the regenerative properties of platelet rich plasma on cartilage tissue, we set out to evaluate the clinical efficacy of this particular arthroscopic treatment compared to the same treatment associated with an intra-articular activated-PRP injection; particularly our interest was to evaluate the improvement of life quality in patients still young for an early prosthetic replacement.
**Materials and methods** It's a prospective randomized study in which we compared two groups of patients affected by chondral lesions grade IV according to the Outerbridge classification, aged between 40 and 60 years, respectively subjected to a specific arthroscopic treatment (microfractures according to Steadman/abrasion arthroplasty) or to the same arthroscopic treatment associated with the intra-articular use of autologous PRP. Clinical efficacy was also assessed objectively by VAS Scale, SF-36 and IKDC to compare preoperative and post-operative values at 3 week, 3 months and 6 months.
**Results** In the post-operative evaluation, improved clinical outcomes in the PRP group—which was confirmed by the results in objective tests VAS, SF-36 and IKDC at 3 weeks, 3 months and 6 months—is in line with the literature.
**Discussion** The arthroscopic technique of microfractures described by Steadman, for its simplicity and low cost of execution, is one of most used. It bases on bone marrow stimulation (MSCs) followed by a formation of fibrocartilage repair tissue. The recent and increasing evidences regarding the potential efficacy of PRP on chondral lesions, both biological in vitro (e.g.: effects on proliferation and chondrogenic differentiation of the MSCs) and clinical in vivo, confirmed the hypothesis of its valid association with the arthroscopic treatment.
**Conclusions** In our results, and mainly in the early follow-up, the use of autologous PRP in association with the arthroscopic microfractures seems to be a good association to a clinical and functional improvement in the reparative treatment of chondral lesions; given the exiguity of samples evaluated and the brief follow-up, these data have to be considered certainly preliminary.

### Treatment of nonunion of the forearm bones with posterior interosseous bone flap

R. Sorbi, R. S. Kamrani, M. R. Aghamirsalim

Department of Orthopaedic and Trauma Surgery, Shariati Hospital, Students' Scientific Research Center (SSRC), Tehran University of Medical Sciences (TUMS) (Tehran, IRAN)

**Objective** Reconstruction of forearm fracture nonunions are frequently complex. A few studies have been reported to help guide the treatment of forearm non-union [1, 2]. We offer a novel surgical technique to treat nonunion of the forearm using a regional vascularized bone graft.
**Materials and methods** Four females and five males ranging from 27 to 74 years of age with 7 ulna and 2 radius nonunions were surgically treated by pedicled grafting with a posterior interosseous bone flap (PIBF) and internal fixation with a plate. There were no cases with extensive soft tissue damage or infection. Pre- and post-operatively (mean: 21 months), all patients were assessed by radiographs and for function by the disabilities of the arm, shoulder and hand (DASH) score.
**Results** In all patients, function of the upper extremity was improved. DASH scores improved from 61.2 points preoperatively to 12 points at final follow up. All fractures were united uneventfully.
**Conclusions** Following debridement of the necrotic tissue, the bone defect can be filled with a vascularized graft from posterior interosseous pedicles. Pedicled PIBF is a safe and useful novel technique in cases of atrophic or hypertrophic nonunion of the middle third of the radius or proximal two-thirds of the ulna.

**References**
1. Moroni A, Rollo G, Guzzardella M, Zinghi G (1997) Surgical treatment of isolated forearm non-union with segmental bone loss. Injury 28:497–504.
2. Safoury Y (2005) Free vascularized fibula for the treatment of traumatic bone defects and nonunion of the forearm bones. J Hand Surg Br 30:67–72
